# Global burden of calcific aortic valve disease and attributable risk factors from 1990 to 2019

**DOI:** 10.3389/fcvm.2022.1003233

**Published:** 2022-11-23

**Authors:** Jiaye Yu, Zhuo Wang, Qinyi Bao, Shuxin Lei, Yayu You, Zhehui Yin, Xiaojie Xie

**Affiliations:** Department of Cardiology, The Second Affiliated Hospital, Zhejiang University School of Medicine, Hangzhou, Zhejiang, China

**Keywords:** calcific aortic valve disease, Global Burden of Disease Study, disability-adjusted life year, estimated annual percentage change, attributable risk factor

## Abstract

**Background:**

Calcific aortic valve disease (CAVD) was highly prevalent among developed countries and caused numerous deaths. Based on the Global Burden of Disease 2019, this study was designed to present comprehensive epidemiological information, attributable risks, and relevant factors.

**Methods:**

All data were available online *via* the Global Health Data Exchange (GHDx). In this study, we analyzed the global incidence, prevalence, deaths, and disability-adjusted life years (DALYs) of CAVD across different regions from 1990 to 2019. We applied the estimated annual percentage changes (EAPCs) to evaluate the change trends and their attributable risks. In addition, we explored several relevant factors.

**Results:**

From 1990 to 2019, the incidence cases, prevalence cases, CAVD-related deaths, and DALYs of CAVD gradually increased globally. However, the age-standardized death rate (ASDR) was relatively stable, and the age-standardized DALYs rate gradually declined during the past 30 years. Males and elderly individuals were more likely to suffer from CAVD. High systolic blood pressure (SBP) was the predominant attributable risk of disease burden that presented a global downward trend (death: EAPC = −0.68, 95% CI −0.77~−0.59, *P* < 0.001; DALYs: EAPC = −0.99, 95% CI −1.09 to −0.89, *P* < 0.001). Alcohol consumption (R = 0.79, *P* < 0.001), smoking prevalence (R = 0.75, *P* < 0.001), and calcium (*R* = 0.72, *P* < 0.001) showed a positive correlation with the age-standardized incidence rate (ASIR), whereas classic monsoon region (*R* = −0.68, *P* < 0.001) and mean temperature (*R* = −0.7, *P* < 0.001) showed a negative correlation with age-standardized incidence rate (ASIR). Besides, medical and healthcare resources presented a positive correlation with ASIR. Meanwhile, similar relationships were found in age-standardized prevalence rate (ASPR), ASDR, and age-standardized DALY rate (ASDALYR).

**Conclusion:**

CAVD displays widely varied spatial distribution around the world, of which high SDI regions have the highest burdens. Age is a powerful factor and hypertension a predominant attributable risk factor. Moreover, controlling blood pressure, avoiding smoking, reducing alcohol consumption, and so on, could effectively reduce the burden of CAVD.

## Introduction

Calcific aortic valve disease (CAVD) is defined as a clinical diagnosis of stenosis due to progressive calcification of the valve which causes impaired hemodynamics. Much clinical and laboratory evidence has suggested that the disease was an active process involving chronic inflammation, lipid deposition, and biomineralization (1). Moreover, the clinical evolution of the disease is from aortic sclerosis and asymptomatic aortic stenosis (AS) to symptomatic aortic stenosis. When the valve becomes thickened over the years, it presents severely impaired leaflet motion and vast left ventricular outflow tract obstruction, which causes left ventricular remodeling (1). Finally, thisresults in chronic heart failure.

CAVD is the most common valvular heart disease (VHD) in the western world and high-income countries (2). Furthermore, it is the third most frequent cardiovascular disease after coronary artery disease and hypertension among developed countries (3). The prevalence of CAVD rose sharply with age, especially in those aged over 65 years, and most of those were symptomatic (1). In 2017, there were ~12.6 million cases of CAVD, increased by 124% from 1990, with 102,700 CAVD-related deaths globally (2). Moreover, rheumatic heart disease (RHD), the most prevalent VHD in the world, showed a downward trend, whereas CAVD presented the fastest increase among non-rheumatic valvular disease (NRVD).

Since life expectancy has extended over the last decades, the prevalence of CAVD was predicted to double within the next half century (4). And as no medication has proved to be effective in stopping the evolution of this disease, aortic valve replacement (AVR) is the only effective therapy for it, which remains a significant financial and physical burden for patients (5, 6). Therefore, more attention should be paid to the existing high burden of CAVD, and prevention is particularly important and cost-effective. In this study, we focused on the global incidence, prevalence, deaths, and disability-adjusted life years (DALYs) of CAVD across different regions (204 countries and territories) from 1990 to 2019. Moreover, we analyzed attributable risks and various covariates to discover underlying attributable risk factors and potential exposure.

## Methods

### Study data

All data were available in the Global Health Data Exchange (GHDx), an online catalog conducted by the Institute for Health Metrics and Evaluation (IHME). GBD 2019 study estimated epidemiological quantity in 23 age groups, gender groups, and 204 countries and territories for a total of 369 diseases (7, 8). In this study, we extracted the incidence, prevalence, deaths, DALYs, and corresponding age-standardized rates (ASRs) of CAVD and health risk factors attributable to it. We presented the above indicators for 5 socio-demographic index (SDI) regions, 4 World Bank income (WBI) level groups, 21 GBD regions, and 204 countries and territories from 1990 to 2019. Meanwhile, we performed gender and age stratifications. SDI is a socio-demographic indicator comprehensively considering education, national income, and fertility rate (7, 9). Thus, the world is divided into five SDI regions including low, low-middle, middle, high-middle, and high SDI regions. According to gross national income (GNI) per capita calculated by the World Bank Atlas method, there are four WBI regions including low, lower-middle, upper-middle, and high WBI regions (10).

DALYs are the sum of years of life lost (YLLs) (due to premature mortality) and years lived with disability (YLDs). In brief, YLDs comprehensively consider several social preference values, for example, disability weight, age weight, and so on. And the formula to calculate DALYs could be simplified as


$$\begin{array}{lll} \text{ YLL} & = & N\times L1\\ \text{ YLD} & = & I\times D\times L2\\ \text{ DALYs} & = & YLLs+YLDs \end{array}$$

Where N is the number of premature deaths due to a given disease; L1 is the standard life expectancy loss for each death; I is the number of disabilities; D is thedisability weight; and L2 is the average duration of disease (11).

In addition, the GBD 2019 study provided 87 risk factors for a given disease at regional levels. Three attributable risks of CAVD were found, including lead exposure, high systolic blood pressure (SBP), and a diet high in sodium. The population attributable fraction (PAF) is the estimated fraction of cases that would be attributable to exposure. And the computational formula of PAF is presented as:

PAF = E/O × 100% (where O and E refer to the observed case load and the case load attributable to exposure, respectively) (7, 12). Finally, we analyzed the burden of CAVD and covariates downloaded from the GBD 2019 covariate dataset (https://cloud.ihme.washington.edu/s/b2tQnbsjAyWgeHm?path=%2FGBD%202019%20Covariates), and correlation coefficient (R-value) was calculated for each covariate.

### Statistical analysis

Annual incidence cases, prevalence cases, CAVD-related death cases, and DALYs were used to present the disease burden. Meanwhile, corresponding ASRs were applied to exclude the age distribution differences among different populations (13). The estimated annual percentage change (EAPC) of ASR of incidence, prevalence, deaths, and DALYs were used to reflect the trends of disease burden. Based on the equation Y = α + βX + ε [where Y refers to ln(ASR), X refers to the calendar year, and ε represents the error term], EAPC is calculated using the formula EAPC = 100 × [exp(β) – 1] (14). Therefore, when the EAPC value and its 95% confidence interval (CI) are over zero, the ASR presents an uptrend and vice versa (15). Furthermore, we calculated spearman's correlation coefficient (R) to explore the correlation between the burden of CAVD (using ASRs) and covariates. Data analysis and visualization were performed by the open-source software R (version 4.1.0). A two-tailed *p* < 0.05 was deemed statistically significant.

## Results

### The incidence and its trend

**Figure 1:**
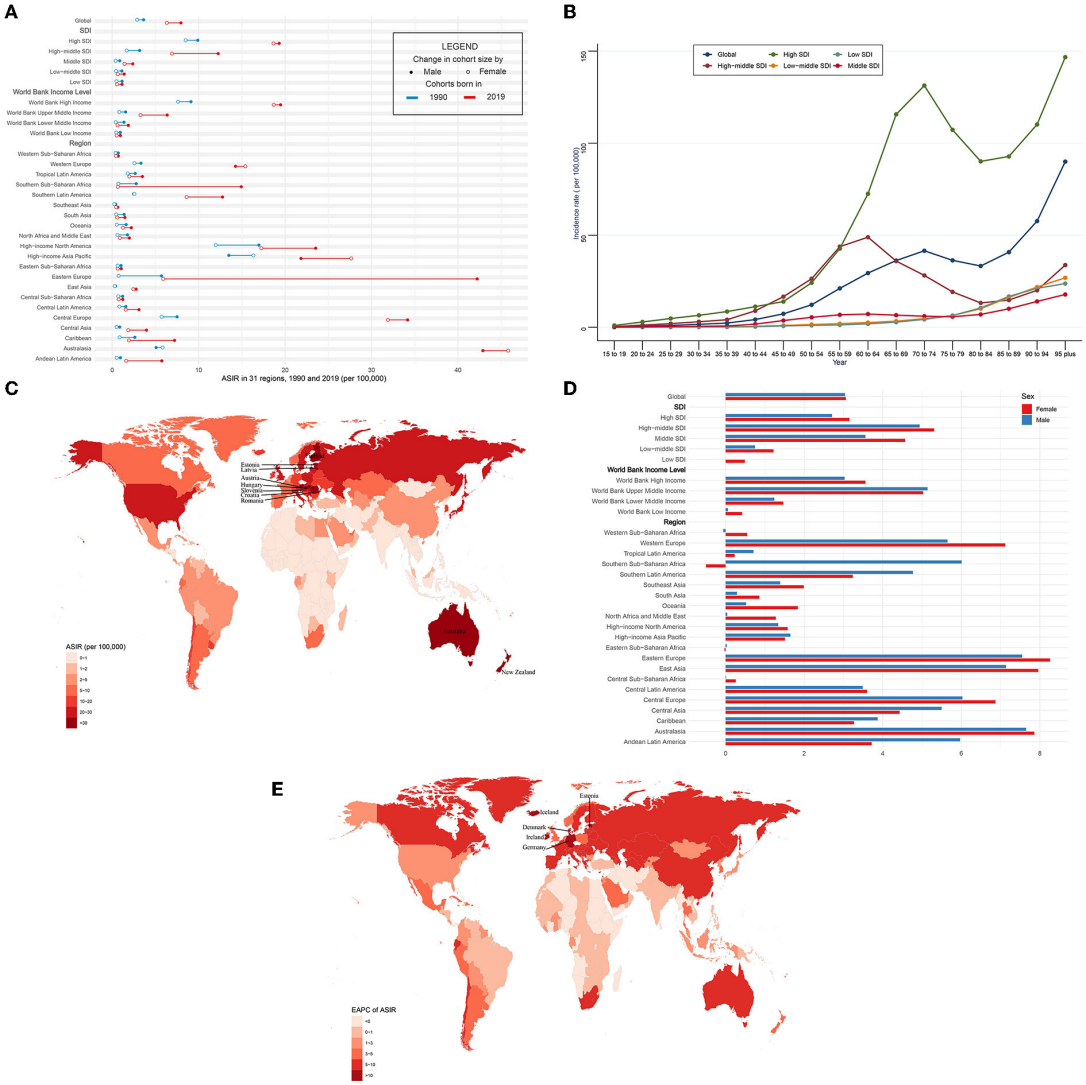
ASIR and its trend of CAVD. **(A)** ASIR in 31 regions from 1990 to 2019. **(B)** Incidence rate stratified by age in the globe and 5 SDI regions in 2019. **(C)** ASIR in 204 countries and territories in 2019. **(D)** EAPC of ASIR in 31 regions from 1990 to 2019. **(E)** EAPC of ASIR in 204 countries and territories from 1990 to 2019. CAVD, calcific aortic valve disease; ASIR, the age-standardized incidence rate; SDI, socio-demographic index; EAPC, estimated annual percentage change.

Globally, over the past 30 years, the incidence case of CAVD gradually increased by 351% from 130,821 in 1990 to 589,637 cases in 2019 (Table 1). Meanwhile, the age-standardized incidence rate (ASIR) of CAVD increased by 120% from 3.25 (95% UI 2.76~3.86) per 100,000 in 1990 to 7.13 (95% UI 6.22~8.15) in 2019 (EAPC = 3.03, 95% CI 2.80~3.27) (Tables 1, **5**). In the socio-demographic factor level, the high SDI region had the highest CAVD burden until 2019 [incidence case: 92,683 in 1990 and 329,823 cases in 2019; ASIR: 9.18 (95% UI 7.65~11.05) per 100,000 in 1990 and 19.04 (95% UI 16.65~21.96) in 2019; EAPC = 2.93, 95% CI 2.62~3.23] (Tables 1, **5**). At the same time, high-middle SDI had the fastest increase in the 30 years (ASIR: 2.40 per 100,000 in 1990 and 9.59 in 2019, EAPC = 5.10, 95% CI 4.92~5.27) (Tables 1, **5**). Similar results were found at the WBI level. Among geographical zones, high-income north America and high-income Asia Pacific had the highest ASIR in 1990 (14.32 and 15.12 per 100,000, respectively) while central Europe and Australasia presented the highest ASIR in 2019 (33.16 and 44.39 per 100,000, respectively) (Table 1). Australasia and eastern Europe were the fastest-growing regions (EAPC of Australasia: 7.72, 95% CI 7.51~7.94; EAPC of Eastern Europe: 7.75, 95% CI 7.33~8.16) (**Table 5**). Among 204 countries and territories, the USA and Japan had the highest ASIR in 1990 (15.77 and 17.10 per 100,000, respectively), while Hungary and Slovenia had the highest ASIR in 2019 (56.24 and 62.21 per 100,000, respectively) (Figure 1C; Supplementary Table 1). Germany and Iceland had the fastest increase in ASIR (EAPC = 12.84, 95% CI 10.89~14.82 and 13.35, 95% CI 10.93~15.81, respectively) (Figure 1E; Supplementary Table 2). Incidence was positively correlated with age and there were two peaks, one at age 70 to 74 and the other at age over 95 (Figure 1B).

**Table 1:** The incidence of CAVD in 1990/2019.

	**1990**						**2019**					
	**Incident cases No** *10^**3**^ **(95% UI)**			**ASIR/100,000 No. (95% UI)**			**Incident cases No** *10^**3**^ **(95% UI)**			**ASIR/100,000 No. (95% UI)**		
	**Male**	**Female**	**Both**	**Male**	**Female**	**Both**	**Male**	**Female**	**Both**	**Male**	**Female**	**Both**
Global	69.64 (59.32~82.58)	61.18 (50.97~73.84)	130.82 (110.7~156.02)	3.63 (3.11~4.27)	2.88 (2.41~3.45)	3.25 (2.76~3.86)	313.8 (271.31~360.92)	275.83 (239.87~317.14)	589.64 (512.9~677.06)	7.95 (6.92~9.09)	6.31 (5.49~7.26)	7.13 (6.22~8.15)
SDI level												
High SDI	44.82 (37.39~53.93)	47.86 (39.24~58.27)	92.68 (76.42~112.27)	9.9 (8.32~11.84)	8.49 (7~10.28)	9.18 (7.65~11.05)	153.12 (133.19~177.54)	176.7 (152.55~204.1)	329.82 (285.98~381.25)	19.31 (16.87~22.23)	18.66 (16.22~21.66)	19.04 (16.65~21.96)
High-middle SDI	16.38 (13.68~19.36)	10.01 (8.51~11.81)	26.39 (22.29~31.01)	3.17 (2.69~3.72)	1.68 (1.42~1.97)	2.4 (2.04~2.79)	118.39 (98.87~139.91)	74.82 (64.36~86.77)	193.22 (163.54~225.65)	12.25 (10.28~14.34)	6.92 (5.94~8.03)	9.59 (8.14~11.18)
Middle SDI	4.28 (3.62~5.08)	1.91 (1.59~2.32)	6.19 (5.24~7.33)	0.87 (0.75~1.01)	0.4 (0.34~0.48)	0.63 (0.54~0.73)	30.87 (25.84~36.65)	19.07 (15.79~23.07)	49.94 (41.59~59.44)	2.38 (2.01~2.78)	1.42 (1.19~1.69)	1.89 (1.59~2.23)
Low-middle SDI	2.93 (2.49~3.43)	0.98 (0.81~1.18)	3.91 (3.33~4.61)	1.11 (0.96~1.29)	0.44 (0.36~0.52)	0.77 (0.66~0.9)	8.58 (7.37~10)	4.02 (3.41~4.78)	12.6 (10.82~14.6)	1.38 (1.19~1.59)	0.62 (0.53~0.74)	0.98 (0.85~1.14)
Low SDI	1.21 (1.04~1.42)	0.41 (0.34~0.48)	1.62 (1.39~1.88)	1.14 (1~1.3)	0.49 (0.42~0.58)	0.82 (0.71~0.94)	2.72 (2.35~3.18)	1.14 (0.97~1.34)	3.86 (3.34~4.46)	1.14 (0.99~1.31)	0.56 (0.48~0.65)	0.84 (0.73~0.97)
World Bank Income Level												
World Bank High Income	50.53 (42.41~60.72)	52.74 (43.46~64)	103.28 (85.7~124.63)	9.11 (7.7~10.89)	7.62 (6.32~9.2)	8.35 (6.99~10.01)	181.82 (158.47~210.31)	208.85 (179.93~241.29)	390.67 (339.45~451.2)	19.48 (17.02~22.4)	18.68 (16.26~21.64)	19.14 (16.73~22.05)
World Bank Upper Middle Income	11.75 (9.86~13.89)	6.39 (5.45~7.55)	18.14 (15.35~21.22)	1.54 (1.32~1.79)	0.82 (0.7~0.96)	1.18 (1.01~1.37)	108.78 (89.85~129.14)	59.39 (49.83~70.34)	168.17 (139.25~198.69)	6.36 (5.28~7.49)	3.27 (2.75~3.85)	4.81 (4~5.65)
World Bank Lower Middle Income	6.73 (5.73~7.87)	1.77 (1.45~2.19)	8.5 (7.24~9.96)	1.36 (1.16~1.57)	0.4 (0.34~0.49)	0.87 (0.74~1.01)	21.73 (18.47~25.39)	6.82 (5.76~8.11)	28.55 (24.32~33.44)	1.87 (1.6~2.16)	0.61 (0.52~0.72)	1.22 (1.05~1.41)
World Bank Low Income	0.61 (0.53~0.71)	0.26 (0.22~0.31)	0.87 (0.76~1.01)	0.93 (0.83~1.04)	0.46 (0.4~0.53)	0.69 (0.61~0.77)	1.36 (1.18~1.58)	0.69 (0.6~0.81)	2.05 (1.79~2.36)	0.94 (0.84~1.07)	0.51 (0.45~0.59)	0.72 (0.64~0.81)
Region												
Andean Latin America	0.1 (0.09~0.11)	0.05 (0.05~0.06)	0.15 (0.13~0.17)	0.95 (0.84~1.06)	0.51 (0.45~0.59)	0.72 (0.65~0.81)	1.66 (1.43~1.91)	0.49 (0.41~0.57)	2.15 (1.86~2.46)	5.74 (4.98~6.62)	1.62 (1.37~1.9)	3.62 (3.14~4.16)
Australasia	0.49 (0.4~0.59)	0.77 (0.68~0.88)	1.26 (1.1~1.45)	5.07 (4.27~6)	5.83 (5.15~6.55)	5.56 (4.9~6.32)	9.23 (7.82~10.95)	10.95 (9.07~13.09)	20.17 (17.13~23.51)	42.87 (36.65~50.79)	45.8 (38.35~54.49)	44.39 (38.08~51.79)
Caribbean	0.33 (0.3~0.37)	0.11 (0.1~0.13)	0.44 (0.4~0.49)	2.62 (2.36~2.9)	0.85 (0.75~0.96)	1.7 (1.53~1.88)	1.82 (1.55~2.15)	0.53 (0.46~0.61)	2.35 (2.02~2.73)	7.21 (6.13~8.44)	1.94 (1.67~2.23)	4.49 (3.86~5.21)
Central Asia	0.19 (0.15~0.23)	0.14 (0.11~0.18)	0.33 (0.27~0.4)	0.87 (0.72~1.04)	0.5 (0.41~0.63)	0.66 (0.54~0.8)	1.74 (1.43~2.06)	0.9 (0.73~1.07)	2.65 (2.18~3.11)	3.97 (3.28~4.66)	1.87 (1.54~2.21)	2.86 (2.37~3.35)
Central Europe	5.11 (4.3~6.03)	4.69 (3.92~5.52)	9.8 (8.26~11.5)	7.5 (6.34~8.77)	5.71 (4.78~6.71)	6.59 (5.52~7.69)	27.88 (23.58~32.92)	29.4 (25.02~34.63)	57.28 (48.87~67.32)	34.18 (28.97~40.11)	31.88 (27.15~37.4)	33.16 (28.29~38.67)
Central Latin America	0.69 (0.6~0.79)	0.36 (0.31~0.41)	1.05 (0.92~1.2)	1.57 (1.37~1.79)	0.82 (0.72~0.94)	1.19 (1.05~1.35)	3.7 (3.19~4.26)	2.05 (1.75~2.39)	5.75 (4.95~6.62)	3.09 (2.67~3.55)	1.57 (1.35~1.82)	2.29 (1.99~2.63)
Central Sub-Saharan Africa	0.11 (0.1~0.13)	0.05 (0.04~0.06)	0.16 (0.14~0.19)	1.21 (1.06~1.37)	0.7 (0.62~0.8)	0.94 (0.84~1.06)	0.25 (0.22~0.3)	0.16 (0.14~0.18)	0.41 (0.36~0.47)	1.22 (1.08~1.38)	0.76 (0.66~0.85)	0.97 (0.87~1.1)
East Asia	1.72 (1.37~2.18)	1.25 (0.98~1.59)	2.97 (2.37~3.78)	0.4 (0.32~0.49)	0.29 (0.23~0.36)	0.34 (0.28~0.42)	29.75 (23.71~36.34)	26.89 (21.55~33.19)	56.64 (45.3~69.33)	2.77 (2.24~3.32)	2.44 (1.97~2.99)	2.61 (2.11~3.15)
Eastern Europe	6.67 (5.26~8.16)	1.27 (1.02~1.57)	7.93 (6.36~9.59)	5.7 (4.57~6.89)	0.73 (0.6~0.89)	2.9 (2.34~3.48)	57.17 (45.96~69.72)	11.24 (9.06~13.65)	68.42 (55.23~82.91)	42.22 (34.12~50.83)	5.91 (4.74~7.11)	22.58 (18.38~27.04)
Eastern Sub-Saharan Africa	0.34 (0.29~0.39)	0.16 (0.14~0.19)	0.5 (0.43~0.57)	1.02 (0.9~1.15)	0.64 (0.55~0.74)	0.83 (0.74~0.95)	0.77 (0.65~0.91)	0.4 (0.34~0.47)	1.17 (1.01~1.35)	1.05 (0.91~1.2)	0.64 (0.54~0.74)	0.84 (0.73~0.96)
High-income Asia Pacific	12.34 (10.17~14.87)	18.14 (14.91~22.22)	30.47 (25.25~36.92)	13.49 (11.28~16.05)	16.33 (13.56~19.81)	15.12 (12.61~18.17)	33.55 (27.93~40.84)	55.05 (45.3~66.38)	88.6 (73.61~107.12)	21.84 (18.59~25.63)	27.65 (23.31~33.08)	25.04 (21.32~29.6)
High-income North America	25.9 (21.23~31.6)	23 (18.51~28.36)	48.91 (39.76~59.97)	16.97 (13.99~20.58)	11.97 (9.74~14.71)	14.32 (11.71~17.4)	64.38 (55.43~74.66)	59.19 (50.96~68.17)	123.57 (106.45~142.33)	23.54 (20.52~26.98)	17.26 (14.98~19.67)	20.23 (17.61~23.2)
North Africa and Middle East	1.6 (1.36~1.87)	0.43 (0.37~0.52)	2.04 (1.75~2.37)	1.76 (1.53~2.03)	0.6 (0.52~0.71)	1.19 (1.04~1.36)	5.25 (4.37~6.23)	1.82 (1.57~2.13)	7.07 (5.98~8.35)	1.98 (1.69~2.31)	0.89 (0.78~1.02)	1.46 (1.27~1.69)
Oceania	0.02 (0.02~0.03)	0.01 (0.01~0.01)	0.03 (0.02~0.03)	1.61 (1.35~1.93)	0.53 (0.46~0.6)	1.07 (0.91~1.26)	0.09 (0.07~0.1)	0.04 (0.04~0.05)	0.13 (0.11~0.15)	2.21 (1.86~2.63)	1.24 (1.04~1.45)	1.73 (1.47~2.04)
South Asia	3.33 (2.8~3.94)	0.8 (0.65~0.99)	4.13 (3.48~4.94)	1.37 (1.16~1.6)	0.46 (0.38~0.55)	0.93 (0.79~1.09)	9.03 (7.66~10.6)	3.34 (2.74~4.06)	12.37 (10.49~14.62)	1.47 (1.25~1.72)	0.57 (0.47~0.68)	1.01 (0.85~1.18)
Southeast Asia	0.44 (0.36~0.53)	0.28 (0.22~0.34)	0.71 (0.59~0.86)	0.41 (0.35~0.48)	0.24 (0.2~0.29)	0.32 (0.27~0.38)	1.88 (1.56~2.24)	1.42 (1.2~1.71)	3.29 (2.78~3.94)	0.64 (0.55~0.75)	0.45 (0.38~0.53)	0.54 (0.46~0.64)
Southern Latin America	0.47 (0.4~0.55)	0.62 (0.49~0.92)	1.09 (0.9~1.43)	2.48 (2.1~2.87)	2.57 (2.03~3.86)	2.59 (2.13~3.46)	4.67 (4.07~5.45)	4.06 (3.57~4.63)	8.73 (7.73~9.92)	12.76 (11.17~14.78)	8.6 (7.56~9.83)	10.64 (9.44~12.08)
Southern Sub-Saharan Africa	0.34 (0.29~0.41)	0.1 (0.08~0.12)	0.44 (0.37~0.52)	2.78 (2.33~3.35)	0.72 (0.6~0.85)	1.62 (1.38~1.91)	4.45 (3.54~5.51)	0.18 (0.15~0.22)	4.64 (3.72~5.7)	14.93 (12.01~18.1)	0.65 (0.54~0.77)	7.09 (5.73~8.6)
Tropical Latin America	1.17 (1~1.37)	0.78 (0.66~0.91)	1.95 (1.67~2.28)	2.66 (2.26~3.08)	1.79 (1.51~2.1)	2.22 (1.91~2.57)	3.95 (3.35~4.64)	2.64 (2.24~3.09)	6.59 (5.61~7.68)	3.5 (2.97~4.09)	1.99 (1.69~2.32)	2.71 (2.31~3.15)
Western Europe	7.92 (6.58~9.54)	8.03 (6.56~9.87)	15.95 (13.21~19.24)	3.33 (2.79~3.97)	2.56 (2.11~3.14)	2.96 (2.46~3.57)	51.77 (43.75~61.18)	64.74 (54.65~77.31)	116.51 (98.95~138.35)	14.25 (12.15~16.77)	15.41 (13.15~18.28)	14.9 (12.77~17.56)
Western Sub-Saharan Africa	0.36 (0.3~0.43)	0.14 (0.11~0.17)	0.51 (0.43~0.6)	0.7 (0.6~0.83)	0.38 (0.32~0.46)	0.57 (0.49~0.66)	0.81 (0.68~0.97)	0.35 (0.29~0.42)	1.16 (0.99~1.37)	0.71 (0.61~0.84)	0.45 (0.38~0.53)	0.58 (0.5~0.67)

CAVD, calcific aortic valve disease; ASIR, the age-standardized incidence rate; SDI, socio-demographic index.

The ASIR was higher in male than female individuals (in 1990: 3.63 per 100,000 in male individuals, 2.88 in female individuals; and in 2019: 7.95 per 100,000 in male individuals, 6.31 in female individuals) globally (Figure 1A, Table 1). This phenomenon was found in five SDI levels, four WBI levels, and most geographical regions. However, in Australasia and high-income Asia Pacific in 1990 and 2019 and western Europe in 2019, the reverse applied. And both sexes shared a quite similar EAPC (3.04, 95% CI 2.87~3.21 in male and 3.06, 95% CI 2.75~3.38 in female individuals) (Figure 1D, **Table 5**). In general, most regions had a rising trend of ASIR from 1990 to 2019 in both genders (Figure 1D, **Table 5**). Male individuals in Australasia had the most rapid increase in ASIR (EAPC = 7.65, 95% CI 7.33~7.96) and female individuals in eastern Europe had the highest EAPC of 8.26 (95% CI 7.68~8.83). Nevertheless, female individuals in southern Sub-Saharan Africa showed the greatest decrease (EAPC: −0.5, 95% CI −0.56~−0.44).

### The prevalence and its trend

**Figure 2:**
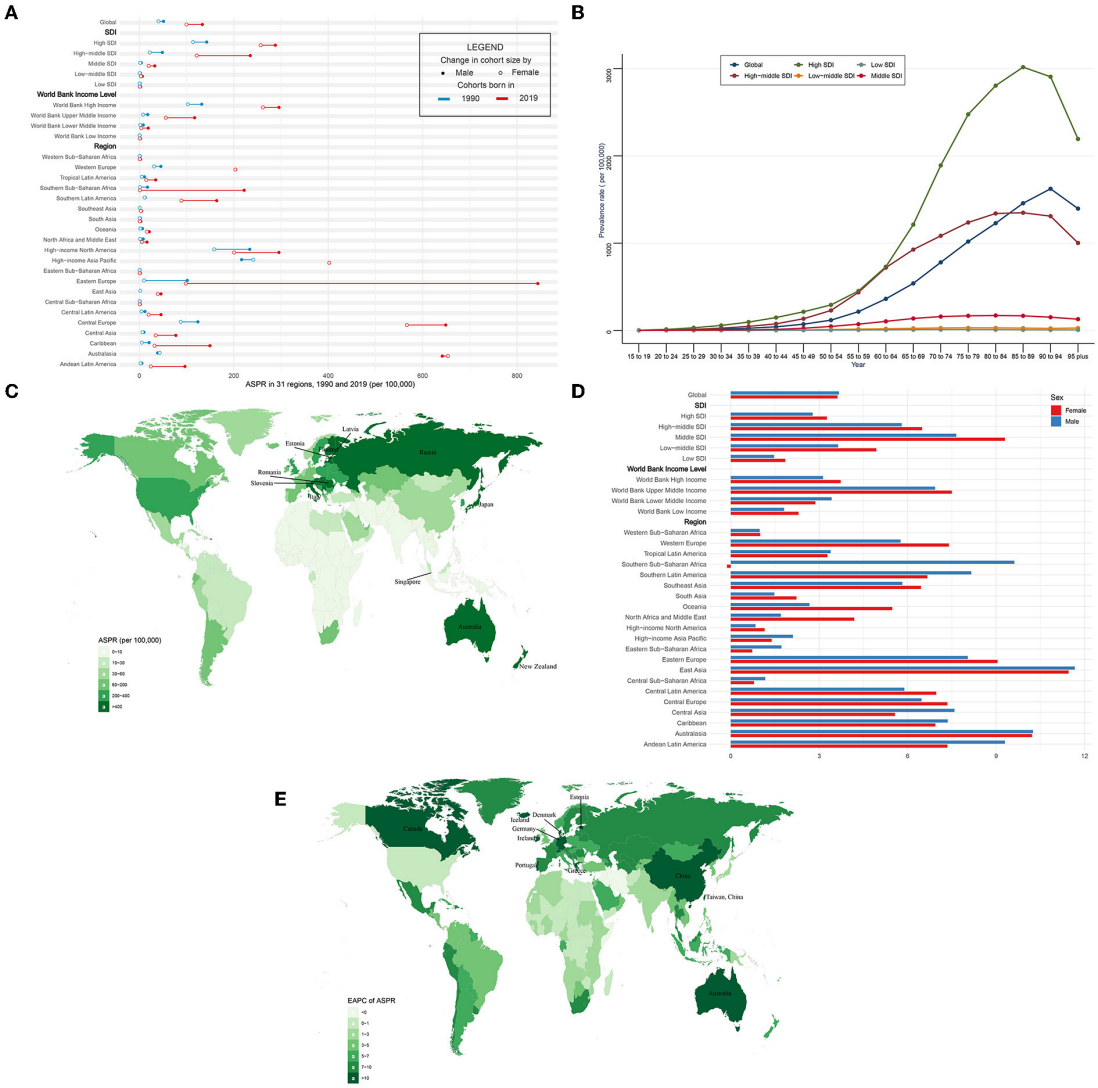
ASPR and its trend of CAVD. **(A)** ASPR in 31 regions from 1990 to 2019. **(B)** Prevalence rate stratified by age in the globe and 5 SDI regions in 2019. **(C)** ASPR in 204 countries and territories in 2019. **(D)** EAPC of ASPR in 31 regions from 1990 to 2019. **(E)** EAPC of ASPR in 204 countries and territories from 1990 to 2019. CAVD, calcific aortic valve disease; ASPR, the age-standardized prevalence rate; SDI, socio-demographic index; EAPC, estimated annual percentage change.

In the globe, the prevalence of CAVD cases remarkably increased by 443% from 1,732,988 in 1990 to 9,404,077 cases in 2019 (Table 2). At the same time, the age-standardized prevalence rate (ASPR) of CAVD increased by 120% from 45.54 (95% UI 37.61~54.67) per 100,000 in 1990 to 116.34 (95% UI 100.39~134.50) in 2019 (EAPC = 3.65, 95% CI 3.4~3.91) (Tables 2, **5**). Socio-demographic factor subgroup analysis indicated that high SDI regions showed the most prevalence cases (1,324,934 in 1990 and 5,095,444 in 2019) and highest ASPR (126.83 per 100,000 in 1990 and 273.52 in 2019) (Table 2). However, middle SDI regions showed the most rapid increase (EAPC = 8.19, 95% CI 7.9~8.49) (**Table 5**). Among WBI levels, the situation was analogous. As for geographical regions, high-income north America and high-income Asia Pacific were the top two regions with the highest ASPR in 1990 (191.35 and 233.42 per 100,000, respectively), but in 2019, they were central Europe and Australasia (ASPR: 608.31 and 649.50 per 100,000, respectively) (Table 2). Meanwhile, Australasia and East Asia had the fastest increase in ASPR (EAPC of Australasia: 10.18, 95% CI 9.7~10.67; EAPC of East Asia: 11.5, 95% CI 10.86~12.13) (**Table 5**). Among 204 countries and territories, the USA and Japan had the highest ASPR in 1990 (210.23 and 261.58 per 100,000, respectively), while Romania and Slovenia were the top two countries with the highest ASPR in 2019 (1,044.49 and 1,080.06 per 100,000, respectively) (Figure 2C; Supplementary Table 1). Furthermore, Germany and Iceland had the fastest rise in ASPR (EAPC = 15.15, 95% CI 12.8~17.56 and 15.28, 95% CI 12.56~18.06, respectively) (Figure 2E; Supplementary Table 2). Prevalence was positively correlated with age and peaked at age of 90 to 94 globally (Figure 2B).

**Table 2:** The prevalence of CAVD in 1990/2019.

	**1990**						**2019**					
	**Prevalent cases No** *10^**3**^ **(95% UI)**			**ASPR/100,000 (95% UI)**			**Prevalent cases No** *10^**3**^ **(95% UI)**			**ASPR/100,000 (95% UI)**		
	**Male**	**Female**	**Both**	**Male**	**Female**	**Both**	**Male**	**Female**	**Both**	**Male**	**Female**	**Both**
Global	894.5 (741.59~1067.5)	838.49 (690.26~1016.94)	1732.99 (1431.47~2074.81)	51.19 (42.68~60.91)	40.28 (32.99~48.82)	45.54 (37.61~54.67)	5027.26 (4276.88~5861.59)	4376.82 (3771.24~5082.8)	9404.08 (8079.6~10889.73)	133.38 (113.79~154.58)	99.86 (86.1~115.88)	116.34 (100.39~134.5)
SDI level												
High SDI	632.57 (523.39~760.43)	692.36 (563.23~842.26)	1324.93 (1090~1602.16)	142.6 (117.67~171.17)	113.91 (93.22~137.94)	126.83 (104.61~152.72)	2385.46 (2064.99~2748.14)	2709.98 (2327.88~3211.14)	5095.44 (4402.07~5933.38)	288.07 (250.19~330.79)	256.98 (221.02~300.21)	273.52 (237.08~315.22)
High-middle SDI	230.81 (188.4~277.51)	134.13 (110.52~160.59)	364.93 (300.69~436.29)	48.72 (39.87~58.5)	22.04 (18.22~26.36)	33.9 (27.98~40.44)	2185.22 (1810.72~2589.05)	1384.6 (1166.85~1627.03)	3569.82 (3002.41~4203.73)	234.86 (194.65~277.14)	121.54 (102.65~142.7)	174.53 (147.33~204.54)
Middle SDI	21.52 (16.92~26.64)	8.98 (7~11.44)	30.51 (24.09~37.76)	4 (3.16~4.93)	1.68 (1.31~2.13)	2.82 (2.23~3.49)	403.23 (326.37~489.15)	255.32 (203.74~311.73)	658.55 (529.93~800.78)	32.62 (26.5~39.34)	19.6 (15.69~23.94)	25.93 (20.92~31.47)
Low-middle SDI	7.05 (5.57~8.79)	2.31 (1.77~2.97)	9.36 (7.35~11.71)	2.12 (1.69~2.6)	0.76 (0.59~0.98)	1.45 (1.15~1.79)	43.85 (35.46~53.49)	23.41 (18.56~29.17)	67.26 (54.36~81.97)	6.34 (5.14~7.64)	3.3 (2.63~4.09)	4.78 (3.86~5.82)
Low SDI	2.34 (1.83~2.93)	0.59 (0.43~0.78)	2.93 (2.3~3.72)	1.69 (1.35~2.11)	0.52 (0.39~0.68)	1.12 (0.88~1.38)	7.43 (5.89~9.24)	2.24 (1.69~2.9)	9.68 (7.61~12.14)	2.5 (1.99~3.1)	0.86 (0.66~1.1)	1.67 (1.34~2.07)
World Bank Income Level												
World Bank High Income	720.96 (596.8~862.18)	765.97 (623.9~931.67)	1486.93 (1226.29~1790.77)	132.11 (109.85~158.01)	102.63 (84.23~123.72)	116.01 (95.82~139.03)	2913.58 (2525.2~3353.42)	3314.67 (2856.43~3900.43)	6228.25 (5389.24~7231.3)	295.76 (256.26~338.43)	261.86 (226.25~305.36)	279.68 (242.58~322.69)
World Bank Upper Middle Income	128.86 (103.46~157.41)	63.17 (51.17~76.63)	192.03 (155.18~232.96)	17.74 (14.32~21.54)	7.88 (6.4~9.57)	12.5 (10.17~15.09)	1889.48 (1556.27~2269.7)	1011.97 (832.19~1208.2)	2901.45 (2400.08~3460.03)	117.06 (96.4~140.16)	55.94 (46.06~66.6)	84.88 (70.35~101.1)
World Bank Lower Middle Income	43.31 (34.49~52.59)	8.83 (6.88~11.19)	52.14 (41.82~63.1)	8.29 (6.68~10.07)	1.84 (1.44~2.33)	5.01 (4.02~6.04)	218.05 (174.89~264.45)	47.18 (37.93~57.83)	265.23 (214.14~320.12)	18.79 (15.21~22.79)	4.02 (3.24~4.89)	11.11 (9.01~13.35)
World Bank Low Income	1.16 (0.91~1.45)	0.4 (0.3~0.54)	1.56 (1.22~1.97)	1.4 (1.1~1.73)	0.54 (0.41~0.71)	0.96 (0.76~1.18)	4.08 (3.22~5.04)	1.7 (1.3~2.17)	5.77 (4.54~7.17)	2.34 (1.85~2.88)	1.04 (0.81~1.33)	1.67 (1.32~2.06)
Region												
Andean Latin America	0.56 (0.45~0.69)	0.27 (0.21~0.35)	0.83 (0.66~1.03)	5.25 (4.22~6.41)	2.37 (1.82~3.03)	3.77 (3.02~4.63)	26.25 (22.25~30.53)	7.1 (5.81~8.45)	33.35 (28.25~39.02)	96.53 (81.91~112.71)	24.29 (19.85~28.95)	59.04 (50.07~69.05)
Australasia	4.03 (3.23~5.03)	5.85 (4.83~7.03)	9.88 (8.18~11.83)	38.54 (30.95~47.68)	42.99 (35.39~51.62)	41.77 (34.78~49.88)	145.81 (124.04~176.73)	175.01 (146.24~209.39)	320.82 (272.23~381.06)	641.53 (548.23~771.77)	653.5 (546.89~785.16)	649.5 (552~772.74)
Caribbean	2.64 (2.18~3.2)	0.72 (0.57~0.89)	3.36 (2.75~4.01)	20.63 (17.03~25.03)	5.2 (4.14~6.44)	12.61 (10.3~15.1)	36.64 (30.14~44.78)	8.83 (7.18~10.64)	45.47 (37.69~54.85)	149.46 (123.31~182.28)	32.16 (26.19~38.74)	87.51 (72.62~105.56)
Central Asia	1.91 (1.46~2.38)	1.86 (1.44~2.35)	3.77 (2.96~4.71)	9.8 (7.66~12.16)	6.69 (5.22~8.43)	7.92 (6.22~9.83)	26.75 (21.47~32.02)	14.31 (11.58~17.08)	41.06 (33.49~48.81)	77.22 (62.76~91.41)	34.51 (28.22~40.92)	53.22 (43.89~62.92)
Central Europe	79.88 (66.06~95.09)	74.78 (61.25~89.11)	154.66 (128.34~184.57)	123.97 (102.96~146.99)	87.57 (71.89~104.38)	104.18 (86.62~123.47)	582.72 (493.97~689.09)	677.83 (575.85~802.85)	1260.56 (1067.65~1479.62)	648.83 (551.63~763.85)	566.62 (482.05~673.31)	608.31 (517.94~713.51)
Central Latin America	4.96 (3.98~6)	2.04 (1.61~2.53)	7 (5.59~8.53)	11.78 (9.52~14.38)	4.45 (3.51~5.52)	7.98 (6.44~9.71)	51.88 (43.38~61.48)	25.08 (20.49~30.42)	76.96 (63.86~91.38)	45.83 (38.37~54.3)	19.54 (15.96~23.74)	31.82 (26.45~37.75)
Central Sub-Saharan Africa	0.18 (0.14~0.22)	0.07 (0.05~0.09)	0.25 (0.19~0.32)	1.42 (1.12~1.79)	0.62 (0.46~0.82)	1 (0.79~1.26)	0.58 (0.45~0.75)	0.21 (0.16~0.28)	0.8 (0.62~1.01)	2.03 (1.6~2.54)	0.78 (0.6~0.99)	1.36 (1.08~1.69)
East Asia	9.79 (7.21~13.03)	8.48 (6.28~11.34)	18.27 (13.68~24.32)	2.19 (1.63~2.88)	1.94 (1.45~2.54)	2.1 (1.57~2.74)	466.89 (370.91~566.03)	424.13 (335.82~519.55)	891.02 (707.25~1093.31)	45.83 (36.68~55.4)	39 (30.97~47.63)	42.41 (33.87~51.68)
Eastern Europe	103.55 (81.71~128.12)	18.11 (14.27~22.87)	121.66 (97.12~148.88)	101.46 (81.16~123.89)	10.01 (7.96~12.57)	43.32 (34.79~52.76)	1114.74 (894.48~1351.95)	213.94 (170.2~259.17)	1328.69 (1065.7~1605.21)	843.96 (678.61~1017.4)	98.37 (78.91~118.49)	395.8 (319.64~477)
Eastern Sub-Saharan Africa	0.56 (0.44~0.71)	0.2 (0.15~0.27)	0.76 (0.59~0.96)	1.29 (1.02~1.61)	0.56 (0.43~0.73)	0.93 (0.73~1.15)	2.03 (1.55~2.57)	0.57 (0.43~0.76)	2.6 (2~3.27)	2.15 (1.69~2.68)	0.7 (0.53~0.91)	1.4 (1.1~1.74)
High-income Asia Pacific	195.57 (159.86~238.65)	273.98 (222.49~332.08)	469.56 (383.48~566.44)	216.47 (177.61~261.2)	241.28 (196.5~291.56)	233.42 (191.41~280.25)	709.94 (604.2~831.52)	1005.76 (841.19~1215.47)	1715.7 (1450.88~2042.13)	401.97 (341.36~468.6)	402.2 (338.33~481.05)	408.4 (348.93~479.89)
High-income North America	352.7 (287.81~429.14)	334.97 (268.61~413.09)	687.66 (557.97~840.92)	233.74 (190.72~283.24)	158.45 (128.41~193.96)	191.35 (155.87~232.93)	807.35 (704.42~936.66)	685.54 (595.4~793.05)	1492.89 (1305.2~1727.5)	295.58 (258.9~338.66)	200.55 (175.67~230.07)	244.39 (214.46~279.6)
North Africa and Middle East	8.31 (6.49~10.17)	1.38 (1.03~1.79)	9.7 (7.64~11.9)	8.19 (6.48~10.01)	1.51 (1.15~1.96)	4.92 (3.9~6.04)	42.78 (33.73~52.9)	11.52 (8.98~14.41)	54.3 (43.23~66.78)	16.12 (12.82~19.87)	5.15 (4.08~6.4)	10.83 (8.67~13.35)
Oceania	0.09 (0.07~0.11)	0.02 (0.02~0.03)	0.11 (0.09~0.14)	6.54 (5.25~8.28)	1.99 (1.6~2.5)	4.3 (3.49~5.36)	0.68 (0.54~0.83)	0.45 (0.36~0.56)	1.13 (0.9~1.37)	21.01 (17.04~25.69)	16.03 (12.75~19.73)	18.61 (14.95~22.75)
South Asia	6.93 (5.36~8.8)	1.4 (0.99~1.95)	8.33 (6.4~10.64)	2.07 (1.63~2.6)	0.53 (0.39~0.71)	1.34 (1.04~1.67)	23.36 (18.46~29.12)	6.83 (5.06~9.18)	30.19 (23.68~37.63)	3.1 (2.46~3.85)	0.96 (0.72~1.28)	2.03 (1.6~2.52)
Southeast Asia	1.08 (0.8~1.41)	0.71 (0.51~0.96)	1.78 (1.34~2.36)	0.86 (0.65~1.12)	0.55 (0.4~0.72)	0.7 (0.53~0.91)	13.23 (10.27~16.94)	10.75 (8.36~13.78)	23.99 (18.74~30.27)	4.73 (3.76~5.95)	3.43 (2.7~4.37)	4.06 (3.21~5.09)
Southern Latin America	2.62 (2.1~3.23)	2.87 (2.24~3.84)	5.5 (4.39~6.94)	12.4 (9.98~15.27)	11.14 (8.73~14.92)	11.87 (9.53~14.91)	59.7 (50.59~71.86)	42.19 (35.18~50.8)	101.89 (87.12~120.8)	163.8 (139.01~196.84)	89.01 (74.38~106.77)	122.72 (104.78~145.67)
Southern Sub-Saharan Africa	2.18 (1.69~2.73)	0.18 (0.14~0.23)	2.37 (1.84~2.94)	17.15 (13.32~21.26)	1.17 (0.91~1.5)	8.15 (6.36~10.13)	57.02 (43.85~72.95)	0.39 (0.3~0.51)	57.41 (44.28~73.39)	221.92 (171.05~280.83)	1.23 (0.95~1.58)	94.86 (72.97~120.7)
Tropical Latin America	5.29 (4.23~6.47)	2.74 (2.18~3.4)	8.03 (6.43~9.81)	10.75 (8.57~13.09)	5.13 (4.09~6.33)	7.79 (6.23~9.54)	39.09 (31.08~48.19)	19.51 (15.59~24.04)	58.6 (47.23~72.05)	34.55 (27.66~42.42)	14.62 (11.68~17.97)	23.73 (19.16~29.01)
Western Europe	110.62 (88.68~135.53)	107.59 (86.28~133.7)	218.21 (177.74~268.12)	45.56 (36.74~55.61)	30.99 (25~38.25)	37.65 (30.75~45.97)	816.75 (693.15~958.79)	1046.03 (883.03~1250.05)	1862.79 (1577.56~2209.09)	204.08 (172.96~239.05)	203.21 (170.91~241.76)	204.84 (174.62~240.59)
Western Sub-Saharan Africa	1.04 (0.79~1.33)	0.26 (0.19~0.35)	1.3 (1~1.63)	1.82 (1.4~2.31)	0.61 (0.45~0.83)	1.27 (0.99~1.58)	3.06 (2.35~3.91)	0.81 (0.6~1.09)	3.87 (3~4.88)	2.53 (1.95~3.21)	0.84 (0.63~1.12)	1.65 (1.3~2.04)

CAVD, calcific aortic valve disease; ASPR, the age-standardized prevalence rate; SDI, socio-demographic index.

The ASPR was higher in male than female individuals (in 1990: 51.19 per 100,000 in male individuals, 40.28 in female individuals, male to female ratio = 1.27; in 2019: 133.38 per 100,000 in male individuals, 99.86 in female individuals, ratio = 1.34) globally (Figure 2A, Table 2). Male and female individuals showed a similar growing trend of ASPR during the past 30 years (EAPC of males: 3.67, 95% CI 3.46~3.88; EAPC of females: 3.62, 95% CI 3.31~3.93) (Figure 2D, **Table 5**). Among five SDI levels, four WBI levels, and 21 GBD regions, there were slight differences in the variation tendency of ASPR in this period (Figures 2A,D, **Table 5**). However, female individuals in southern sub-Saharan Africa showed a downward trend (EAPC = −0.13, 95% CI −0.27~0) of ASPR while male individuals in this region showed a relatively huge increase (EAPC = 9.62, 95% CI 8.13~11.14).

### The death and its trend

**Figure 3:**
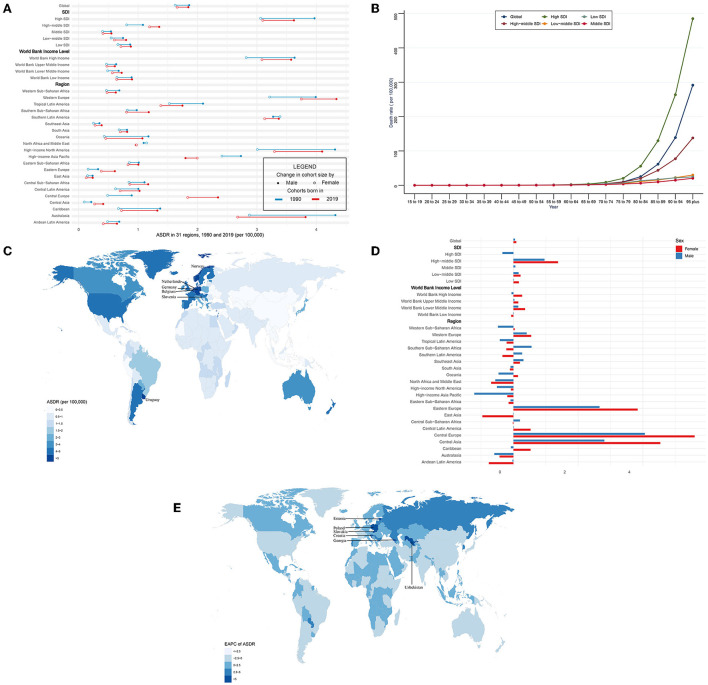
ASDR and its trend of CAVD. **(A)** ASDR in 31 regions from 1990 to 2019. **(B)** Death rate stratified by age in the globe and 5 SDI regions in 2019. **(C)** ASDR in 204 countries and territories in 2019. **(D)** EAPC of ASDR in 31 regions from 1990 to 2019. **(E)** EAPC of ASDR in 204 countries and territories from 1990 to 2019. CAVD, calcific aortic valve disease; ASDR, the age-standardized death rate; SDI, socio-demographic index; EAPC, estimated annual percentage change.

In general, CAVD-related death cases significantly increased from 53,298 in 1990 to 126,827 in 2019 while the age-standardized death rate (ASDR) was relatively stable during the past 30 years (from 1.75 per 100,000 in 1990 to 1.76 in 2019, EAPC = 0.06, 95% CI −0.04~0.15) (Tables 3, **5**). In the SDI level, the high SDI regions had the highest burden until 2019 [death cases: 36,015 in 1990 and 80,211 in 2019, ASDR: 3.46 (95% UI 3.06~3.84) per 100,000 in 1990 and 3.35 (95% UI 2.75~3.74) in 2019] (Table 3). At the same time, high-middle SDI regions experienced the most rapid increase in this period (ASDR: 0.93 in 1990 and 1.28 in 2019, EAPC = 1.17, 95% CI 0.89~1.45) (**Table 5**). Similar results were found at the WBI level. Subgroup analysis by geographical regions showed that western Europe and high-income north America were the top two regions with the highest ASDR (western Europe: 3.59 per 100,000 in 1990 and 4.05 in 2019; high-income north America: 3.56 per 100,000 in 1990 and 3.64 in 2019). Central Europe had the fastest rise in ASDR (EAPC = 4.85, 95% CI 4.43~5.27), whereas North Africa and the middle east (EAPC = −0.65, 95% CI −0.7~−0.61) and east Asia (EAPC = −0.54, 95% CI −0.86~−0.21) showed the fastest decrease in ASDR. Among 204 countries and territories, the highest ASDR was in Cyprus (10.21 per 100,000) in 1990, followed by Norway (5.55 per 100,000) and Bermuda (5.29 per 100,000) (Figure 3C; Supplementary Table 1). Meanwhile, the hugest annual increase of ASDR during the past 30 years was in Poland (EAPC = 9.44, 95% CI 8.09~10.8), followed by Czechia (EAPC = 8.72, 95% CI 7.78~9.68) and Estonia (EAPC = 8.6, 95% CI 7.35~9.87), while the largest annual decrease of ASDR was in Qatar (EAPC = −3.24, 95% CI −3.6~−2.88), followed by Syrian (EAPC = −3.01, 95% CI −3.53~−2.49) and Panama (EAPC = −2.99, 95% CI −3.44~−2.53) (Figure 3E; Supplementary Table 2). It was indicated that the ASDR of CAVD was positively related to age, especially for those aged 80 years and over. And the fastest elevation of the ASDR was found among elderly patients in high SDI regions (Figure 3B).

**Table 3:** The death of CAVD in 1990/2019.

	**1990**						**2019**					
	Death cases No*10^**3**^ **(95% UI)**			**ASDR/100,000 (95% UI)**			**Death cases No** *10^**3**^ **(95% UI)**			**ASDR/100,000 (95% UI)**		
	**Male**	**Female**	**Both**	**Male**	**Female**	**Both**	**Male**	**Female**	**Both**	**Male**	**Female**	**Both**
Global	24.52 (22.44~27.03)	28.78 (24.82~34.07)	53.3 (47.76~59.73)	1.86 (1.69~2.04)	1.62 (1.39~1.91)	1.75 (1.55~1.96)	54.17 (47.77~58.67)	72.65 (57.76~84.3)	126.83 (105.6~141.39)	1.85 (1.58~2.01)	1.66 (1.32~1.92)	1.76 (1.45~1.97)
SDI level												
High SDI	15.18 (14.09~16.49)	20.84 (17.97~24.02)	36.02 (32.13~39.86)	3.97 (3.65~4.31)	3.06 (2.63~3.54)	3.46 (3.06~3.84)	31.77 (27.2~34.65)	48.45 (37.05~56.49)	80.21 (64.3~90.1)	3.62 (3.11~3.96)	3.1 (2.45~3.59)	3.35 (2.75~3.74)
High-middle SDI	4.07 (3.66~4.44)	4.29 (3.58~5.08)	8.36 (7.42~9.21)	1.08 (0.95~1.2)	0.81 (0.67~0.95)	0.93 (0.81~1.04)	10.12 (9.02~11.1)	14.31 (11.66~16.41)	24.44 (20.86~27.26)	1.36 (1.18~1.49)	1.19 (0.97~1.37)	1.28 (1.08~1.43)
Middle SDI	2.44 (2.07~2.86)	1.83 (1.34~2.49)	4.27 (3.5~5.17)	0.54 (0.46~0.63)	0.4 (0.3~0.53)	0.47 (0.39~0.57)	5.73 (5.11~6.56)	4.75 (4.03~5.46)	10.48 (9.43~11.74)	0.55 (0.49~0.63)	0.41 (0.35~0.47)	0.48 (0.43~0.53)
Low-middle SDI	1.89 (1.32~2.68)	1.23 (0.65~2.25)	3.12 (2.1~4.2)	0.75 (0.53~1.04)	0.55 (0.3~0.93)	0.65 (0.44~0.85)	4.53 (3.69~5.76)	3.63 (2.61~4.99)	8.17 (6.63~9.99)	0.8 (0.64~1)	0.6 (0.43~0.8)	0.7 (0.57~0.84)
Low SDI	0.93 (0.56~1.4)	0.58 (0.27~1.19)	1.5 (0.88~2.2)	0.87 (0.57~1.27)	0.66 (0.33~1.25)	0.77 (0.47~1.09)	1.99 (1.48~2.64)	1.48 (0.99~2.27)	3.46 (2.56~4.39)	0.88 (0.65~1.14)	0.71 (0.48~1.02)	0.8 (0.61~0.99)
World Bank Income Level												
World Bank High Income	17.17 (15.95~18.55)	23.15 (19.88~26.73)	40.33 (36.18~44.65)	3.63 (3.34~3.93)	2.82 (2.42~3.27)	3.19 (2.84~3.53)	37.59 (32.4~40.92)	57.87 (44.87~67.4)	95.46 (76.94~107.1)	3.58 (3.09~3.9)	3.08 (2.43~3.58)	3.33 (2.73~3.7)
World Bank Upper Middle Income	3.82 (3.39~4.3)	3.17 (2.63~3.84)	6.99 (6.13~7.92)	0.63 (0.56~0.7)	0.47 (0.39~0.55)	0.55 (0.48~0.62)	8.27 (7.44~9.26)	8.04 (6.95~9.14)	16.31 (14.61~18.15)	0.61 (0.54~0.68)	0.47 (0.41~0.54)	0.54 (0.48~0.6)
World Bank Lower Middle Income	2.93 (2.04~4.14)	2.05 (1.06~3.65)	4.98 (3.31~6.73)	0.67 (0.48~0.93)	0.49 (0.26~0.83)	0.58 (0.39~0.77)	7.1 (5.67~8.94)	5.83 (4.22~7.7)	12.92 (10.65~15.75)	0.72 (0.57~0.91)	0.57 (0.41~0.74)	0.65 (0.53~0.78)
World Bank Low Income	0.58 (0.37~0.87)	0.39 (0.22~0.77)	0.97 (0.62~1.41)	0.89 (0.6~1.29)	0.64 (0.38~1.16)	0.77 (0.51~1.09)	1.19 (0.88~1.6)	0.87 (0.61~1.32)	2.06 (1.57~2.71)	0.9 (0.66~1.19)	0.64 (0.46~0.91)	0.77 (0.6~0.97)
Region												
Andean Latin America	0.07 (0.05~0.09)	0.05 (0.03~0.07)	0.12 (0.09~0.15)	0.68 (0.52~0.87)	0.49 (0.33~0.64)	0.58 (0.45~0.71)	0.17 (0.13~0.21)	0.12 (0.1~0.15)	0.29 (0.24~0.35)	0.64 (0.5~0.81)	0.41 (0.33~0.52)	0.53 (0.43~0.64)
Australasia	0.38 (0.34~0.41)	0.39 (0.33~0.46)	0.77 (0.69~0.85)	4.32 (3.89~4.68)	2.87 (2.41~3.35)	3.48 (3.07~3.85)	0.9 (0.77~1.02)	0.96 (0.74~1.14)	1.87 (1.51~2.11)	3.82 (3.24~4.3)	2.68 (2.1~3.17)	3.18 (2.62~3.58)
Caribbean	0.17 (0.14~0.19)	0.09 (0.07~0.11)	0.25 (0.22~0.28)	1.37 (1.2~1.54)	0.67 (0.54~0.8)	1 (0.87~1.12)	0.31 (0.26~0.37)	0.2 (0.16~0.27)	0.52 (0.42~0.63)	1.32 (1.08~1.58)	0.72 (0.57~0.95)	1 (0.82~1.22)
Central Asia	0.04 (0.03~0.04)	0.03 (0.02~0.03)	0.06 (0.05~0.07)	0.21 (0.17~0.25)	0.09 (0.07~0.11)	0.14 (0.11~0.16)	0.11 (0.09~0.15)	0.09 (0.08~0.13)	0.21 (0.17~0.26)	0.41 (0.35~0.52)	0.27 (0.22~0.37)	0.33 (0.27~0.43)
Central Europe	0.51 (0.43~0.64)	0.4 (0.29~0.52)	0.9 (0.75~1.11)	0.89 (0.76~1.15)	0.49 (0.35~0.64)	0.66 (0.54~0.81)	2.01 (1.56~2.36)	2.66 (2.05~3.28)	4.68 (3.77~5.58)	2.35 (1.81~2.76)	1.83 (1.41~2.26)	2.08 (1.68~2.49)
Central Latin America	0.41 (0.37~0.44)	0.25 (0.21~0.31)	0.66 (0.6~0.73)	1 (0.9~1.1)	0.62 (0.51~0.75)	0.81 (0.74~0.9)	1.09 (0.87~1.35)	0.87 (0.7~1.14)	1.96 (1.61~2.43)	1.02 (0.82~1.27)	0.7 (0.56~0.92)	0.85 (0.7~1.06)
Central Sub-Saharan Africa	0.1 (0.06~0.18)	0.07 (0.04~0.14)	0.17 (0.1~0.26)	1.11 (0.74~1.86)	0.85 (0.48~1.56)	0.97 (0.63~1.42)	0.22 (0.15~0.35)	0.19 (0.13~0.29)	0.41 (0.29~0.58)	1.17 (0.85~1.72)	0.86 (0.58~1.29)	1.01 (0.74~1.36)
East Asia	0.95 (0.65~1.27)	0.62 (0.33~1.05)	1.57 (1.02~2.22)	0.23 (0.17~0.31)	0.15 (0.08~0.25)	0.19 (0.12~0.27)	1.95 (1.53~2.5)	1.28 (0.96~1.6)	3.23 (2.62~3.85)	0.24 (0.19~0.29)	0.13 (0.1~0.16)	0.17 (0.14~0.2)
Eastern Europe	0.28 (0.22~0.32)	0.28 (0.2~0.33)	0.56 (0.44~0.63)	0.33 (0.27~0.37)	0.16 (0.11~0.19)	0.21 (0.17~0.24)	0.75 (0.6~0.9)	0.89 (0.72~1.09)	1.64 (1.37~1.93)	0.61 (0.49~0.72)	0.38 (0.31~0.47)	0.48 (0.4~0.57)
Eastern Sub-Saharan Africa	0.33 (0.21~0.48)	0.24 (0.14~0.47)	0.57 (0.38~0.85)	1.01 (0.69~1.38)	0.85 (0.53~1.48)	0.94 (0.66~1.34)	0.66 (0.5~0.91)	0.53 (0.38~0.75)	1.2 (0.95~1.53)	1 (0.71~1.42)	0.83 (0.58~1.15)	0.92 (0.72~1.21)
High-income Asia Pacific	1.79 (1.62~2.03)	2.49 (2.02~3.12)	4.28 (3.66~5.07)	2.73 (2.44~3.12)	2.41 (1.94~3.02)	2.57 (2.15~3.06)	4.01 (3.11~4.61)	9.56 (6.01~11.97)	13.57 (9.08~16.51)	1.8 (1.4~2.06)	2 (1.32~2.45)	1.98 (1.4~2.36)
High-income North America	5.88 (5.41~6.33)	7.28 (6.13~8.22)	13.16 (11.62~14.39)	4.31 (3.96~4.63)	3.01 (2.54~3.4)	3.56 (3.14~3.89)	11.57 (9.95~12.98)	15.62 (12.29~17.91)	27.19 (22.16~30.24)	4.1 (3.53~4.59)	3.3 (2.67~3.77)	3.64 (3.02~4.02)
North Africa and Middle East	0.9 (0.65~1.2)	0.83 (0.51~1.18)	1.73 (1.26~2.18)	1.09 (0.79~1.44)	1.14 (0.69~1.59)	1.13 (0.82~1.45)	2.01 (1.56~2.41)	1.81 (1.4~2.18)	3.82 (3.19~4.44)	0.96 (0.75~1.14)	0.97 (0.74~1.17)	0.97 (0.81~1.12)
Oceania	0.013 (0.008~0.02)	0.005 (0.003~0.009)	0.02 (0.01~0.03)	1.17 (0.76~1.77)	0.43 (0.24~0.75)	0.79 (0.52~1.12)	0.03 (0.02~0.04)	0.012 (0.007~0.02)	0.04 (0.03~0.06)	1.07 (0.76~1.54)	0.46 (0.3~0.74)	0.75 (0.54~1.04)
South Asia	1.87 (1.2~2.87)	1.19 (0.48~2.41)	3.06 (1.85~4.37)	0.81 (0.53~1.22)	0.68 (0.29~1.24)	0.75 (0.47~1.03)	4.73 (3.62~6.47)	4.02 (2.65~5.67)	8.75 (6.85~11.05)	0.82 (0.62~1.09)	0.7 (0.46~0.96)	0.76 (0.6~0.95)
Southeast Asia	0.37 (0.29~0.53)	0.29 (0.21~0.39)	0.66 (0.54~0.86)	0.35 (0.27~0.51)	0.25 (0.18~0.33)	0.3 (0.24~0.38)	0.93 (0.71~1.31)	0.78 (0.6~0.93)	1.7 (1.4~2.13)	0.39 (0.3~0.54)	0.28 (0.21~0.33)	0.33 (0.27~0.41)
Southern Latin America	0.59 (0.52~0.67)	0.8 (0.68~0.92)	1.39 (1.22~1.56)	3.27 (2.9~3.71)	3.39 (2.86~3.91)	3.43 (3.01~3.85)	1.15 (1.03~1.26)	1.69 (1.41~1.95)	2.84 (2.46~3.16)	3.37 (2.99~3.7)	3.13 (2.65~3.59)	3.3 (2.85~3.66)
Southern Sub-Saharan Africa	0.11 (0.09~0.13)	0.11 (0.09~0.14)	0.22 (0.19~0.25)	0.98 (0.82~1.18)	0.82 (0.68~0.98)	0.9 (0.78~1.04)	0.21 (0.18~0.24)	0.22 (0.18~0.25)	0.43 (0.37~0.48)	1.18 (0.95~1.34)	0.8 (0.66~0.92)	0.96 (0.82~1.07)
Tropical Latin America	0.89 (0.79~0.95)	0.65 (0.52~0.75)	1.53 (1.35~1.66)	2.09 (1.86~2.24)	1.53 (1.24~1.72)	1.81 (1.59~1.95)	1.76 (1.55~1.98)	1.82 (1.52~2.23)	3.58 (3.11~4.14)	1.75 (1.53~1.97)	1.38 (1.15~1.69)	1.56 (1.34~1.8)
Western Europe	8.56 (7.94~9.24)	12.54 (10.81~14.54)	21.1 (18.96~23.33)	3.99 (3.67~4.29)	3.21 (2.77~3.72)	3.59 (3.21~3.97)	18.95 (16.57~20.69)	28.94 (22.91~33.63)	47.89 (39.52~53.66)	4.34 (3.79~4.73)	3.75 (3.04~4.32)	4.05 (3.4~4.51)
Western Sub-Saharan Africa	0.34 (0.17~0.54)	0.18 (0.08~0.39)	0.51 (0.27~0.81)	0.68 (0.35~1.09)	0.47 (0.23~0.99)	0.6 (0.33~0.95)	0.64 (0.44~0.92)	0.38 (0.24~0.65)	1.02 (0.71~1.42)	0.63 (0.44~0.9)	0.48 (0.31~0.76)	0.55 (0.39~0.76)

CAVD, calcific aortic valve disease; ASDR, the age-standardized death rate; SDI, socio-demographic index.

Globally, CAVD-related death cases in female individuals were higher than those in male individuals (1990: 24,517 female and 28,780 male; 2019: 72,652 female and 54,175 male) (Table 3). But ASDR of CAVD in male individuals was higher than that in female individuals (1990: 1.86 per 100,000 in male and 1.62 in female individuals; 2019: 1.85 per 100,000 in male and 1.66 in female individuals) (Figure 3A, Table 3). And both sexes shared a quite low EAPC (0.05, 95% CI −0.06~0.16 in male and 0.09, 95% CI −0.03~0.21 in female individuals) (Figure 3D; **Table 5**). Subgroup analysis revealed that except for north Africa and the Middle East, high-income Asia Pacific in 2019, and southern Latin America in 1990, the CAVD-related death cases in female individuals were higher than those in male individuals (Table 3). The variation tendencies of ASDR in both genders were identical in most regions except western Sub-Saharan Africa, Southern Sub-Saharan Africa, Southern Latin America, Oceania, Central Sub-Saharan Africa, and the Caribbean.

### The DALY and its trend

**Figure 4:**
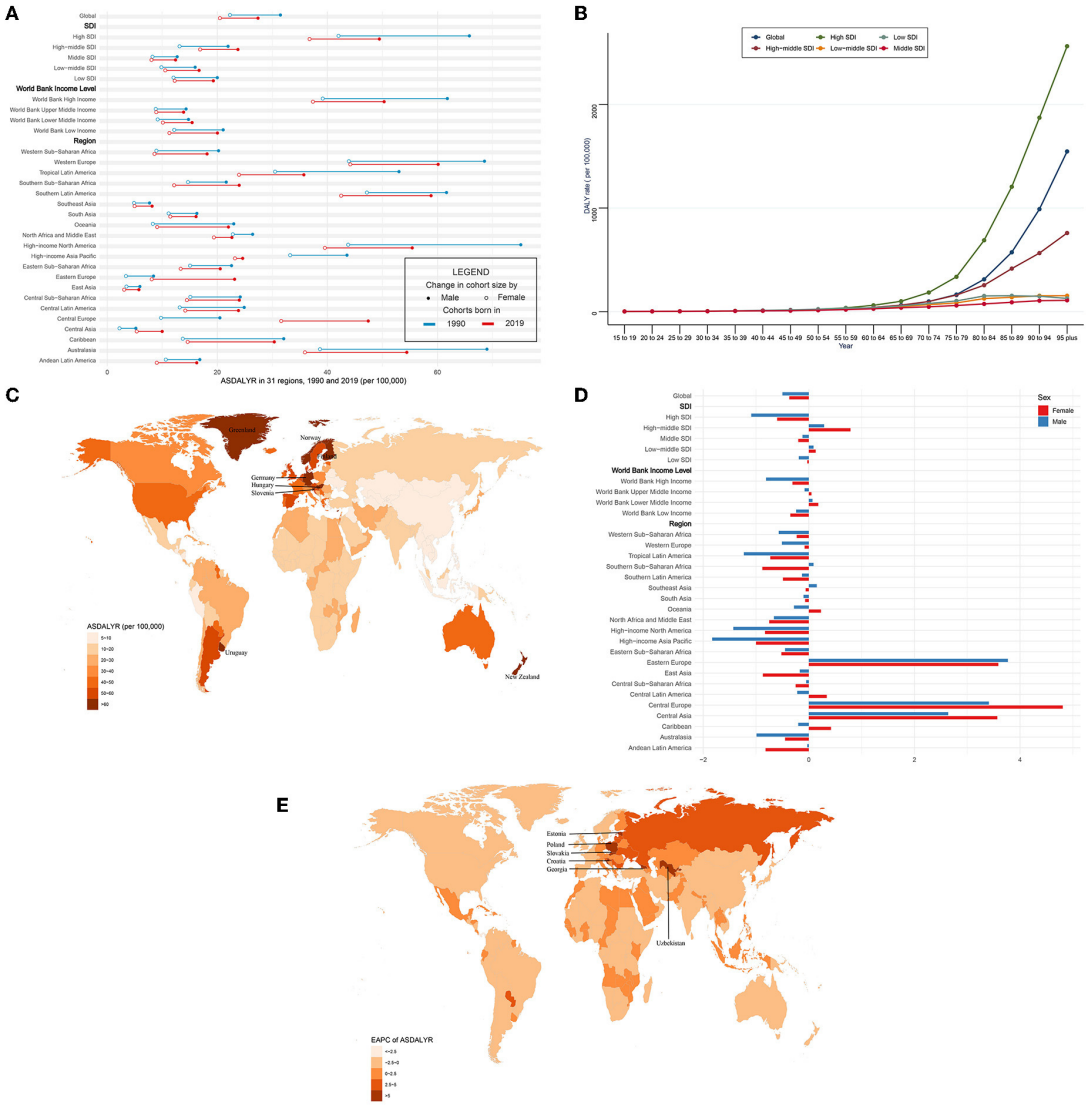
ASDALYR and its trend of CAVD. **(A)** ASDALYR in 31 regions from 1990 to 2019. **(B)** DALY rate stratified by age in the globe and 5 SDI regions in 2019. **(C)** ASDALYR in 204 countries and territories in 2019. **(D)** EAPC of ASDALYR in 31 regions from 1990 to 2019. **(E)** EAPC of ASDALYR in 204 countries and territories from 1990 to 2019. CAVD, calcific aortic valve disease; ASDALYR, the age-standardized disability-adjusted life years rate; SDI, socio-demographic index; EAPC, estimated annual percentage change.

Globally, the DALYs of CAVD increased by 88.3% from 975,894 in 1990 to 1,837,751 in 2019. On the contrary, the age-standardized DALY rate (ASDALYR) gradually declined from 26.8 per 100,000 to 1990 to 23.9 in 2019 (EAPC = −0.45, 95% CI −0.52~−0.38) (Tables 4, 5). Subgroup analysis by socio-demographic factors showed that the high SDI regions had the highest DALY until 2019 (553,755 in 1990 and 913,672 in 2019), while the ASDALYR had the largest decrease (EAPC = −0.84, 95% CI −1.01~−0.67) (Tables 4, 5). However, the ASDALYR in high-middle SDI regions had the fastest rise (from 17.3 per 100,000 in 1990 to 20.2 in 2019, EAPC = 0.51, 95% CI 0.36~0.67). Among WBI levels, the situation was similar. Subgroup analysis by geographical regions demonstrated that high-income North America, Western Europe, and Southern Latin America were the top three regions with the highest ASDALYR (high-income North America: 57.8 per 100,000 in 1990 and 46.8 in 2019; Western Europe: 55.5 in 1990 and 51.9 in 2019; Southern Latin America: 54.8 in 1990 and 50.5 in 2019) (Table 4). Meanwhile, Central Europe had the most rapid increase of ASDALYR (EAPC = 4.02, 95% CI 3.68~4.36), whereas high-income Asia Pacific showed the largest decline (EAPC = −1.32, 95% CI −1.74~−0.9) (Table 5). Among 204 countries and territories, the highest ASDALYR was found in Cyprus (131.6 per 100,000) in 1990, followed by Bermuda (111.0 per 100,000), and it was in Cyprus (96.1 per 100,000) in 2019, followed by Slovenia (91.6 per 100,000) (Figure 4C; Supplementary Table 1). In the meantime, Poland showed the most rapid increase in ASDALYR during this period (EAPC = 7.77, 95% CI 6.57~8.98), followed by Estonia (EAPC = 7.69, 95% CI 6.62~8.77) (Figure 4E; Supplementary Table 2). In contrast, Syria showed the fastest decrease in ASDALYR (EAPC = −3.15, 95% CI −3.69~−2.61). Moreover, the ASDALYR of CAVD was closely correlated to age globally and among five SDI regions. In detail, it slightly increased in patients under 75 years, while rapidly growing among patients older than 75 years. And the fastest elevation of the ASDALYR was in high SDI regions (Figure 4B).

**Table 4:** The DALYs of CAVD in 1990/2019.

	**1990**						**2019**					
	**DALYs No** *10^**3**^ **(95% UI)**			**ASDALYR/100,000 (95% UI)**			**DALYs No** *10^**3**^ **(95% UI)**			**ASDALYR/100,000 (95% UI)**		
	**Male**	**Female**	**Both**	**Male**	**Female**	**Both**	**Male**	**Female**	**Both**	**Male**	**Female**	**Both**
Global	533.66 (481.12~594.23)	442.23 (381.94~551.81)	975.89 (872.01~1109.79)	31.47 (28.7~34.63)	22.3 (19.27~27.31)	26.85 (24.07~30.31)	944.03 (864.57~1036.41)	893.72 (765.94~1032.79)	1837.75 (1637.02~2031.85)	27.4 (24.8~29.99)	20.48 (17.55~23.69)	23.9 (21.1~26.55)
SDI level												
High SDI	277.94 (259.85~300.65)	275.81 (241.47~320.12)	553.75 (508.56~609.84)	65.8 (61.39~71.14)	42.02 (36.76~48.87)	52.88 (48.59~58.27)	422.51 (380.72~457.52)	491.17 (404.32~565.07)	913.67 (778.95~1005.2)	49.45 (44.6~53.46)	36.76 (31.09~42.28)	42.88 (37.5~47)
High-middle SDI	101.8 (92.76~111.78)	76.25 (66.02~91.74)	178.05 (162.77~197.28)	21.96 (19.86~24.05)	13.14 (11.29~15.78)	17.25 (15.73~19.19)	200.7 (180.53~223.1)	194.95 (167.8~222.29)	395.65 (353.62~437.85)	23.75 (21.32~26.45)	16.88 (14.62~19.27)	20.21 (18.01~22.35)
Middle SDI	74.12 (62.61~87.44)	46.88 (34.06~66.91)	121 (98.91~146.12)	12.72 (10.78~14.89)	8.22 (6.01~11.42)	10.48 (8.62~12.68)	149.62 (132.77~171.97)	101.4 (86.96~116.54)	251.02 (227.01~281.66)	12.42 (11.02~14.3)	8.04 (6.87~9.23)	10.18 (9.21~11.4)
Low-middle SDI	52.18 (36.04~75.29)	28.92 (14.86~56.59)	81.11 (53.2~111.59)	15.98 (11.17~22.67)	9.84 (5.21~18.19)	12.97 (8.69~17.57)	113.93 (92.96~146.18)	73.3 (51.99~107.11)	187.23 (149.87~235.31)	16.69 (13.6~21.29)	10.54 (7.53~15.03)	13.56 (10.9~16.84)
Low SDI	27.27 (15.59~42.73)	14.12 (6.18~32.92)	41.39 (22.47~63.11)	20 (12.09~30.45)	12.05 (5.6~25.45)	16.14 (9.28~23.73)	56.66 (40.56~77.62)	32.42 (20.55~55.33)	89.08 (62.68~119.76)	19.28 (14.27~25.77)	12.27 (8.1~19.55)	15.8 (11.57~20.29)
World Bank Income Level												
World Bank High Income	321.94 (300.93~348.49)	312.1 (272.9~363.22)	634.04 (584.52~700.54)	61.79 (57.39~66.93)	39.16 (34.27~45.77)	49.58 (45.5~54.71)	511.77 (459.64~554.33)	598.84 (492.83~690.63)	1110.61 (949.42~1219.26)	50.31 (45.51~54.47)	37.37 (31.43~42.91)	43.65 (38.18~47.78)
World Bank Upper Middle Income	111.22 (97.79~125.66)	71.46 (58.47~92.05)	182.67 (159.1~211.25)	14.33 (12.7~16.1)	8.81 (7.28~11.08)	11.51 (10.08~13.17)	211.57 (190.72~237.53)	153.65 (135.97~174.46)	365.22 (333.42~407.17)	13.88 (12.46~15.56)	8.92 (7.89~10.11)	11.33 (10.34~12.61)
World Bank Lower Middle Income	82.7 (56.72~117.64)	48.72 (24.81~92.65)	131.42 (85.63~180.64)	14.76 (10.3~20.8)	9.17 (4.77~16.57)	11.99 (7.97~16.19)	184.94 (148.61~233.15)	121.24 (86.94~170.48)	306.18 (249.81~378.62)	15.43 (12.38~19.42)	10.11 (7.31~13.89)	12.74 (10.46~15.58)
World Bank Low Income	17.46 (10.32~26.88)	9.71 (4.89~22.31)	27.17 (15.88~41.61)	21.07 (13.23~31.79)	12.17 (6.65~24.55)	16.53 (10.36~24.3)	35.13 (24.73~48.74)	19.49 (12.91~33.59)	54.63 (38.98~75.56)	20.03 (14.65~27.02)	11.3 (7.79~17.93)	15.53 (11.55~20.75)
Region												
Andean Latin America	1.99 (1.52~2.61)	1.31 (0.85~1.86)	3.3 (2.47~4.28)	16.82 (12.88~22)	10.64 (6.98~14.46)	13.67 (10.36~17.42)	4.58 (3.59~5.73)	2.68 (2.12~3.37)	7.26 (5.88~8.87)	16.26 (12.83~20.32)	9.01 (7.13~11.27)	12.54 (10.21~15.28)
Australasia	6.84 (6.24~7.41)	5.31 (4.57~6.24)	12.15 (11.12~13.48)	68.98 (63.01~74.92)	38.66 (33.28~45.57)	52.52 (47.85~58.16)	12.6 (10.89~14.14)	11.37 (9.24~13.43)	23.97 (20.44~27.5)	54.42 (47.2~60.87)	35.92 (29.82~42.63)	44.54 (38.48~51.02)
Caribbean	4.36 (3.74~4.97)	1.95 (1.54~2.63)	6.31 (5.37~7.23)	32.07 (27.67~36.46)	13.73 (10.94~17.91)	22.61 (19.31~25.69)	7.35 (6.04~8.85)	3.98 (3.07~5.61)	11.32 (9.16~14.16)	30.35 (24.97~36.51)	14.62 (11.25~20.85)	22.09 (17.87~27.65)
Central Asia	1.11 (0.95~1.26)	0.64 (0.49~0.75)	1.76 (1.48~1.96)	5.2 (4.34~6.01)	2.23 (1.71~2.63)	3.48 (2.89~3.92)	3.41 (2.81~4.36)	2.13 (1.76~2.78)	5.54 (4.67~6.97)	9.99 (8.34~12.66)	5.37 (4.44~7.1)	7.45 (6.27~9.38)
Central Europe	12.88 (11.26~15.63)	8.12 (6.26~10.31)	20.99 (18.13~25.38)	20.47 (17.81~25.23)	9.77 (7.55~12.31)	14.59 (12.57~17.61)	41.63 (33.6~49.21)	42.47 (34.06~52)	84.11 (69.74~99.02)	47.43 (38.03~55.99)	31.61 (25.3~38.91)	39.15 (32.37~46.15)
Central Latin America	11.78 (10.63~12.65)	6.35 (5.18~7.77)	18.12 (16.45~20.01)	24.89 (22.56~26.86)	13.17 (10.89~15.99)	18.85 (17.19~20.94)	26.8 (21.59~33.45)	18.07 (14.49~23.94)	44.87 (36.84~55.56)	23.85 (19.28~29.74)	14.18 (11.37~18.77)	18.72 (15.4~23.13)
Central Sub-Saharan Africa	2.88 (1.57~5.32)	1.71 (0.86~3.97)	4.59 (2.59~7.53)	24.16 (14.68~42.41)	15.1 (8.1~31.22)	19.38 (11.77~30.04)	6.48 (4.05~10.65)	3.94 (2.6~6.74)	10.43 (6.84~15.6)	23.99 (16.3~36.81)	14.48 (9.81~23.15)	18.97 (13.23~26.91)
East Asia	29.92 (20.38~40.45)	17.06 (8.83~29.8)	46.99 (30.3~66.13)	5.94 (4.11~7.94)	3.5 (1.84~5.96)	4.71 (3.06~6.62)	55.07 (43.26~69.63)	31.74 (24.43~39.46)	86.82 (70.96~103.74)	5.76 (4.6~7.16)	3.07 (2.37~3.81)	4.36 (3.59~5.18)
Eastern Europe	8.32 (6.45~9.83)	5.9 (4.1~6.84)	14.22 (11.03~16.35)	8.42 (6.65~9.85)	3.4 (2.38~3.93)	5.25 (4.11~6.02)	29.01 (22.55~36.68)	17.11 (13.93~20.94)	46.12 (37.77~56.57)	23.15 (18.19~29.31)	8.09 (6.53~10.19)	14.17 (11.57~17.34)
Eastern Sub-Saharan Africa	9.82 (5.65~14.4)	5.86 (2.87~13.31)	15.69 (9.22~24.32)	22.59 (14.14~32.65)	15.05 (8.55~29.51)	18.86 (12.21~27.88)	18.82 (13.75~26.02)	11.15 (7.88~16.49)	29.97 (22.89~39.84)	20.54 (15.45~28.31)	13.36 (9.6~18.69)	16.91 (13.45~21.67)
High-income Asia Pacific	34.26 (31.85~38.06)	36.64 (31.16~45.45)	70.9 (63.43~81.93)	43.56 (39.89~48.49)	33.23 (28.12~41.23)	38.17 (33.96~44.37)	51.49 (43.83~58.02)	90.64 (64.56~109.85)	142.13 (109.6~165.94)	24.61 (21.03~27.68)	23.21 (17.78~27.46)	24.46 (19.89~28.22)
High-income North America	109.06 (102.39~116.75)	97.65 (84.74~110.92)	206.71 (189.98~224.97)	75.13 (70.26~80.38)	43.77 (38.39~49.86)	57.8 (53.37~62.81)	153.24 (137.21~166.81)	161.57 (134.8~183.81)	314.81 (274.62~346.35)	55.42 (49.7~60.13)	39.54 (33.4~45.19)	46.8 (41.53~51.25)
North Africa and Middle East	27.76 (20.25~37.09)	21.31 (13.35~32.5)	49.07 (36.29~61.47)	26.42 (19.41~35.22)	22.82 (14.04~33.61)	24.77 (18.02~31.02)	57.57 (43.05~70.16)	43.4 (34.34~54.03)	100.97 (84.2~119.65)	22.65 (17.4~27.32)	19.39 (15.36~23.64)	21.11 (17.59~24.72)
Oceania	0.41 (0.24~0.66)	0.14 (0.07~0.26)	0.54 (0.32~0.83)	22.99 (14.19~35.82)	8.29 (4.5~15.33)	15.64 (9.68~23.28)	0.92 (0.58~1.41)	0.35 (0.2~0.66)	1.27 (0.82~1.89)	22.02 (14.92~32.32)	9.08 (5.62~15.57)	15.58 (10.6~22.16)
South Asia	49.83 (30.99~78.1)	27.03 (10.29~59.44)	76.87 (44.62~111.6)	16.29 (10.43~25.03)	11.16 (4.47~22.5)	13.86 (8.35~19.67)	113.43 (85.7~161.11)	77.25 (48.78~115.63)	190.68 (144.06~248.6)	16.14 (12.32~22.5)	11.46 (7.4~16.6)	13.83 (10.63~17.66)
Southeast Asia	10.26 (8.05~14.83)	6.87 (4.97~9.84)	17.13 (14.08~23.37)	7.69 (6~11.16)	4.88 (3.57~6.67)	6.22 (5.14~8.24)	23.66 (18.26~34.39)	15.72 (12.12~18.98)	39.38 (32.23~50.17)	8.15 (6.31~11.67)	4.98 (3.86~5.97)	6.49 (5.36~8.18)
Southern Latin America	12.45 (11.1~14.1)	11.78 (9.98~13.52)	24.23 (21.71~27.03)	61.64 (55~69.69)	47.18 (40.21~53.84)	54.79 (48.95~61.1)	21.01 (19.06~22.94)	21.47 (18.69~24.43)	42.48 (38.29~46.44)	58.83 (53.32~64.23)	42.5 (37.24~48.25)	50.54 (45.69~55.27)
Southern Sub-Saharan Africa	3.21 (2.65~3.9)	2.55 (2.07~3.51)	5.76 (4.93~7.02)	21.62 (17.93~25.93)	14.68 (12.27~19.09)	17.96 (15.51~21.18)	5.95 (5~7.19)	3.79 (2.95~4.47)	9.74 (8.36~11.08)	23.98 (20.66~27.89)	12.16 (9.66~14.19)	17.24 (14.86~19.32)
Tropical Latin America	27.32 (24.46~29.6)	15.82 (12.48~19.36)	43.14 (38.39~47.88)	53.01 (47.46~56.75)	30.49 (24.41~36.23)	41.46 (36.63~45.36)	39.18 (35.4~44.29)	31.55 (27.03~39.01)	70.73 (63.57~80.92)	35.73 (32.12~40.23)	23.95 (20.52~29.57)	29.5 (26.5~33.74)
Western Europe	157.76 (146.96~170.87)	164.02 (143.33~191.92)	321.79 (294.95~357.15)	68.54 (63.73~74.1)	43.9 (38.46~51.7)	55.48 (50.82~61.36)	249.71 (224.03~270.26)	294.07 (245.2~337.05)	543.78 (471.46~598.09)	60.12 (54.05~65.02)	44.16 (37.6~50.43)	51.94 (45.69~56.95)
Western Sub-Saharan Africa	11.41 (5.83~18.35)	4.22 (1.76~10.77)	15.63 (8.04~24.98)	20.23 (10.4~32.37)	8.94 (4~21.12)	15.2 (8~23.99)	22.08 (15.16~32)	9.27 (5.4~17.95)	31.35 (21.26~45.26)	18.14 (12.51~26.24)	8.58 (5.37~15.16)	13.17 (9.11~18.49)

CAVD, calcific aortic valve disease; ASDALYR, the age-standardized disability-adjusted life years rate; SDI, socio-demographic index.

**Table 5:** The temporal trends of ASIR, ASPR, ASDR, and ASDALYR from 1990 to 2019 of CAVD.

	**EAPC of ASIR No. (95% CI)**			**EAPC of ASPR No. (95% CI)**			**EAPC of ASDR No. (95% CI)**			**EAPC of ASDALYR No. (95% CI)**		
	**Male**	**Female**	**Both**	**Male**	**Female**	**Both**	**Male**	**Female**	**Both**	**Male**	**Female**	**Both**
Global	3.04 (2.87~3.21)	3.06 (2.75~3.38)	3.03 (2.8~3.27)	3.67 (3.46~3.88)	3.62 (3.31~3.93)	3.65 (3.4~3.91)	0.05 (−0.06~0.16)	0.09 (−0.03~0.21)	0.06 (−0.04~0.15)	−0.5 (−0.61~−0.39)	−0.37 (−0.44~−0.3)	−0.45 (−0.52~−0.38)
SDI level												
High SDI	2.71 (2.45~2.98)	3.15 (2.81~3.49)	2.93 (2.62~3.23)	2.78 (2.54~3.01)	3.26 (2.96~3.57)	3.05 (2.78~3.32)	−0.34 (−0.53~−0.16)	0 (−0.1~0.1)	−0.14 (−0.27~−0.02)	−1.09 (−1.32~−0.87)	−0.6 (−0.71~−0.48)	−0.84 (−1.01~−0.67)
High–middle SDI	4.94 (4.83~5.04)	5.31 (4.98~5.65)	5.1 (4.92~5.27)	5.8 (5.63~5.98)	6.49 (6.12~6.87)	6.11 (5.87~6.35)	0.96 (0.75~1.17)	1.38 (1.06~1.71)	1.17 (0.89~1.45)	0.29 (0.17~0.4)	0.79 (0.59~0.99)	0.51 (0.36~0.67)
Middle SDI	3.56 (3.14~3.98)	4.57 (4.11~5.03)	3.92 (3.5~4.35)	7.65 (7.36~7.95)	9.3 (8.95~9.64)	8.19 (7.9~8.49)	0.06 (−0.01~0.13)	0 (−0.08~0.08)	0.01 (−0.06~0.08)	−0.12 (−0.19~−0.04)	−0.2 (−0.28~−0.11)	−0.17 (−0.24~−0.1)
Low–middle SDI	0.75 (0.64~0.87)	1.22 (1.04~1.4)	0.83 (0.69~0.97)	3.64 (3.46~3.82)	4.94 (4.7~5.18)	3.97 (3.76~4.17)	0.16 (0.09~0.24)	0.22 (0.13~0.31)	0.16 (0.08~0.24)	0.09 (0.04~0.15)	0.13 (0.04~0.23)	0.08 (0.01~0.14)
Low SDI	0 (−0.04~0.04)	0.49 (0.41~0.56)	0.12 (0.07~0.17)	1.47 (1.4~1.54)	1.85 (1.78~1.92)	1.51 (1.45~1.57)	0.01 (−0.02~0.05)	0.17 (0.09~0.25)	0.07 (0.01~0.12)	−0.19 (−0.23~−0.15)	−0.03 (−0.13~0.07)	−0.15 (−0.21~−0.09)
World Bank Income Level												
World Bank High Income	3.03 (2.73~3.32)	3.56 (3.18~3.95)	3.28 (2.94~3.63)	3.13 (2.84~3.43)	3.73 (3.37~4.08)	3.45 (3.13~3.78)	−0.06 (−0.21~0.08)	0.27 (0.17~0.36)	0.12 (0.02~0.22)	−0.81 (−0.98~−0.64)	−0.31 (−0.4~−0.22)	−0.56 (−0.68~−0.44)
World Bank Upper Middle Income	5.14 (4.76~5.51)	5.03 (4.73~5.34)	5.1 (4.76~5.44)	6.93 (6.65~7.22)	7.5 (7.2~7.79)	7.13 (6.86~7.4)	0.03 (−0.04~0.1)	0.15 (0.1~0.2)	0.07 (0.01~0.12)	−0.08 (−0.16~0)	0.05 (−0.03~0.12)	−0.04 (−0.11~0.03)
World Bank Lower Middle Income	1.24 (1.05~1.42)	1.47 (1.24~1.7)	1.28 (1.08~1.47)	3.42 (2.97~3.86)	2.87 (2.37~3.38)	3.24 (2.8~3.69)	0.15 (0.08~0.21)	0.36 (0.25~0.46)	0.23 (0.15~0.31)	0.07 (0.02~0.12)	0.18 (0.09~0.27)	0.1 (0.03~0.16)
World Bank Low Income	0.06 (0.04~0.08)	0.42 (0.38~0.45)	0.17 (0.14~0.21)	1.81 (1.72~1.89)	2.3 (2.13~2.48)	1.95 (1.83~2.06)	−0.01 (−0.05~0.03)	−0.07 (−0.14~0)	−0.05 (−0.11~0)	−0.24 (−0.3~−0.19)	−0.35 (−0.46~−0.24)	−0.29 (−0.37~−0.21)
Region												
Andean Latin America	5.97 (5.43~6.52)	3.72 (3.24~4.19)	5.3 (4.78~5.82)	9.3 (8.14~10.48)	7.35 (6.26~8.45)	8.76 (7.62~9.91)	−0.03 (−0.14~0.08)	−0.76 (−0.94~−0.58)	−0.34 (−0.46~−0.22)	−0.03 (−0.14~0.07)	−0.82 (−1~−0.65)	−0.35 (−0.47~−0.23)
Australasia	7.65 (7.33~7.96)	7.86 (7.69~8.04)	7.72 (7.51~7.94)	10.25 (9.63~10.88)	10.22 (9.84~10.6)	10.18 (9.7~10.67)	−0.59 (−0.84~−0.34)	−0.43 (−0.63~−0.23)	−0.49 (−0.71~−0.27)	−0.99 (−1.3~−0.68)	−0.45 (−0.72~−0.18)	−0.75 (−1.04~−0.45)
Caribbean	3.87 (3.74~3.99)	3.27 (3.1~3.43)	3.75 (3.63~3.87)	7.36 (7.01~7.71)	6.94 (6.43~7.45)	7.25 (6.88~7.61)	−0.08 (−0.29~0.13)	0.53 (0.33~0.72)	0.12 (−0.09~0.32)	−0.2 (−0.43~0.04)	0.42 (0.26~0.58)	−0.01 (−0.22~0.19)
Central Asia	5.5 (5.12~5.88)	4.43 (3.58~5.28)	5.26 (4.72~5.8)	7.59 (7.17~8.02)	5.57 (4.58~6.57)	6.92 (6.29~7.55)	2.81 (2.54~3.08)	4.54 (3.99~5.09)	3.72 (3.32~4.12)	2.64 (2.4~2.88)	3.57 (3.19~3.95)	3.08 (2.79~3.36)
Central Europe	6.03 (5.8~6.26)	6.87 (6.64~7.09)	6.43 (6.21~6.66)	6.47 (6.26~6.69)	7.35 (7.14~7.56)	6.92 (6.71~7.12)	4.06 (3.7~4.43)	5.6 (5.13~6.08)	4.85 (4.43~5.27)	3.41 (3.07~3.74)	4.81 (4.44~5.17)	4.02 (3.68~4.36)
Central Latin America	3.49 (3.16~3.81)	3.6 (3.19~4.02)	3.5 (3.16~3.83)	5.89 (5.39~6.4)	6.97 (6.11~7.83)	6.17 (5.57~6.78)	0.01 (−0.25~0.28)	0.53 (0.27~0.78)	0.2 (−0.06~0.46)	−0.22 (−0.49~0.05)	0.34 (0.07~0.6)	−0.04 (−0.31~0.23)
Central Sub–Saharan Africa	0.01 (−0.07~0.09)	0.26 (0.18~0.35)	0.12 (0.02~0.21)	1.17 (0.94~1.4)	0.79 (0.64~0.93)	1.06 (0.85~1.27)	0.2 (0.14~0.25)	−0.02 (−0.13~0.09)	0.08 (0~0.17)	−0.05 (−0.12~0.02)	−0.25 (−0.36~−0.14)	−0.13 (−0.22~−0.04)
East Asia	7.14 (6.4~7.89)	7.96 (7.2~8.72)	7.5 (6.75~8.26)	11.67 (11.02~12.32)	11.46 (10.83~12.08)	11.5 (10.86~12.13)	−0.01 (−0.29~0.26)	−0.96 (−1.36~−0.55)	−0.54 (−0.86~−0.21)	−0.17 (−0.28~−0.06)	−0.87 (−1.1~−0.64)	−0.48 (−0.63~−0.34)
Eastern Europe	7.55 (7.14~7.95)	8.26 (7.68~8.83)	7.75 (7.33~8.16)	8.04 (7.62~8.46)	9.05 (8.44~9.68)	8.39 (7.97~8.81)	2.66 (2.34~2.99)	3.84 (3.44~4.24)	3.46 (3.16~3.77)	3.77 (3.46~4.08)	3.59 (3.26~3.93)	3.79 (3.5~4.08)
Eastern Sub–Saharan Africa	0.03 (−0.07~0.13)	−0.03 (−0.09~0.04)	−0.02 (−0.1~0.07)	1.72 (1.46~1.98)	0.73 (0.65~0.81)	1.39 (1.19~1.6)	−0.09 (−0.14~−0.03)	−0.15 (−0.31~0.02)	−0.13 (−0.24~−0.02)	−0.45 (−0.52~−0.38)	−0.52 (−0.71~−0.33)	−0.5 (−0.61~−0.38)
High–income Asia Pacific	1.65 (1.45~1.86)	1.51 (1.4~1.62)	1.55 (1.43~1.68)	2.11 (1.99~2.23)	1.39 (1.26~1.53)	1.71 (1.61~1.81)	−1.21 (−1.65~−0.78)	−0.19 (−0.62~0.25)	−0.49 (−0.92~−0.06)	−1.83 (−2.26~−1.39)	−1 (−1.41~−0.58)	−1.32 (−1.74~−0.9)
High–income North America	1.34 (0.82~1.85)	1.58 (1.01~2.15)	1.43 (0.89~1.98)	0.84 (0.3~1.39)	1.15 (0.57~1.74)	1.01 (0.45~1.57)	−0.51 (−0.78~−0.24)	−0.08 (−0.28~0.12)	−0.28 (−0.49~−0.07)	−1.43 (−1.78~−1.09)	−0.83 (−1.05~−0.61)	−1.15 (−1.42~−0.88)
North Africa and Middle East	0.04 (−0.27~0.35)	1.28 (1.09~1.47)	0.42 (0.16~0.68)	1.7 (1.29~2.11)	4.19 (3.93~4.45)	2.19 (1.84~2.55)	−0.56 (−0.64~−0.48)	−0.69 (−0.77~−0.61)	−0.65 (−0.7~−0.61)	−0.66 (−0.73~−0.6)	−0.75 (−0.82~−0.68)	−0.71 (−0.77~−0.65)
Oceania	0.52 (−0.13~1.17)	1.84 (1.51~2.17)	0.9 (0.34~1.45)	2.67 (1.29~4.06)	5.48 (4.85~6.13)	3.44 (2.28~4.62)	−0.46 (−0.53~−0.39)	0.14 (0.05~0.24)	−0.28 (−0.36~−0.2)	−0.28 (−0.35~−0.22)	0.23 (0.15~0.31)	−0.13 (−0.19~−0.06)
South Asia	0.29 (0.26~0.32)	0.86 (0.75~0.97)	0.31 (0.26~0.36)	1.48 (1.43~1.53)	2.23 (2.11~2.34)	1.52 (1.49~1.55)	−0.09 (−0.17~−0.01)	−0.11 (−0.24~0.02)	−0.11 (−0.2~−0.01)	−0.1 (−0.16~−0.04)	−0.07 (−0.18~0.03)	−0.12 (−0.19~−0.05)
Southeast Asia	1.39 (1.15~1.63)	1.99 (1.69~2.28)	1.66 (1.39~1.93)	5.82 (5.41~6.22)	6.45 (6.04~6.86)	6.09 (5.69~6.5)	0.31 (0.22~0.4)	0.2 (0.06~0.34)	0.25 (0.13~0.36)	0.15 (0.08~0.21)	−0.06 (−0.16~0.04)	0.06 (−0.02~0.14)
Southern Latin America	4.77 (4.4~5.14)	3.24 (2.84~3.64)	3.96 (3.58~4.35)	8.16 (7.46~8.87)	6.67 (6.08~7.26)	7.44 (6.81~8.08)	0.27 (0.13~0.41)	−0.34 (−0.43~−0.24)	−0.11 (−0.21~−0.01)	−0.13 (−0.28~0.02)	−0.49 (−0.59~−0.39)	−0.33 (−0.45~−0.22)
Southern Sub–Saharan Africa	6.01 (4.87~7.16)	−0.5 (−0.56~−0.44)	5.08 (4.01~6.15)	9.62 (8.13~11.14)	−0.13 (−0.27~0)	9.15 (7.62~10.7)	0.56 (0.36~0.76)	−0.22 (−0.38~−0.07)	0.06 (−0.11~0.23)	0.09 (−0.1~0.28)	−0.88 (−1.04~−0.72)	−0.41 (−0.58~−0.24)
Tropical Latin America	0.71 (0.52~0.9)	0.23 (0.12~0.34)	0.48 (0.32~0.64)	3.39 (3.11~3.68)	3.28 (3.13~3.43)	3.31 (3.08~3.54)	−0.42 (−0.61~−0.22)	−0.21 (−0.33~−0.1)	−0.36 (−0.51~−0.2)	−1.23 (−1.44~−1.01)	−0.73 (−0.89~−0.57)	−1.05 (−1.25~−0.86)
Western Europe	5.65 (4.96~6.34)	7.12 (6.12~8.12)	6.35 (5.5~7.2)	5.76 (5.03~6.5)	7.4 (6.4~8.41)	6.6 (5.72~7.49)	0.41 (0.31~0.5)	0.55 (0.47~0.64)	0.47 (0.4~0.54)	−0.51 (−0.61~−0.4)	−0.08 (−0.15~−0.01)	−0.31 (−0.38~−0.24)
Western Sub–Saharan Africa	−0.06 (−0.11~−0.01)	0.55 (0.51~0.59)	−0.03 (−0.07~0.01)	0.98 (0.83~1.13)	1 (0.96~1.03)	0.72 (0.58~0.85)	−0.48 (−0.57~−0.39)	0.04 (−0.01~0.08)	−0.44 (−0.51~−0.37)	−0.57 (−0.67~−0.47)	−0.23 (−0.31~−0.15)	−0.68 (−0.78~−0.59)

ASIR, the age-standardized incidence rate; ASPR, the age-standardized prevalence rate; ASDR, the age-standardized death rate; ASDALYR, the age-standardized disability-adjusted life years rate; CAVD, calcific aortic valve disease; SDI, socio-demographic index.

Compared with female individuals, male individuals contributed more in ASDALYR until 2019 (male: 31.5 per 100,000 in 1990 and 27.4 in 2019; female: 22.3 per 100,000 in 1990 and 20.5 in 2019) globally (Figure 4A; Table 4). Subgroup analysis by SDI, WBI levels, and geographical regions all showed that male individuals had a higher burden of disability than female individuals. Nevertheless, male individuals showed a more pronounced downward trend than female individuals (EAPC of male individuals: −0.5, 95% CI −0.61~−0.39; EAPC of female individuals: −0.37, 95% CI −0.44~−0.3) (Figure 4D; Table 5).

### Attributable risks

Three attributable risk factors of CAVD were available in GBD 2019, including lead exposure, high SBP, and a diet high in sodium.

**Figure 5:**
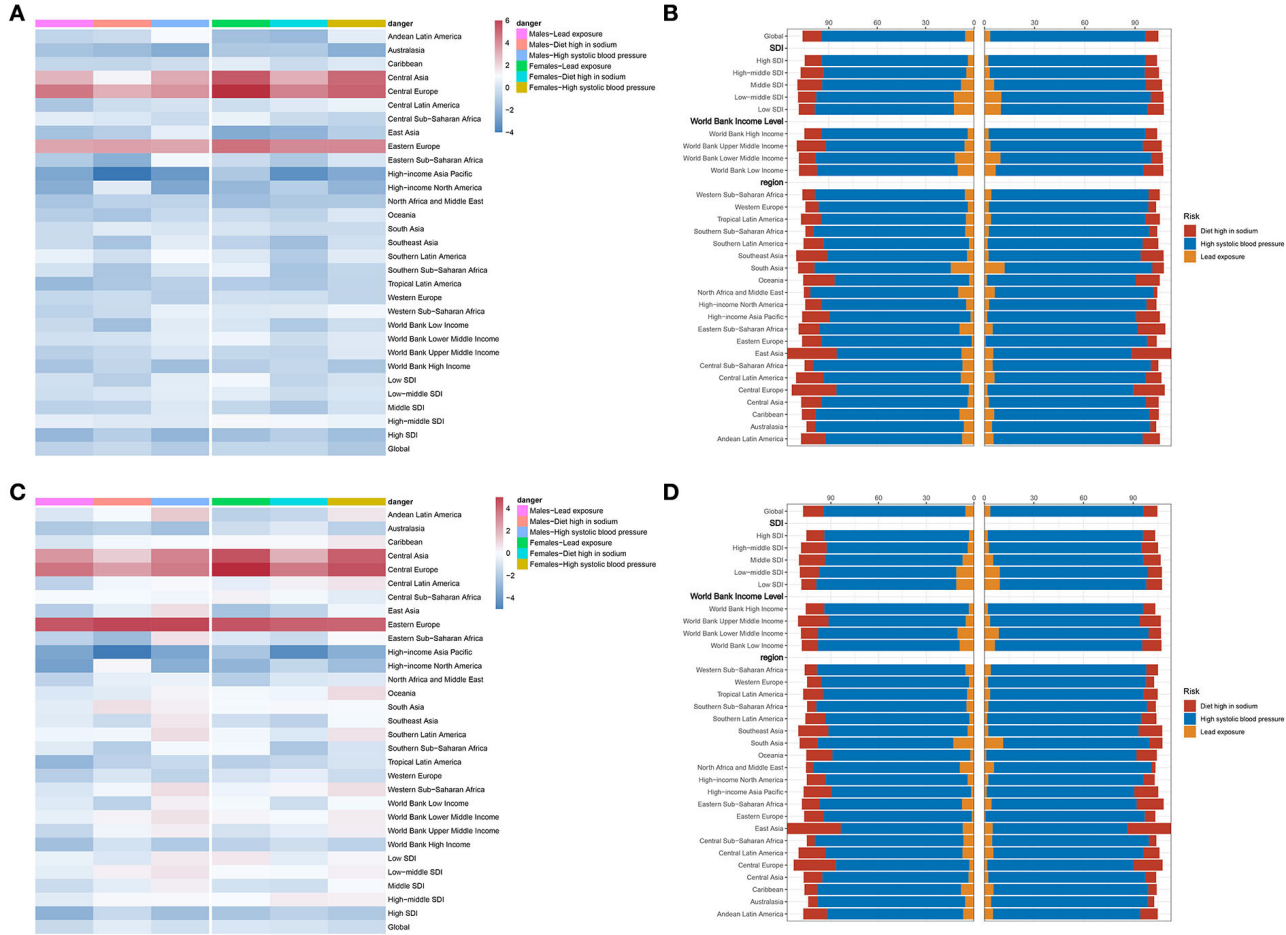
Attributable risks of CAVD. **(A)** EAPC and attributable risks of ASDR in 31 regions. **(B)** PAF of attributable risks of ASDR in 2019. **(C)** EAPC and attributable risks of ASDALYR in 31 regions. **(D)** PAF of attributable risks of ASDALYR in 2019. CAVD, calcific aortic valve disease; EAPC, estimated annual percentage change; ASDR, age-standardized death rate; PAF, population attributable fraction; ASDALYR, the age-standardized disability-adjusted life years rate.

First of all, lead exposure had a downtrend globally (EAPC = −0.36, 95% CI −0.5~−0.23, *P* < 0.001) (Table 6). In detail, ASDR caused by lead exposure had the fastest reduction in high-income North America (EAPC = −1.45, 95% CI −1.65~−1.25, *P* < 0.001), while it had a biggest rise in Central Europe (EAPC = 4.32, 95% CI 3.78~4.86, *P* < 0.001). Taking sex into consideration, male individuals in high-income North America had the fastest reduction (EAPC = −0.36, 95% CI −0.5~−0.23, *P* < 0.001), but female individuals in Central Europe showedthe most rapid increase (EAPC = 5.48, 95% CI 4.93~6.04, *P* < 0.001) (Figure 5A; Table 6). Second, ASDR from a diet high in sodium also showed a downtrend in the globe (EAPC = −0.17, 95% CI −0.27~−0.07, *P* = 0.001), especially in high-income Asia Pacific (EAPC = −2.92, 95% CI −3.41~−2.42, *P* < 0.001) (Table 6). However, Eastern Europe showed the most rapid increase with an EAPC of 3.42 (95% CI 3.04~3.79, *P* < 0.001). As for sex stratification, male individuals in high-income Asia Pacific showed the fastest decrease (EAPC = −3.42, 95% CI −3.95~−2.89, *P* < 0.001), whereas female individuals in Central Europe presented the fastest increase (EAPC = 3.69, 95% CI 3.4~4, *P* < 0.001) (Figure 5A; Table 6). At last, high SBP, another attributable risk, also showed a downtrend around the world (EAPC = −0.68, 95% CI −0.77~−0.59, *P* < 0.001), especially in high-income Asia Pacific (EAPC = −1.9, 95% CI −2.3~−1.5, *P* < 0.001) (Table 6). Nevertheless, ASDR of CAVD resulting from high SBP in Central Europe appeared as the biggest rise (EAPC = 3.81, 95% CI 3.43~4.19, *P* < 0.001). Meanwhile, males in high-income Asia Pacific had the sharpest decline (EAPC = −2.29, 95% CI −2.73~−1.85, *P* < 0.001), while females in Central Europe presented the fastest increase (EAPC = 4.37, 95% CI 3.95~4.8, *P* < 0.001) (Figure 5A; Table 6).

**Table 6:** Attributable risks of ASDR in CAVD.

	**Lead exposure**			**Diet high in sodium**			**High systolic blood pressure**		
	**Male**	**Female**	**Both**	**Male**	**Female**	**Both**	**Male**	**Female**	**Both**
Global	−0.39 (−0.49~−0.28)	−0.22 (−0.40~−0.03)	−0.36 (−0.50~−0.23)	−0.14 (−0.27~−0.01)	−0.22 (−0.30~−0.14)	−0.17 (−0.27~−0.07)	−0.61 (−0.73~−0.49)	−0.71 (−0.83~−0.58)	−0.68 (−0.77~−0.59)
SDI level									
High SDI	−1.27 (−1.42~−1.12)	−0.99 (−1.15~−0.82)	−1.15 (−1.29~−1.02)	−0.53 (−0.76~−0.30)	−0.51 (−0.68~−0.34)	−0.46 (−0.67~−0.26)	−1.33 (−1.58~−1.09)	−1.05 (−1.20~−0.90)	−1.17 (−1.34~−0.99)
High–middle SDI	0.65 (0.33~0.98)	1.14 (0.68~1.61)	0.78 (0.40~1.17)	0.48 (0.37~0.59)	0.92 (0.75~1.10)	0.66 (0.53~0.78)	0.48 (0.24~0.71)	0.76 (0.41~1.10)	0.62 (0.32~0.92)
Middle SDI	−0.40 (−0.48~−0.33)	−0.22 (−0.28~−0.15)	−0.39 (−0.46~−0.32)	−0.33 (−0.43~−0.23)	−0.75 (−0.88~−0.62)	−0.50 (−0.60~−0.41)	0.43 (0.37~0.49)	0.19 (0.15~0.23)	0.29 (0.25~0.34)
Low–middle SDI	0.10 (0.02~0.18)	0.59 (0.49~0.69)	0.22 (0.14~0.30)	0.34 (0.30~0.39)	0.01 (−0.09~0.11)	0.16 (0.10~0.22)	0.58 (0.54~0.63)	0.35 (0.28~0.42)	0.46 (0.40~0.51)
Low SDI	0.16 (0.08~0.25)	0.94 (0.90~0.98)	0.38 (0.33~0.43)	−0.48 (−0.55~−0.40)	−0.28 (−0.41~−0.16)	−0.42 (−0.52~−0.33)	0.56 (0.52~0.60)	0.27 (0.25~0.29)	0.41 (0.39~0.44)
World Bank Income Level									
World Bank High Income	−0.84 (−0.97~−0.71)	−0.54 (−0.73~−0.34)	−0.72 (−0.86~−0.58)	−0.26 (−0.45~−0.06)	−0.19 (−0.33~−0.06)	−0.18 (−0.35~−0.02)	−1.03 (−1.21~−0.85)	−0.79 (−0.93~−0.66)	−0.90 (−1.03~−0.76)
World Bank Upper Middle Income	−0.49 (−0.59~−0.39)	−0.20 (−0.30~−0.10)	−0.45 (−0.55~−0.34)	−0.17 (−0.26~−0.08)	−0.30 (−0.37~−0.22)	−0.22 (−0.29~−0.15)	0.38 (0.30~0.45)	0.45 (0.37~0.53)	0.39 (0.32~0.47)
World Bank Lower Middle Income	0.18 (0.07~0.29)	0.83 (0.72~0.95)	0.39 (0.29~0.50)	0.24 (0.20~0.27)	0.06 (−0.01~0.13)	0.14 (0.10~0.19)	0.59 (0.55~0.63)	0.48 (0.42~0.55)	0.53 (0.48~0.58)
World Bank Low Income	−0.13 (−0.21~−0.05)	0.35 (0.30~0.39)	−0.06 (−0.13~0.00)	−1.00 (−1.07~−0.93)	−0.65 (−0.80~−0.50)	−0.88 (−0.99~−0.77)	0.54 (0.52~0.55)	0.09 (0.07~0.11)	0.33 (0.31~0.34)
Region									
Andean Latin America	−0.30 (−0.40~−0.20)	−0.97 (−1.17~−0.77)	−0.54 (−0.66~−0.41)	−0.09 (−0.21~0.02)	−1.18 (−1.39~−0.97)	−0.46 (−0.59~−0.33)	1.07 (0.83~1.30)	0.60 (0.42~0.79)	0.88 (0.67~1.08)
Australasia	−0.88 (−1.08~−0.68)	−0.72 (−0.90~−0.54)	−0.79 (−0.97~−0.61)	−1.11 (−1.34~−0.87)	−0.65 (−0.84~−0.45)	−0.89 (−1.11~−0.67)	−1.64 (−1.97~−1.30)	−1.52 (−1.80~−1.25)	−1.59 (−1.89~−1.30)
Caribbean	−0.23 (−0.32~−0.15)	0.56 (0.43~0.68)	−0.11 (−0.19~−0.02)	−0.25 (−0.45~−0.05)	−0.02 (−0.19~0.14)	−0.24 (−0.43~−0.04)	0.08 (−0.12~0.28)	0.44 (0.28~0.61)	0.18 (0.00~0.37)
Central Asia	2.62 (2.34~2.91)	4.58 (4.13~5.04)	3.37 (3.04~3.70)	1.23 (0.93~1.53)	2.70 (2.05~3.36)	1.85 (1.43~2.28)	2.79 (2.49~3.10)	4.22 (3.68~4.76)	3.52 (3.11~3.94)
Central Europe	3.92 (3.37~4.47)	5.48 (4.93~6.04)	4.32 (3.78~4.86)	2.70 (2.49~2.90)	3.69 (3.40~4.00)	3.03 (2.80~3.26)	3.27 (2.92~3.61)	4.37 (3.95~4.80)	3.81 (3.43~4.19)
Central Latin America	−0.77 (−0.93~−0.62)	0.09 (−0.03~0.21)	−0.59 (−0.73~−0.44)	0.06 (−0.28~0.39)	0.48 (0.18~0.79)	0.08 (−0.24~0.41)	0.27 (0.04~0.51)	0.76 (0.51~1.00)	0.44 (0.20~0.68)
Central Sub–Saharan Africa	0.56 (0.48~0.63)	0.74 (0.70~0.77)	0.53 (0.49~0.56)	0.40 (0.36~0.44)	0.02 (−0.07~0.11)	0.19 (0.12~0.25)	0.04 (0.00~0.08)	−0.28 (−0.32~−0.24)	−0.09 (−0.13~−0.06)
East Asia	−0.93 (−1.18~−0.68)	−1.69 (−1.97~−1.41)	−1.31 (−1.57~−1.06)	−0.52 (−0.62~−0.41)	−1.42 (−1.64~−1.19)	−0.87 (−1.00~−0.73)	0.64 (0.37~0.90)	−0.53 (−0.91~−0.16)	0.05 (−0.25~0.35)
Eastern Europe	2.91 (2.46~3.35)	4.05 (3.70~4.41)	3.48 (3.07~3.89)	3.04 (2.63~3.46)	3.51 (3.10~3.91)	3.42 (3.04~3.79)	2.91 (2.54~3.28)	3.55 (3.15~3.95)	3.42 (3.10~3.74)
Eastern Sub–Saharan Africa	−0.64 (−0.72~−0.55)	−0.07 (−0.17~0.02)	−0.59 (−0.64~−0.54)	−1.52 (−1.61~−1.42)	−0.71 (−0.91~−0.52)	−1.13 (−1.27~−0.99)	0.92 (0.87~0.96)	0.34 (0.21~0.46)	0.63 (0.55~0.70)
High–income Asia Pacific	−1.83 (−2.21~−1.45)	−0.71 (−1.10~−0.31)	−1.22 (−1.61~−0.82)	−3.42 (−3.95~−2.89)	−2.59 (−3.06~−2.11)	−2.92 (−3.41~−2.42)	−2.29 (−2.73~−1.85)	−1.74 (−2.14~−1.35)	−1.90 (−2.30~−1.50)
High–income North America	−1.57 (−1.81~−1.34)	−1.25 (−1.46~−1.04)	−1.45 (−1.65~−1.25)	0.51 (0.34~0.68)	−0.50 (−0.72~−0.27)	0.14 (−0.05~0.33)	−1.80 (−2.16~−1.44)	−1.33 (−1.65~−1.02)	−1.55 (−1.83~−1.27)
North Africa and Middle East	−0.82 (−0.97~−0.67)	−1.04 (−1.17~−0.91)	−0.92 (−1.05~−0.78)	−0.41 (−0.48~−0.34)	−0.72 (−0.79~−0.65)	−0.52 (−0.57~−0.46)	−0.32 (−0.41~−0.23)	−0.67 (−0.80~−0.55)	−0.54 (−0.63~−0.44)
Oceania	−0.53 (−0.64~−0.42)	−0.05 (−0.11~0.01)	−0.46 (−0.54~−0.38)	−0.83 (−0.94~−0.72)	−0.37 (−0.50~−0.24)	−0.70 (−0.81~−0.58)	−0.15 (−0.22~−0.09)	0.41 (0.31~0.50)	0.04 (−0.03~0.11)
South Asia	−0.01 (−0.15~0.13)	0.33 (0.17~0.49)	0.07 (−0.07~0.22)	0.54 (0.46~0.62)	−0.05 (−0.13~0.03)	0.26 (0.19~0.33)	0.32 (0.24~0.40)	−0.04 (−0.15~0.07)	0.15 (0.07~0.24)
Southeast Asia	0.02 (−0.09~0.13)	−0.35 (−0.48~−0.22)	−0.16 (−0.28~−0.04)	−0.84 (−0.95~−0.72)	−1.02 (−1.18~−0.86)	−0.94 (−1.07~−0.80)	0.58 (0.53~0.64)	0.17 (0.12~0.23)	0.39 (0.34~0.44)
Southern Latin America	0.72 (0.59~0.85)	0.43 (0.30~0.56)	0.51 (0.39~0.63)	0.14 (−0.04~0.31)	−0.62 (−0.72~−0.52)	−0.28 (−0.40~−0.16)	1.09 (0.98~1.19)	0.54 (0.35~0.72)	0.74 (0.61~0.86)
Southern Sub–Saharan Africa	0.22 (−0.04~0.47)	0.72 (0.53~0.92)	0.14 (−0.07~0.36)	−0.77 (−0.96~−0.59)	−0.87 (−1.02~−0.72)	−0.89 (−1.05~−0.73)	0.33 (0.12~0.54)	−0.22 (−0.40~−0.04)	−0.02 (−0.21~0.17)
Tropical Latin America	−1.20 (−1.32~−1.08)	−0.58 (−0.64~−0.52)	−1.05 (−1.13~−0.96)	−0.75 (−0.91~−0.59)	−0.77 (−0.87~−0.68)	−0.83 (−0.96~−0.69)	−0.44 (−0.60~−0.29)	−0.30 (−0.35~−0.24)	−0.41 (−0.52~−0.30)
Western Europe	−0.20 (−0.37~−0.03)	0.16 (−0.10~0.41)	−0.06 (−0.26~0.15)	0.04 (−0.13~0.20)	0.12 (0.04~0.20)	0.08 (−0.05~0.20)	−0.52 (−0.64~−0.39)	−0.31 (−0.41~−0.21)	−0.41 (−0.51~−0.31)
Western Sub–Saharan Africa	−0.41 (−0.57~−0.25)	0.43 (0.34~0.52)	−0.37 (−0.52~−0.21)	−0.06 (−0.13~0.00)	0.50 (0.44~0.55)	−0.07 (−0.11~−0.02)	0.72 (0.62~0.83)	0.89 (0.82~0.96)	0.59 (0.49~0.69)

ASDR, the age-standardized death rate; CAVD, calcific aortic valve disease; SDI, socio-demographic index.

High SBP was considered the dominating contributor to ASRD (PAF = 89.08% in males, PAF = 92.76% in females) and ASDALY (PAF = 89% in males, PAF = 92.43% in females) globally (Figures 5B,D; **Table 8**). The details of PAF in different regions were listed in Table 8. Similar change trends and PAF were found in ASDALYR (Figure 5C; Tables 7, 8).

**Table 7:** Attributable risks of ASDALYR in CAVD.

	**Lead exposure**			**Diet high in sodium**			**High systolic blood pressure**		
	**Male**	**Female**	**Both**	**Male**	**Female**	**Both**	**Male**	**Female**	**Both**
Global	−1.13 (−1.20~−1.05)	−0.73 (−0.85~−0.60)	−1.02 (−1.11~−0.93)	−0.53 (−0.66~−0.40)	−0.79 (−0.90~−0.67)	−0.62 (−0.74~−0.51)	−0.92 (−1.06~−0.78)	−1.05 (−1.16~−0.95)	−0.99 (−1.09~−0.89)
SDI level									
High SDI	−2.45 (−2.61~−2.28)	−1.83 (−1.97~−1.69)	−2.23 (−2.37~−2.09)	−1.15 (−1.41~−0.90)	−1.37 (−1.60~−1.15)	−1.18 (−1.42~−0.93)	−2.01 (−2.31~−1.70)	−1.69 (−1.90~−1.48)	−1.84 (−2.09~−1.59)
High–middle SDI	−0.56 (−0.81~−0.31)	0.06 (−0.27~0.40)	−0.41 (−0.68~−0.13)	0.14 (0.08~0.20)	0.40 (0.32~0.48)	0.24 (0.19~0.30)	0.11 (−0.04~0.25)	0.32 (0.09~0.55)	0.21 (0.02~0.40)
Middle SDI	−1.07 (−1.15~−1.00)	−0.87 (−0.95~−0.80)	−1.07 (−1.14~−0.99)	−0.48 (−0.59~−0.38)	−0.96 (−1.12~−0.81)	−0.66 (−0.77~−0.56)	0.36 (0.29~0.43)	0.09 (0.04~0.14)	0.23 (0.17~0.28)
Low–middle SDI	−0.46 (−0.55~−0.37)	0.04 (−0.05~0.13)	−0.36 (−0.45~−0.28)	0.31 (0.25~0.36)	−0.04 (−0.15~0.07)	0.14 (0.07~0.20)	0.57 (0.53~0.61)	0.31 (0.23~0.38)	0.44 (0.39~0.48)
Low SDI	−0.37 (−0.46~−0.28)	0.48 (0.45~0.52)	−0.16 (−0.22~−0.09)	−0.68 (−0.77~−0.58)	−0.48 (−0.65~−0.31)	−0.63 (−0.75~−0.51)	0.44 (0.41~0.47)	0.17 (0.14~0.21)	0.32 (0.29~0.34)
World Bank Income Level									
World Bank High Income	−1.99 (−2.10~−1.87)	−1.38 (−1.55~−1.21)	−1.78 (−1.90~−1.66)	−0.85 (−1.05~−0.64)	−0.98 (−1.16~−0.80)	−0.84 (−1.04~−0.65)	−1.66 (−1.88~−1.43)	−1.40 (−1.57~−1.22)	−1.52 (−1.70~−1.33)
World Bank Upper Middle Income	−1.18 (−1.25~−1.10)	−0.83 (−0.89~−0.77)	−1.12 (−1.19~−1.05)	−0.22 (−0.32~−0.12)	−0.45 (−0.58~−0.33)	−0.30 (−0.40~−0.21)	0.35 (0.28~0.42)	0.35 (0.31~0.39)	0.34 (0.28~0.39)
World Bank Lower Middle Income	−0.41 (−0.54~−0.29)	0.19 (0.08~0.30)	−0.25 (−0.37~−0.14)	0.24 (0.20~0.29)	−0.05 (−0.12~0.03)	0.11 (0.07~0.16)	0.60 (0.56~0.65)	0.40 (0.35~0.45)	0.50 (0.46~0.54)
World Bank Low Income	−0.61 (−0.69~−0.53)	−0.09 (−0.14~−0.05)	−0.52 (−0.59~−0.46)	−1.40 (−1.49~−1.31)	−1.00 (−1.20~−0.80)	−1.27 (−1.40~−1.14)	0.33 (0.31~0.35)	−0.11 (−0.16~−0.07)	0.16 (0.13~0.18)
Region									
Andean Latin America	−0.71 (−0.81~−0.62)	−1.42 (−1.62~−1.22)	−0.94 (−1.06~−0.82)	−0.04 (−0.15~0.08)	−1.19 (−1.40~−0.98)	−0.39 (−0.52~−0.26)	1.15 (0.90~1.40)	0.52 (0.30~0.74)	0.91 (0.68~1.14)
Australasia	−1.70 (−1.93~−1.48)	−1.01 (−1.20~−0.82)	−1.47 (−1.68~−1.25)	−1.43 (−1.72~−1.13)	−0.64 (−0.89~−0.39)	−1.18 (−1.46~−0.89)	−1.97 (−2.36~−1.58)	−1.43 (−1.79~−1.07)	−1.75 (−2.13~−1.38)
Caribbean	−0.78 (−0.88~−0.68)	0.11 (0.01~0.20)	−0.63 (−0.72~−0.54)	−0.16 (−0.39~0.07)	0.12 (−0.02~0.27)	−0.13 (−0.33~0.08)	0.06 (−0.16~0.28)	0.46 (0.31~0.61)	0.17 (−0.03~0.36)
Central Asia	2.25 (1.97~2.52)	3.71 (3.36~4.06)	2.69 (2.41~2.98)	1.07 (0.80~1.35)	1.69 (1.17~2.22)	1.31 (0.98~1.65)	2.66 (2.37~2.94)	3.45 (3.03~3.88)	3.01 (2.68~3.34)
Central Europe	3.00 (2.45~3.54)	4.59 (4.13~5.06)	3.34 (2.83~3.85)	2.08 (1.93~2.23)	2.87 (2.68~3.06)	2.30 (2.15~2.45)	2.82 (2.50~3.13)	3.73 (3.41~4.05)	3.20 (2.89~3.52)
Central Latin America	−1.37 (−1.52~−1.22)	−0.45 (−0.57~−0.33)	−1.19 (−1.33~−1.05)	−0.19 (−0.52~0.13)	0.29 (−0.03~0.61)	−0.16 (−0.48~0.17)	0.11 (−0.14~0.37)	0.54 (0.26~0.81)	0.23 (−0.03~0.49)
Central Sub–Saharan Africa	0.04 (−0.04~0.11)	0.27 (0.23~0.31)	0.07 (0.02~0.11)	0.07 (0.01~0.13)	−0.13 (−0.22~−0.04)	−0.02 (−0.10~0.06)	−0.18 (−0.22~−0.14)	−0.52 (−0.57~−0.47)	−0.29 (−0.33~−0.25)
East Asia	−1.54 (−1.69~−1.38)	−1.90 (−2.07~−1.72)	−1.72 (−1.86~−1.58)	−0.47 (−0.54~−0.40)	−1.31 (−1.50~−1.13)	−0.76 (−0.85~−0.67)	0.69 (0.57~0.81)	−0.23 (−0.45~−0.02)	0.31 (0.17~0.45)
Eastern Europe	3.65 (3.21~4.09)	3.66 (3.35~3.97)	3.70 (3.25~4.14)	3.98 (3.58~4.39)	3.38 (3.03~3.73)	3.93 (3.55~4.32)	3.96 (3.60~4.32)	3.35 (3.00~3.70)	3.83 (3.51~4.14)
Eastern Sub–Saharan Africa	−1.37 (−1.46~−1.28)	−0.68 (−0.80~−0.57)	−1.30 (−1.37~−1.23)	−2.11 (−2.24~−1.98)	−1.08 (−1.31~−0.85)	−1.66 (−1.84~−1.48)	0.61 (0.56~0.66)	0.11 (−0.04~0.27)	0.39 (0.31~0.48)
High–income Asia Pacific	−2.85 (−3.22~−2.47)	−1.82 (−2.17~−1.46)	−2.37 (−2.74~−2.00)	−4.23 (−4.76~−3.70)	−3.68 (−4.16~−3.20)	−3.94 (−4.44~−3.43)	−2.88 (−3.33~−2.43)	−2.55 (−2.95~−2.16)	−2.66 (−3.08~−2.25)
High–income North America	−3.02 (−3.32~−2.72)	−2.33 (−2.54~−2.12)	−2.81 (−3.06~−2.55)	0.17 (0.02~0.32)	−1.23 (−1.51~−0.95)	−0.29 (−0.49~−0.09)	−2.56 (−3.05~−2.08)	−2.06 (−2.44~−1.69)	−2.32 (−2.71~−1.94)
North Africa and Middle East	−1.35 (−1.51~−1.19)	−1.50 (−1.66~−1.34)	−1.40 (−1.56~−1.25)	−0.42 (−0.47~−0.37)	−0.73 (−0.82~−0.64)	−0.50 (−0.56~−0.45)	−0.34 (−0.40~−0.29)	−0.62 (−0.74~−0.50)	−0.48 (−0.56~−0.40)
Oceania	−0.69 (−0.81~−0.57)	−0.13 (−0.21~−0.05)	−0.60 (−0.70~−0.50)	−0.58 (−0.70~−0.46)	−0.21 (−0.33~−0.10)	−0.46 (−0.57~−0.35)	0.21 (0.09~0.33)	0.75 (0.62~0.87)	0.39 (0.27~0.50)
South Asia	−0.51 (−0.65~−0.37)	−0.06 (−0.19~0.07)	−0.42 (−0.54~−0.29)	0.67 (0.57~0.76)	0.14 (0.04~0.23)	0.43 (0.35~0.51)	0.33 (0.25~0.42)	0.04 (−0.03~0.12)	0.19 (0.13~0.26)
Southeast Asia	−0.54 (−0.68~−0.39)	−0.95 (−1.11~−0.79)	−0.70 (−0.84~−0.55)	−1.09 (−1.20~−0.98)	−1.39 (−1.53~−1.24)	−1.21 (−1.33~−1.09)	0.50 (0.46~0.53)	−0.07 (−0.11~−0.04)	0.26 (0.24~0.29)
Southern Latin America	−0.21 (−0.35~−0.08)	−0.14 (−0.27~0.00)	−0.26 (−0.39~−0.13)	−0.19 (−0.39~0.01)	−0.70 (−0.80~−0.60)	−0.43 (−0.57~−0.28)	0.73 (0.64~0.83)	0.57 (0.38~0.76)	0.62 (0.52~0.72)
Southern Sub–Saharan Africa	−0.54 (−0.77~−0.30)	−0.02 (−0.19~0.14)	−0.62 (−0.82~−0.41)	−1.50 (−1.71~−1.30)	−1.74 (−1.91~−1.57)	−1.65 (−1.83~−1.46)	−0.13 (−0.33~0.06)	−0.82 (−0.99~−0.65)	−0.49 (−0.67~−0.32)
Tropical Latin America	−2.32 (−2.44~−2.21)	−1.47 (−1.53~−1.41)	−2.12 (−2.21~−2.02)	−1.38 (−1.56~−1.20)	−1.21 (−1.34~−1.08)	−1.39 (−1.55~−1.22)	−1.15 (−1.32~−0.98)	−0.80 (−0.89~−0.71)	−1.04 (−1.18~−0.90)
Western Europe	−1.52 (−1.68~−1.36)	−0.81 (−1.06~−0.55)	−1.27 (−1.46~−1.08)	−0.75 (−0.95~−0.55)	−0.45 (−0.55~−0.34)	−0.63 (−0.79~−0.46)	−1.36 (−1.52~−1.20)	−1.05 (−1.18~−0.91)	−1.21 (−1.35~−1.07)
Western Sub–Saharan Africa	−0.75 (−0.91~−0.58)	−0.28 (−0.36~−0.20)	−0.91 (−1.08~−0.74)	−0.18 (−0.26~−0.10)	0.17 (0.10~0.25)	−0.34 (−0.40~−0.27)	0.70 (0.60~0.80)	0.67 (0.61~0.72)	0.45 (0.36~0.55)

ASDALYR, the age-standardized disability-adjusted life years rate; CAVD, calcific aortic valve disease; SDI, socio-demographic index.

**Table 8:** PAF of attributable risks of ASDR and ASDALYR to CAVD in 2019.

	**ASDR (%)**						**ASDALYR (%)**					
	**Lead exposure**		**Diet high**		**High systolic**		**Lead exposure**		**Diet high**		**High systolic**	
			**in sodium**		**blood pressure**				**in sodium**		**blood pressure**	
	**Male**	**Female**	**Male**	**Female**	**Male**	**Female**	**Male**	**Female**	**Male**	**Female**	**Male**	**Female**
Global	5.53	3.49	11.63	7.68	89.08	92.76	5.38	3.67	13.05	8.55	89.00	92.43
SDI level
High SDI	3.78	2.34	10.54	7.03	90.59	93.75	3.13	2.09	11.41	7.40	90.89	93.80
High-middle SDI	4.88	3.18	14.07	8.56	88.51	92.55	3.95	2.77	16.16	10.06	88.61	92.24
Middle SDI	8.10	5.81	15.32	9.59	86.03	90.72	7.14	5.37	16.31	10.69	86.61	90.62
Low-middle SDI	12.61	10.14	11.26	7.75	85.19	89.13	11.10	9.37	12.02	8.42	86.36	89.58
Low SDI	12.48	9.88	9.89	9.65	86.22	87.66	11.08	9.33	9.42	9.54	88.04	88.58
World Bank Income Level
World Bank High Income	3.97	2.52	10.74	7.04	90.45	93.63	3.29	2.24	11.79	7.49	90.75	93.67
World Bank Upper Middle Income	5.95	3.67	18.12	11.34	85.77	90.90	5.18	3.56	19.23	12.74	86.22	90.43
World Bank Lower Middle Income	12.00	9.61	10.26	7.06	86.28	89.95	10.45	8.79	10.77	7.56	87.58	90.50
World Bank Low Income	10.12	6.70	11.18	11.98	87.17	88.24	9.01	6.40	10.22	11.62	89.02	89.11
Region
Andean Latin America	7.43	5.44	15.01	10.32	84.73	89.06	6.79	5.25	14.84	10.52	85.66	89.04
Australasia	6.33	4.55	5.43	3.54	92.09	94.54	5.47	4.12	6.08	3.71	92.70	94.91
Caribbean	9.09	5.86	8.51	5.62	89.06	92.73	8.14	5.56	8.00	5.14	90.48	93.56
Central Asia	4.05	2.69	12.47	7.63	90.56	93.85	3.46	2.47	11.83	6.65	91.83	94.79
Central Europe	3.24	1.85	27.67	18.73	82.06	87.15	2.93	1.75	26.44	17.68	84.00	88.35
Central Latin America	8.26	6.05	17.21	9.33	84.85	90.42	7.20	5.46	16.85	9.65	86.25	90.81
Central Sub-Saharan Africa	7.20	4.98	5.24	4.54	92.58	94.38	6.65	4.77	5.04	4.54	93.42	94.70
East Asia	7.82	5.32	30.72	23.72	77.28	82.65	7.04	5.10	33.79	26.36	76.74	81.56
Eastern Europe	1.51	0.77	12.28	5.87	92.78	96.34	1.33	0.71	12.49	6.44	92.97	96.25
Eastern Sub-Saharan Africa	9.04	4.98	12.73	16.46	86.91	86.67	7.55	4.42	11.25	16.37	89.39	87.63
High-income Asia Pacific	2.23	1.67	16.88	14.16	87.41	88.96	1.85	1.46	17.33	14.52	87.96	89.18
High-income North America	4.97	2.78	10.37	5.99	89.25	94.06	4.06	2.45	11.89	6.70	89.21	93.89
North Africa and Middle East	9.82	6.23	4.01	2.06	91.59	95.04	8.91	5.89	4.34	2.25	92.36	95.34
Oceania	3.07	1.52	19.48	14.40	83.29	88.83	2.36	1.21	16.46	12.39	86.64	90.72
South Asia	14.44	12.06	10.23	6.90	84.35	88.23	13.07	11.37	11.33	7.77	85.24	88.55
Southeast Asia	4.39	2.53	19.09	13.78	86.73	90.75	4.06	2.46	18.99	14.40	87.45	90.72
Southern Latin America	3.18	1.91	12.40	9.38	90.07	92.61	2.87	1.76	12.60	9.38	90.54	92.94
Southern Sub-Saharan Africa	5.46	2.64	5.12	4.84	93.88	95.77	4.71	2.50	5.84	5.38	94.38	95.83
Tropical Latin America	5.04	3.94	12.47	8.65	89.70	92.19	4.32	3.55	12.37	8.53	90.68	92.73
Western Europe	3.82	2.63	8.19	4.64	92.47	95.32	3.18	2.34	8.86	4.87	92.91	95.47
Western Sub-Saharan Africa	5.72	4.16	7.75	6.68	92.91	93.87	5.38	3.93	7.72	7.01	93.36	94.08

PAF, population attributable fraction; ASDR, age-standardized death rate; ASDALYR, the age-standardized disability-adjusted life years rate; CAVD, calcific aortic valve disease.

### Relevant factors

**Figure 6:**
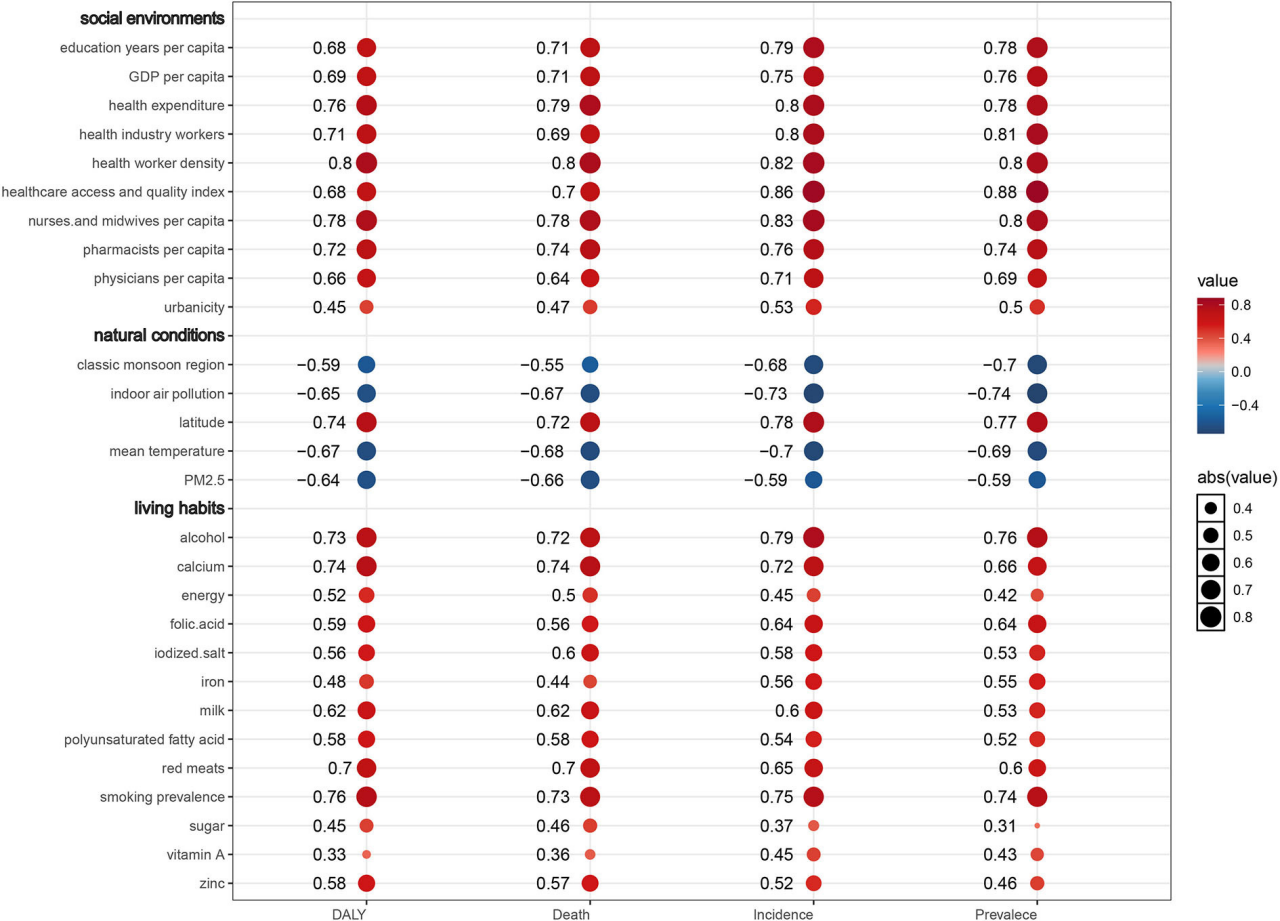
Covariates of CAVD. CAVD, calcific aortic valve disease.

The correlation of exposure factors with ASIR, ASPR, ASDR, and ASDALYR of CAVD were analyzed, which were stratified as social environment, natural conditions, and living habits (Figure 6).

#### Social environment and burden of CAVD

Health worker density (*R* = 0.82, *P* < 0.001), health industry workers (*R* = 0.80, *P* < 0.001), healthcare access and quality index (*R* = 0.86, *P* < 0.001), and nurses and midwives per capita (*R* = 0.83, *P* < 0.001) showed strong positive correlations to ASIR of CAVD. At the same time, education years per capita (*R* = 0.79, *P* < 0.001), Gross Domestic Product (GDP) per capita (*R* = 0.75, *P* < 0.001), health expenditure per capita (*R* = 0.795, *P* < 0.001), pharmacists per capita (*R* = 0.76, *P* < 0.001), physicians per capita (*R* = 0.71, *P* < 0.001), and urbanicity (*R* = 0.53, *P* < 0.001) showed moderately positive correlations toASIR. The relationships between social environment and ASPR, and ASDR as well as ASDALYR were analogous.

#### Natural conditions and burden of CAVD

As for natural environmental factors, classic monsoon region (*R* = −0.68, *P* < 0.001), indoor air pollution (*R* = −0.73, *P* < 0.001), mean temperature (*R* = −0.7, *P* < 0.001), and outdoor PM2.5 (*R* = −0.59, *P* < 0.001) were negatively correlated with ASIR of CAVD. However, latitude (*R* = 0.73, *P* < 0.001) had a moderately positive correlation. Similar results were found in the analysis of ASPR, ASDR, and ASDALYR.

#### Living habits and burden of CAVD

All living habit factors with significant correlations manifested positive correlations. However, these were moderately relevant factors not strong ones: alcohol (*R* = 0.79, *P* < 0.001), smoking prevalence (*R* = 0.75, *P* < 0.001), calcium (*R* = 0.72, *P* < 0.001), red meats (*R* = 0.65, *P* < 0.001), folic acid (*R* = 0.64, *P* < 0.001), milk (*R* = 0.60, *P* < 0.001), and so on. Consistent results were found in the relationships among ASPR, ASDR, ASDALYR, and these lifestyle-related factors.

## Discussion

In this study, we comprehensively reported the recent global burden and trends from 1990 to 2019 according to the GBD 2019 study. Great regional differences were found in the disease distribution of CAVD. High SDI regions had the highest burdens, especially in the elderly population, and still shoed an upwards trend. Although the ASIR and ASPR presented a global upwards trend, ASDR was relatively stable. Even ASDALYR gradually declined. Age was a powerful factor to develop CAVD and males were more likely to suffer from it. High SBP was a major attributable risk to CAVD. In addition, various covariates stratified as social environment, natural conditions, and living habits were found relative to the burden of CAVD.

Our results revealed that the epidemiological distributions of CAVD were markedly different around the world. High SDI regions had the highest burdens of CAVD including incidence, prevalence, deaths, and DALYs. Older age was a strong risk factor for calcific AS (1, 16). Interestingly, the highest age-standardized rates of these four statistical variables were still in high SDI regions. Several research elaborated on the significant associations between cardiovascular risk factors (such as smoking, high SBP, high body mass index, hyperlipemia, etc.) and CAVD (1, 17–20). Widely varied spatial distribution among the global population of these traditional cardiovascular risk factors might partly account for the differences in the geographical distribution of CAVD. In addition, high SDI countries such as the UK and the USA had more sound disease diagnosis and registry systems compared with other regions. Age was an extremely powerful factor in CAVD, no matter in incidence, prevalence, deaths, or DALYs. In this study, we found that CAVD-related deaths and DALYs surged among patients aged over 75 years. Once an estimate told that the hazard of AS increases by 75% for each decade increase in age (20). As life expectancy remains increasing, inevitably, the burden of AS is anticipated to surge (21). Globally, male individuals were more likely to suffer from CAVD than female individuals with higher prevalence cases, death cases, DALYs, and corresponding age-standardized rates (6). Moreover, female individuals tended to present more fibrotic remodeling and less calcification than male individuals (22). Meanwhile, female individuals showed a more advanced age of onset of the disease (21). Studies focused on sex-specific features implied that the progression, pathophysiology, and hemodynamic severity of aortic valve calcification varied in males and females (23–25). Therefore, in future, it is of great importance to consider sex-related difference in the clinical practice of diagnosis, treatment, and prognosis (23).

We found out that the ASIR and ASPR of CAVD displayed a pretty great uptrend from 1990 to 2019 globally. Despite that cardiac catheterization is the gold standard of diagnosis for CAVD, echocardiography examination, which provide wealthy and immediate information on cardiac structure and function, has been widely used in clinical diagnosis (26). With this convenient and relatively low-cost technique, more patients were screened especially in high-middle SDI and middle SDI regions. While the ASIR and ASPR in high SDI regions remained increasing, the growth rate had given way to high-middle SDI and middle SDI regions such as East Asia and Eastern Europe. Maybe medical services and public health were increasingly valued by the government in these regions. Taking China, for instance, transthoracic echocardiography was prevalently used in diagnosing and evaluating the severity of valvular heart disease (27). Thus, more cases were diagnosed and registered in these regions. However, the ASDR was relatively stable during the past 30 years, particularly in high SDI regions. And more encouragingly, globally, the ASDALYR of CAVD presented a gradual downward trend during this period, particularly in high SDI regions where the prevalence and ASPR were the highest in 2019 at the socio-demographic factor level (11), which sent a positive signal to control and ameliorate the confused and intractable disease. For the past few years, most individuals were diagnosed as asymptomatic patients, and it would take several years for the onset of symptoms during the regular follow-up. Transcatheter aortic valve replacement (TAVR), first performed on humans in 2002, turned out to be an epochmaking intervention for CAVD treatment recently, especially for those patients with advanced age and lack of surgery tolerance (28). In the 2020 ACC/AHA Guideline, TAVR was recommended in preference to surgery among patients over 80 years of age while surgical aortic valve replacement (SAVR) was first recommended among individuals aged younger than 65 years (26). Thus, since CAVD is highly correlated with age particularly among individuals over 70 years, emerging TAVR brought good news to these patients. In research, enrolling 246 consecutive AS patients who underwent TAVR, obvious hemodynamics improvement of the aortic valve and cardiac reverse remodeling were observed after a 3-year follow-up (29). Therefore, TAVR significantly improved the living qualities and reduced disabilities caused by CAVD, especially for patients with advanced ages.

We analyzed the three attributable risk factors (lead exposure, high SBP, and diet high in sodium) available in GBD 2019 of CAVD. We found out that high SBP remained the predominating attributable risk factor to CAVD, which is consistent with previous studies (20, 30, 31). The CANHEART Aortic Stenosis study, a large observational cohort study of 1.12 million individuals older than 65 years, using a population-based longitudinal approach revealed an independent and dose-response association between well-known cardiovascular risk factors such as hypertension, diabetes, as well as dyslipidemia and the risk of developing severe AS (20). Moreover, due to a higher prevalence in elder people and a higher HR, hypertension presented the highest attributed risk to AS. And these three risk factors combinedly accounted for approximately one-third (34.4%) of the attributable risk for AS (20). The PROGRESSA study indicated that high SBP had a significant relationship with faster progression of aortic valve calcification (AVC) (31). A novel finding was that high SBP could disturb the evaluation of the hemodynamic severity of AS and thus may cover up its progression (31). Systolic hypertension may increase the bending stress on the valve leaflets during the ventricular ejection stage (31, 32). Meanwhile, it may cause faster and more abrupt closing of the aortic valve leading to the increase of tensile stress in early diastole (31, 33). All these mechanical stresses may damage endothelial cells of valve leaflets resulting in inflammatory activation and the infiltration of lipids (1, 17, 21, 31). Furthermore, a study consisting of 737 elder patients manifested that after adjustment for atherosclerosis-related risk factors, only ambulatory mean diastolic blood pressure (DBP) displayed a significant association with advanced AVC independently, which suggests that DBP may play a more important role in the early stage of CAVD than SBP (19). Fortunately, all these three attributable risks for CAVD displayed a downward trend in general, especially for high SBP. A systematic analysis from 90 countries showed that the increase in awareness, treatment, and control in high-income countries was substantial while it was less in low- and middle-income countries (34). Thus, ambulatory blood pressure monitoring and controlling, no matter whether SBP or DBP, could play a great important role in preventing CAVD.

Although the exact mechanism and pathophysiology of CAVD are still not clear, the existing research demonstrates that it is a series of the chronic progression of endothelium damage, lipid infiltration and oxidation, chronic inflammation, and finally fibrosis and biomineralization (1, 6, 17, 21, 35, 36). In this study, we found some relevant exposure factors related to the burden of CAVD, which may provide some ideas and orientations for prevention. Avoiding risk factors and reducing exposure are important for prevention. Our results revealed that alcohol, calcium, salt, milk, red meats, and smoking had a positive relationship with the burden of aortic aneurysm. In contrast, the classic monsoon region and temperature were negatively related to that. Alcohol consumption was positively associated with cardiovascular events and all-cause mortality presenting a curvilinear dose-response (37, 38). The influx of Ca^2+^ through CaV1.2 (an L-type voltage-gated Ca2+ channel) was proved to promote aortic valve calcification (39). A case-control study involving 132 individuals revealed that a small increase in calcium level would lead to a significant rise in the risk of AS (40, 41). And smoking, a conventional cardiovascular risk factor, was linked to CAVD (17, 42). Although there were no direct evidence to confirm the association between salt, temperature, and the development of CAVD, they might exert as intermediate variables. For example, salt was a risk factor for hypertension and high ambient temperature may serve as a protective factor (43–45). Therefore, avoiding smoking, lowering alcohol consumption, limiting calcium and salt intake, controlling blood pressure, and increasing the room temperature appropriately could be cost-effective measures to reduce the risk of developing AS and CAVD.

There are some limitations in this study. First, all of our analysis relied on the quality of the disease registry data. Inevitably, data on CAVD were sparse. Fortunately, several statistical approaches were adopted to reduce the influence. Second, GBD is a descriptive study that lacks causal argument. So, further analytical and experimental studies are needed to confirm these results. Third, potentially confounding factors may interfere with the calculation of correlation coefficients.

## Conclusions

CAVD displays widely varied spatial distribution around the world and high SDI regions have the highest burdens. Although the ASIR and ASPR present an upward trend globally, ASDR is relatively stable. Even ASDALYR gradually declines. Age is a powerful factor to develop CAVD and male individuals are more likely to suffer from it. Blood pressure control, both SBP and DBP, should be paid high attention to. In addition, avoiding smoking, reducing alcohol consumption, limiting calcium and salt intake, and rising the room temperature appropriately might decrease the burden of CAVD. Our findings may aid in providing information on comprehensive prevention measures in response to the challenges of valvular heart disease.

## Data availability statement

The original contributions presented in the study are included in the article/Supplementary material, further inquiries can be directed to the corresponding author.

## Author contributions

JY and ZW designed the study, retrieved the data, analyzed the data, and performed the data visualization. JY, QB, SL, and ZY interpreted the results and wrote the manuscript. YY revised the manuscript. XX directed the study and checked and approved the manuscript. All authors consented to submit the manuscript.

## Funding

XX was supported by the National Health Commission, Key Program of Science and Technology of Medical and Health of Zhejiang Province (WKJ-ZJ-2028).

## Conflict of interest

The authors declare that the research was conducted in the absence of any commercial or financial relationships that could be construed as a potential conflict of interest.

## Publisher's note

All claims expressed in this article are solely those of the authors and do not necessarily represent those of their affiliated organizations, or those of the publisher, the editors and the reviewers. Any product that may be evaluated in this article, or claim that may be made by its manufacturer, is not guaranteed or endorsed by the publisher.
