# Supplementary material for: Global burden of calcific aortic valve disease and attributable risk factors from 1990 to 2019
Source: Front Cardiovasc Med. 2022 Nov 23;9:1003233. doi: 10.3389/fcvm.2022.1003233 (PMC9727398; doi:10.3389/fcvm.2022.1003233)
Supplement: Supplementary file 1 [file Table_1.DOCX]

**TABLE 1 |** The incidence of CAVD in 1990/2019

**TABLE 2 |** The prevalence of CAVD in 1990/2019

**TABLE 3 |** The death of CAVD in 1990/2019

**TABLE 4 |** The DALYs of CAVD in 1990/2019

**TABLE 5 |** The temporal trends of ASIR, ASPR, ASDR, ASDALYR from 1990 to 2019 of CAVD

**TABLE 6 |** Attributable risks of ASDR in CAVD

**TABLE 7 |** Attributable risks of ASDALYR in CAVD

**TABLE 8 |** PAF of attributable risks of ASDR and ASDALYR to CAVD in 2019

**FIGURE 1 |** ASIR and its trend of CAVD. (A) ASIR in 31 regions from 1990 to 2019. (B) ASIR stratified by age in the globe and 5 SDI regions in 2019. (C) ASIR in 204 countries and territories in 2019. (D) EAPC of ASIR in 31 regions from 1990 to 2019. (E) EAPC of ASIR in 204 countries and territories from 1990 to 2019. CAVD, calcific aortic valve disease; ASIR, the age-standardized incidence rate; SDI, socio-demographic index; EAPC, estimated annual percentage change

**FIGURE 2 |** ASPR and its trend of CAVD. (A) ASPR in 31 regions from 1990 to 2019. (B) ASPR stratified by age in the globe and 5 SDI regions in 2019. (C) ASPR in 204 countries and territories in 2019. (D) EAPC of ASPR in 31 regions from 1990 to 2019. (E) EAPC of ASPR in 204 countries and territories from 1990 to 2019. CAVD, calcific aortic valve disease; ASPR, the age-standardized prevalence rate; SDI, socio-demographic index; EAPC, estimated annual percentage change

**FIGURE 3 |** ASDR and its trend of CAVD. (A) ASDR in 31 regions from 1990 to 2019. (B) ASDR stratified by age in the globe and 5 SDI regions in 2019. (C) ASDR in 204 countries and territories in 2019. (D) EAPC of ASDR in 31 regions from 1990 to 2019. (E) EAPC of ASIR in 204 countries and territories from 1990 to 2019. CAVD, calcific aortic valve disease; ASDR, the age-standardized death rate; SDI, socio-demographic index; EAPC, estimated annual percentage change

**FIGURE 4 |** ASDALYR and its trend of CAVD. (A) ASDALYR in 31 regions from 1990 to 2019. (B) ASDALYR stratified by age in the globe and 5 SDI regions in 2019. (C) ASDALYR in 204 countries and territories in 2019. (D) EAPC of ASDALYR in 31 regions from 1990 to 2019. (E) EAPC of ASDALYR in 204 countries and territories from 1990 to 2019. CAVD, calcific aortic valve disease; ASDALYR, the age-standardized disability-adjusted life years rate; SDI, socio-demographic index; EAPC, estimated annual percentage change

**FIGURE 5 |** Attributable risks of CAVD. (A) EAPC and attributable risks of ASDR in 31 regions. (B) PAF of attributable risks of ASDR in 2019. (C) EAPC and attributable risks of ASDALYR in 31 regions. (D) PAF of attributable risks of ASDALYR in 2019. CAVD, calcific aortic valve disease; EAPC, estimated annual percentage change; ASDR, age-standardized death rate; PAF, population attributable fraction; ASDALYR, the age-standardized disability-adjusted life years rate

**FIGURE 6 |** Covariates of CAVD. CAVD, calcific aortic valve disease
