# Supplementary material for: Global burden of calcific aortic valve disease and attributable risk factors from 1990 to 2019
Source: Front Cardiovasc Med. 2022 Nov 23;9:1003233. doi: 10.3389/fcvm.2022.1003233 (PMC9727398; doi:10.3389/fcvm.2022.1003233)
Supplement: Supplementary file 2 [file Data_Sheet_1.PDF]

Supplementary Table1: The incidence,prevalence,deaths and DALYs (Disability-Adjusted Life Years) of CAVD in 1990 and 2019 in 204 countries and territories.

Measure	Region	Sex	Year	Value(per 100,000)	95%UI(per 100,000)
Incidence	Afghanistan	Both	1990	0.58	(0.50 ,0.68)
Incidence	Albania	Both	1990	2.61	(2.29 ,2.96)
Incidence	Algeria	Both	1990	0.82	(0.73 ,0.93)
Incidence	American Samoa	Both	1990	5.03	(4.08 ,6.10)
Incidence	Andorra	Both	1990	0.73	(0.60 ,0.90)
Incidence	Angola	Both	1990	0.95	(0.85 ,1.06)
Incidence	Antigua	Both	1990	1.46	(1.34 ,1.60)
Incidence	Argentina	Both	1990	2.65	(2.10 ,3.79)
Incidence	Armenia	Both	1990	0.46	(0.38 ,0.56)
Incidence	Australia	Both	1990	4.65	(4.14 ,5.24)
Incidence	Austria	Both	1990	3.53	(3.01 ,4.12)
Incidence	Azerbaijan	Both	1990	0.48	(0.39 ,0.61)
Incidence	Bahamas	Both	1990	1.55	(1.41 ,1.70)
Incidence	Bahrain	Both	1990	3.02	(2.56 ,3.50)
Incidence	Bangladesh	Both	1990	0.82	(0.73 ,0.93)
Incidence	Barbados	Both	1990	1.94	(1.77 ,2.14)
Incidence	Belarus	Both	1990	1.62	(1.29 ,1.99)
Incidence	Belgium	Both	1990	0.62	(0.51 ,0.74)
Incidence	Belize	Both	1990	1.03	(0.91 ,1.16)
Incidence	Benin	Both	1990	0.55	(0.48 ,0.63)
Incidence	Bermuda	Both	1990	6.85	(5.87 ,8.18)
Incidence	Bhutan	Both	1990	0.84	(0.74 ,0.97)
Incidence	Bolivia	Both	1990	0.83	(0.74 ,0.93)
Incidence	Bosnia and Herzegovina	Both	1990	3.40	(2.94 ,3.96)
Incidence	Botswana	Both	1990	0.84	(0.75 ,0.94)
Incidence	Brazil	Both	1990	2.24	(1.92 ,2.60)
Incidence	Brunei Darussalam	Both	1990	5.46	(4.20 ,6.89)
Incidence	Bulgaria	Both	1990	7.71	(6.20 ,9.32)
Incidence	Burkina Faso	Both	1990	0.50	(0.43 ,0.57)
Incidence	Burundi	Both	1990	0.88	(0.79 ,0.98)
Incidence	Cabo Verde	Both	1990	0.52	(0.45 ,0.60)
Incidence	Cambodia	Both	1990	0.26	(0.22 ,0.31)
Incidence	Cameroon	Both	1990	0.78	(0.68 ,0.90)
Incidence	Canada	Both	1990	0.72	(0.60 ,0.85)
Incidence	Central African Republic	Both	1990	0.91	(0.81 ,1.01)
Incidence	Chad	Both	1990	0.47	(0.41 ,0.55)
Incidence	Chile	Both	1990	2.18	(1.96 ,2.40)
Incidence	China	Both	1990	0.34	(0.27 ,0.42)
Incidence	Colombia	Both	1990	1.38	(1.15 ,1.56)
Incidence	Comoros	Both	1990	1.00	(0.90 ,1.11)
Incidence	Cook Islands	Both	1990	1.57	(1.31 ,1.87)
Incidence	Costa Rica	Both	1990	1.56	(1.41 ,1.72)
Incidence	Croatia	Both	1990	4.50	(3.68 ,5.36)
Incidence	Cuba	Both	1990	1.26	(1.15 ,1.39)
Incidence	Cyprus	Both	1990	0.79	(0.66 ,0.95)
Incidence	Czechia	Both	1990	4.27	(3.39 ,5.40)
Incidence	Democratic Republic of the C	Both	1990	0.92	(0.81 ,1.05)
Incidence	Denmark	Both	1990	0.59	(0.49 ,0.72)
Incidence	Djibouti	Both	1990	0.98	(0.88 ,1.09)

Incidence	Dominica	Both	1990	1.56	(1.42 ,1.71)
Incidence	Dominican Republic	Both	1990	0.89	(0.79 ,1.00)
Incidence	Ecuador	Both	1990	0.97	(0.86 ,1.11)
Incidence	Egypt	Both	1990	1.65	(1.32 ,2.02)
Incidence	El Salvador	Both	1990	0.55	(0.46 ,0.67)
Incidence	Equatorial Guinea	Both	1990	0.89	(0.80 ,0.99)
Incidence	Eritrea	Both	1990	0.87	(0.78 ,0.97)
Incidence	Estonia	Both	1990	3.60	(2.97 ,4.31)
Incidence	Ethiopia	Both	1990	0.67	(0.56 ,0.80)
Incidence	Fiji	Both	1990	0.98	(0.82 ,1.19)
Incidence	Finland	Both	1990	3.48	(2.86 ,4.17)
Incidence	France	Both	1990	0.78	(0.65 ,0.93)
Incidence	Gabon	Both	1990	1.19	(1.07 ,1.33)
Incidence	Gambia	Both	1990	0.58	(0.51 ,0.66)
Incidence	Georgia	Both	1990	1.27	(1.03 ,1.53)
Incidence	Germany	Both	1990	0.70	(0.58 ,0.83)
Incidence	Ghana	Both	1990	0.46	(0.40 ,0.54)
Incidence	Greece	Both	1990	0.72	(0.59 ,0.88)
Incidence	Greenland	Both	1990	0.62	(0.52 ,0.75)
Incidence	Grenada	Both	1990	1.47	(1.03 ,1.72)
Incidence	Guam	Both	1990	3.26	(2.76 ,3.86)
Incidence	Guatemala	Both	1990	0.54	(0.46 ,0.64)
Incidence	Guinea	Both	1990	0.51	(0.44 ,0.59)
Incidence	Guinea-Bissau	Both	1990	0.72	(0.62 ,0.82)
Incidence	Guyana	Both	1990	1.92	(1.68 ,2.21)
Incidence	Haiti	Both	1990	1.47	(1.19 ,1.64)
Incidence	Honduras	Both	1990	0.65	(0.57 ,0.74)
Incidence	Hungary	Both	1990	12.39	(10.74 ,14.49)
Incidence	Iceland	Both	1990	0.70	(0.58 ,0.85)
Incidence	India	Both	1990	0.86	(0.72 ,1.02)
Incidence	Indonesia	Both	1990	0.30	(0.24 ,0.36)
Incidence	Iran	Both	1990	0.74	(0.62 ,0.89)
Incidence	Iraq	Both	1990	1.58	(1.30 ,1.93)
Incidence	Ireland	Both	1990	0.74	(0.61 ,0.90)
Incidence	Israel	Both	1990	0.57	(0.47 ,0.69)
Incidence	Italy	Both	1990	5.86	(4.91 ,7.01)
Incidence	Ivory Coast	Both	1990	0.70	(0.62 ,0.78)
Incidence	Jamaica	Both	1990	0.66	(0.56 ,0.78)
Incidence	Japan	Both	1990	17.10	(14.22 ,20.52)
Incidence	Jordan	Both	1990	1.24	(1.03 ,1.64)
Incidence	Kazakhstan	Both	1990	0.95	(0.77 ,1.19)
Incidence	Kenya	Both	1990	0.91	(0.76 ,1.08)
Incidence	Kiribati	Both	1990	0.79	(0.66 ,0.94)
Incidence	Kuwait	Both	1990	4.95	(4.06 ,6.00)
Incidence	Kyrgyzstan	Both	1990	0.38	(0.31 ,0.47)
Incidence	Laos	Both	1990	0.32	(0.28 ,0.37)
Incidence	Latvia	Both	1990	3.60	(3.06 ,4.28)
Incidence	Lebanon	Both	1990	1.02	(0.79 ,1.48)
Incidence	Lesotho	Both	1990	0.86	(0.77 ,0.97)
Incidence	Liberia	Both	1990	0.53	(0.45 ,0.63)
Incidence	Libya	Both	1990	1.73	(1.39 ,2.10)

Incidence	Lithuania	Both	1990	2.55	(2.08 ,3.03)
Incidence	Luxembourg	Both	1990	1.59	(1.31 ,1.95)
Incidence	Macedonia	Both	1990	3.25	(2.64 ,3.94)
Incidence	Madagascar	Both	1990	1.09	(0.98 ,1.20)
Incidence	Malawi	Both	1990	0.75	(0.67 ,0.85)
Incidence	Malaysia	Both	1990	0.49	(0.43 ,0.57)
Incidence	Maldives	Both	1990	0.38	(0.32 ,0.45)
Incidence	Mali	Both	1990	0.50	(0.43 ,0.58)
Incidence	Malta	Both	1990	0.45	(0.37 ,0.55)
Incidence	Marshall Islands	Both	1990	0.93	(0.77 ,1.11)
Incidence	Mauritania	Both	1990	0.69	(0.61 ,0.78)
Incidence	Mauritius	Both	1990	0.49	(0.42 ,0.57)
Incidence	Mexico	Both	1990	1.23	(1.04 ,1.45)
Incidence	Micronesia	Both	1990	1.49	(1.25 ,1.79)
Incidence	Monaco	Both	1990	1.08	(0.87 ,1.37)
Incidence	Mongolia	Both	1990	0.39	(0.32 ,0.47)
Incidence	Montenegro	Both	1990	6.57	(5.20 ,8.16)
Incidence	Morocco	Both	1990	0.64	(0.55 ,0.76)
Incidence	Mozambique	Both	1990	0.81	(0.72 ,0.90)
Incidence	Myanmar	Both	1990	0.28	(0.24 ,0.33)
Incidence	Namibia	Both	1990	1.00	(0.89 ,1.11)
Incidence	Nauru	Both	1990	1.23	(1.04 ,1.46)
Incidence	Nepal	Both	1990	0.71	(0.63 ,0.81)
Incidence	Netherlands	Both	1990	0.62	(0.52 ,0.74)
Incidence	New Zealand	Both	1990	10.11	(8.39 ,11.95)
Incidence	Nicaragua	Both	1990	0.54	(0.45 ,0.63)
Incidence	Niger	Both	1990	0.50	(0.43 ,0.58)
Incidence	Nigeria	Both	1990	0.58	(0.49 ,0.69)
Incidence	Niue	Both	1990	1.09	(0.93 ,1.28)
Incidence	North Korea	Both	1990	0.22	(0.18 ,0.26)
Incidence	Northern Mariana Islands	Both	1990	4.01	(3.29 ,4.92)
Incidence	Norway	Both	1990	1.51	(1.23 ,1.83)
Incidence	Oman	Both	1990	1.14	(1.02 ,1.28)
Incidence	Pakistan	Both	1990	1.40	(1.17 ,1.64)
Incidence	Palau	Both	1990	1.05	(0.84 ,1.29)
Incidence	Palestine	Both	1990	0.72	(0.52 ,0.92)
Incidence	Panama	Both	1990	0.94	(0.80 ,1.10)
Incidence	Papua New Guinea	Both	1990	0.84	(0.70 ,0.99)
Incidence	Paraguay	Both	1990	1.62	(1.48 ,1.76)
Incidence	Peru	Both	1990	0.59	(0.52 ,0.67)
Incidence	Philippines	Both	1990	0.26	(0.21 ,0.33)
Incidence	Poland	Both	1990	6.84	(5.52 ,8.22)
Incidence	Portugal	Both	1990	0.35	(0.28 ,0.43)
Incidence	Puerto Rico	Both	1990	4.77	(4.16 ,5.53)
Incidence	Qatar	Both	1990	9.23	(7.56 ,11.14)
Incidence	Republic of Congo	Both	1990	1.11	(1.00 ,1.23)
Incidence	Republic of Moldova	Both	1990	1.16	(0.96 ,1.37)
Incidence	Romania	Both	1990	7.22	(5.93 ,8.72)
Incidence	Russia	Both	1990	2.89	(2.32 ,3.53)
Incidence	Rwanda	Both	1990	1.03	(0.91 ,1.15)
Incidence	Saint Kitts	Both	1990	1.39	(1.26 ,1.52)

Incidence	Saint Lucia	Both	1990	1.66	(1.46 ,1.82)
Incidence	Saint Vincent and the Grenac	Both	1990	1.34	(1.15 ,1.55)
Incidence	Samoa	Both	1990	2.25	(1.89 ,2.69)
Incidence	San Marino	Both	1990	0.67	(0.53 ,0.82)
Incidence	Sao Tome and Principe	Both	1990	0.53	(0.46 ,0.62)
Incidence	Saudi Arabia	Both	1990	1.02	(0.80 ,1.39)
Incidence	Senegal	Both	1990	0.50	(0.43 ,0.58)
Incidence	Serbia	Both	1990	4.27	(3.76 ,4.85)
Incidence	Seychelles	Both	1990	0.69	(0.60 ,0.80)
Incidence	Sierra Leone	Both	1990	0.54	(0.48 ,0.62)
Incidence	Singapore	Both	1990	7.02	(5.85 ,8.49)
Incidence	Slovakia	Both	1990	2.78	(2.26 ,3.43)
Incidence	Slovenia	Both	1990	8.23	(6.64 ,10.17)
Incidence	Solomon Islands	Both	1990	0.96	(0.79 ,1.15)
Incidence	Somalia	Both	1990	0.86	(0.76 ,0.96)
Incidence	South Africa	Both	1990	1.84	(1.56 ,2.19)
Incidence	South Korea	Both	1990	6.74	(5.58 ,8.20)
Incidence	South Sudan	Both	1990	0.92	(0.82 ,1.03)
Incidence	Spain	Both	1990	0.66	(0.55 ,0.80)
Incidence	Sri Lanka	Both	1990	0.59	(0.49 ,0.68)
Incidence	Sudan	Both	1990	0.65	(0.57 ,0.75)
Incidence	Suriname	Both	1990	1.17	(1.05 ,1.30)
Incidence	Swaziland	Both	1990	1.27	(1.03 ,1.68)
Incidence	Sweden	Both	1990	3.04	(2.46 ,3.76)
Incidence	Switzerland	Both	1990	1.16	(0.95 ,1.48)
Incidence	Syrian	Both	1990	1.72	(1.47 ,1.93)
Incidence	Taiwan	Both	1990	0.55	(0.45 ,0.66)
Incidence	Tajikistan	Both	1990	0.28	(0.23 ,0.35)
Incidence	Tanzania	Both	1990	0.86	(0.77 ,0.97)
Incidence	Thailand	Both	1990	0.35	(0.30 ,0.40)
Incidence	Timor-Leste	Both	1990	0.28	(0.23 ,0.33)
Incidence	Togo	Both	1990	0.58	(0.51 ,0.66)
Incidence	Tokelau	Both	1990	0.77	(0.65 ,0.90)
Incidence	Tonga	Both	1990	1.60	(1.35 ,1.90)
Incidence	Trinidad	Both	1990	1.29	(1.11 ,1.54)
Incidence	Tunisia	Both	1990	0.80	(0.70 ,0.93)
Incidence	Turkey	Both	1990	1.40	(1.26 ,1.55)
Incidence	Turkmenistan	Both	1990	0.41	(0.33 ,0.50)
Incidence	Tuvalu	Both	1990	0.68	(0.58 ,0.79)
Incidence	Uganda	Both	1990	0.75	(0.66 ,0.85)
Incidence	UK	Both	1990	9.25	(7.50 ,11.40)
Incidence	Ukraine	Both	1990	3.26	(2.61 ,3.92)
Incidence	United Arab Emirates	Both	1990	2.92	(2.35 ,3.56)
Incidence	Uruguay	Both	1990	2.79	(2.17 ,3.59)
Incidence	USA	Both	1990	15.77	(12.89 ,19.16)
Incidence	Uzbekistan	Both	1990	0.38	(0.31 ,0.47)
Incidence	Vanuatu	Both	1990	1.10	(0.91 ,1.31)
Incidence	Venezuela	Both	1990	1.26	(1.14 ,1.38)
Incidence	Vietnam	Both	1990	0.29	(0.25 ,0.34)
Incidence	Virgin Islands	Both	1990	3.82	(3.41 ,4.33)
Incidence	Yemen	Both	1990	0.65	(0.57 ,0.75)

Incidence	Zambia	Both	1990	0.83	(0.73 ,0.93)
Incidence	Zimbabwe	Both	1990	0.87	(0.77 ,0.97)
Prevalence	Afghanistan	Both	1990	0.78	(0.60 ,1.02)
Prevalence	Albania	Both	1990	31.31	(26.06 ,37.57)
Prevalence	Algeria	Both	1990	2.92	(2.28 ,3.66)
Prevalence	American Samoa	Both	1990	45.20	(35.49 ,57.11)
Prevalence	Andorra	Both	1990	7.18	(5.66 ,9.14)
Prevalence	Angola	Both	1990	0.91	(0.72 ,1.13)
Prevalence	Antigua	Both	1990	6.40	(5.23 ,7.74)
Prevalence	Argentina	Both	1990	10.96	(8.53 ,14.45)
Prevalence	Armenia	Both	1990	5.05	(3.89 ,6.40)
Prevalence	Australia	Both	1990	33.21	(27.60 ,39.89)
Prevalence	Austria	Both	1990	52.03	(44.12 ,61.23)
Prevalence	Azerbaijan	Both	1990	4.86	(3.63 ,6.28)
Prevalence	Bahamas	Both	1990	8.71	(7.08 ,10.42)
Prevalence	Bahrain	Both	1990	19.08	(14.93 ,23.99)
Prevalence	Bangladesh	Both	1990	1.09	(0.85 ,1.40)
Prevalence	Barbados	Both	1990	12.59	(10.35 ,15.00)
Prevalence	Belarus	Both	1990	23.35	(18.40 ,28.59)
Prevalence	Belgium	Both	1990	3.35	(2.62 ,4.33)
Prevalence	Belize	Both	1990	5.17	(4.13 ,6.34)
Prevalence	Benin	Both	1990	1.08	(0.85 ,1.34)
Prevalence	Bermuda	Both	1990	53.42	(42.76 ,65.48)
Prevalence	Bhutan	Both	1990	1.58	(1.22 ,2.03)
Prevalence	Bolivia	Both	1990	2.49	(2.00 ,3.06)
Prevalence	Bosnia and Herzegovina	Both	1990	40.23	(32.33 ,48.85)
Prevalence	Botswana	Both	1990	1.58	(1.26 ,1.96)
Prevalence	Brazil	Both	1990	7.85	(6.26 ,9.62)
Prevalence	Brunei Darussalam	Both	1990	62.64	(48.90 ,79.56)
Prevalence	Bulgaria	Both	1990	124.24	(98.96 ,152.51)
Prevalence	Burkina Faso	Both	1990	0.89	(0.70 ,1.13)
Prevalence	Burundi	Both	1990	0.83	(0.67 ,1.03)
Prevalence	Cabo Verde	Both	1990	1.74	(1.36 ,2.13)
Prevalence	Cambodia	Both	1990	0.37	(0.28 ,0.50)
Prevalence	Cameroon	Both	1990	2.13	(1.64 ,2.72)
Prevalence	Canada	Both	1990	5.86	(4.64 ,7.34)
Prevalence	Central African Republic	Both	1990	0.76	(0.60 ,0.94)
Prevalence	Chad	Both	1990	0.78	(0.61 ,0.99)
Prevalence	Chile	Both	1990	14.25	(11.79 ,17.10)
Prevalence	China	Both	1990	2.12	(1.58 ,2.76)
Prevalence	Colombia	Both	1990	7.02	(5.43 ,8.66)
Prevalence	Comoros	Both	1990	1.30	(1.02 ,1.61)
Prevalence	Cook Islands	Both	1990	10.90	(8.57 ,13.69)
Prevalence	Costa Rica	Both	1990	10.84	(8.98 ,13.04)
Prevalence	Croatia	Both	1990	68.76	(55.68 ,82.56)
Prevalence	Cuba	Both	1990	7.47	(6.16 ,8.98)
Prevalence	Cyprus	Both	1990	4.93	(3.85 ,6.17)
Prevalence	Czechia	Both	1990	65.24	(51.59 ,81.37)
Prevalence	Democratic Republic of the C	Both	1990	0.97	(0.77 ,1.24)
Prevalence	Denmark	Both	1990	4.83	(3.71 ,6.18)
Prevalence	Djibouti	Both	1990	1.22	(0.97 ,1.52)

Prevalence	Dominica	Both	1990	7.06	(5.81 ,8.49)
Prevalence	Dominican Republic	Both	1990	3.35	(2.69 ,4.14)
Prevalence	Ecuador	Both	1990	6.48	(5.17 ,8.00)
Prevalence	Egypt	Both	1990	7.14	(5.30 ,9.32)
Prevalence	El Salvador	Both	1990	2.98	(2.31 ,3.76)
Prevalence	Equatorial Guinea	Both	1990	0.84	(0.66 ,1.06)
Prevalence	Eritrea	Both	1990	0.75	(0.60 ,0.91)
Prevalence	Estonia	Both	1990	51.27	(41.94 ,61.85)
Prevalence	Ethiopia	Both	1990	0.68	(0.52 ,0.86)
Prevalence	Fiji	Both	1990	3.96	(3.09 ,5.19)
Prevalence	Finland	Both	1990	40.11	(33.20 ,48.45)
Prevalence	France	Both	1990	5.80	(4.60 ,7.32)
Prevalence	Gabon	Both	1990	1.90	(1.49 ,2.38)
Prevalence	Gambia	Both	1990	1.21	(0.96 ,1.54)
Prevalence	Georgia	Both	1990	17.93	(14.11 ,21.82)
Prevalence	Germany	Both	1990	5.71	(4.50 ,7.16)
Prevalence	Ghana	Both	1990	0.95	(0.73 ,1.22)
Prevalence	Greece	Both	1990	6.50	(5.10 ,8.34)
Prevalence	Greenland	Both	1990	3.79	(3.02 ,4.71)
Prevalence	Grenada	Both	1990	4.70	(3.45 ,5.82)
Prevalence	Guam	Both	1990	26.50	(21.68 ,32.57)
Prevalence	Guatemala	Both	1990	1.96	(1.49 ,2.50)
Prevalence	Guinea	Both	1990	0.87	(0.68 ,1.12)
Prevalence	Guinea-Bissau	Both	1990	1.04	(0.80 ,1.31)
Prevalence	Guyana	Both	1990	5.08	(4.06 ,6.25)
Prevalence	Haiti	Both	1990	2.51	(1.95 ,3.08)
Prevalence	Honduras	Both	1990	2.73	(2.14 ,3.40)
Prevalence	Hungary	Both	1990	186.85	(157.65 ,219.63)
Prevalence	Iceland	Both	1990	6.38	(5.01 ,8.12)
Prevalence	India	Both	1990	1.21	(0.93 ,1.51)
Prevalence	Indonesia	Both	1990	0.55	(0.41 ,0.73)
Prevalence	Iran	Both	1990	3.54	(2.74 ,4.47)
Prevalence	Iraq	Both	1990	8.13	(6.06 ,10.68)
Prevalence	Ireland	Both	1990	6.30	(4.89 ,8.06)
Prevalence	Israel	Both	1990	3.87	(2.95 ,4.91)
Prevalence	Italy	Both	1990	84.07	(68.60 ,101.04)
Prevalence	Ivory Coast	Both	1990	1.37	(1.06 ,1.71)
Prevalence	Jamaica	Both	1990	3.89	(3.07 ,4.86)
Prevalence	Japan	Both	1990	261.58	(214.61 ,314.48)
Prevalence	Jordan	Both	1990	6.34	(4.74 ,8.73)
Prevalence	Kazakhstan	Both	1990	11.96	(9.20 ,15.25)
Prevalence	Kenya	Both	1990	1.32	(1.03 ,1.70)
Prevalence	Kiribati	Both	1990	2.08	(1.64 ,2.71)
Prevalence	Kuwait	Both	1990	59.49	(46.50 ,75.12)
Prevalence	Kyrgyzstan	Both	1990	3.75	(2.85 ,4.83)
Prevalence	Laos	Both	1990	0.41	(0.31 ,0.53)
Prevalence	Latvia	Both	1990	53.05	(44.04 ,63.93)
Prevalence	Lebanon	Both	1990	6.17	(4.49 ,8.69)
Prevalence	Lesotho	Both	1990	1.32	(1.06 ,1.65)
Prevalence	Liberia	Both	1990	1.16	(0.88 ,1.49)
Prevalence	Libya	Both	1990	10.87	(8.23 ,13.90)

Prevalence	Lithuania	Both	1990	38.33	(30.69 ,46.11)
Prevalence	Luxembourg	Both	1990	15.33	(12.10 ,19.62)
Prevalence	Macedonia	Both	1990	46.36	(37.01 ,56.91)
Prevalence	Madagascar	Both	1990	1.30	(1.05 ,1.57)
Prevalence	Malawi	Both	1990	0.85	(0.65 ,1.08)
Prevalence	Malaysia	Both	1990	1.48	(1.11 ,1.91)
Prevalence	Maldives	Both	1990	0.90	(0.68 ,1.16)
Prevalence	Mali	Both	1990	0.87	(0.68 ,1.11)
Prevalence	Malta	Both	1990	3.48	(2.63 ,4.49)
Prevalence	Marshall Islands	Both	1990	2.51	(1.99 ,3.24)
Prevalence	Mauritania	Both	1990	1.86	(1.45 ,2.31)
Prevalence	Mauritius	Both	1990	1.68	(1.28 ,2.16)
Prevalence	Mexico	Both	1990	9.96	(7.87 ,12.20)
Prevalence	Micronesia	Both	1990	5.13	(3.93 ,6.61)
Prevalence	Monaco	Both	1990	12.96	(10.11 ,17.05)
Prevalence	Mongolia	Both	1990	2.73	(2.08 ,3.54)
Prevalence	Montenegro	Both	1990	108.71	(86.03 ,134.34)
Prevalence	Morocco	Both	1990	1.83	(1.39 ,2.32)
Prevalence	Mozambique	Both	1990	0.73	(0.57 ,0.92)
Prevalence	Myanmar	Both	1990	0.40	(0.30 ,0.54)
Prevalence	Namibia	Both	1990	1.56	(1.25 ,1.92)
Prevalence	Nauru	Both	1990	5.56	(4.36 ,7.24)
Prevalence	Nepal	Both	1990	0.94	(0.73 ,1.21)
Prevalence	Netherlands	Both	1990	3.66	(2.86 ,4.62)
Prevalence	New Zealand	Both	1990	84.34	(68.75 ,104.19)
Prevalence	Nicaragua	Both	1990	3.12	(2.46 ,3.89)
Prevalence	Niger	Both	1990	0.85	(0.65 ,1.08)
Prevalence	Nigeria	Both	1990	1.40	(1.08 ,1.76)
Prevalence	Niue	Both	1990	5.82	(4.59 ,7.26)
Prevalence	North Korea	Both	1990	0.91	(0.69 ,1.19)
Prevalence	Northern Mariana Islands	Both	1990	34.32	(27.14 ,43.30)
Prevalence	Norway	Both	1990	11.08	(8.81 ,14.10)
Prevalence	Oman	Both	1990	3.69	(2.90 ,4.55)
Prevalence	Pakistan	Both	1990	2.53	(1.98 ,3.23)
Prevalence	Palau	Both	1990	6.56	(4.98 ,8.42)
Prevalence	Palestine	Both	1990	3.15	(2.31 ,4.11)
Prevalence	Panama	Both	1990	5.44	(4.29 ,6.82)
Prevalence	Papua New Guinea	Both	1990	2.03	(1.59 ,2.61)
Prevalence	Paraguay	Both	1990	5.40	(4.40 ,6.57)
Prevalence	Peru	Both	1990	2.91	(2.30 ,3.64)
Prevalence	Philippines	Both	1990	0.60	(0.44 ,0.82)
Prevalence	Poland	Both	1990	113.21	(89.83 ,135.96)
Prevalence	Portugal	Both	1990	2.15	(1.63 ,2.86)
Prevalence	Puerto Rico	Both	1990	53.47	(43.26 ,66.24)
Prevalence	Qatar	Both	1990	108.48	(83.11 ,138.80)
Prevalence	Republic of Congo	Both	1990	1.42	(1.13 ,1.77)
Prevalence	Republic of Moldova	Both	1990	15.88	(12.78 ,19.14)
Prevalence	Romania	Both	1990	117.25	(94.72 ,142.48)
Prevalence	Russia	Both	1990	42.95	(34.28 ,53.09)
Prevalence	Rwanda	Both	1990	1.00	(0.80 ,1.24)
Prevalence	Saint Kitts	Both	1990	6.13	(5.00 ,7.47)

Prevalence	Saint Lucia	Both	1990	5.89	(4.84 ,7.13)
Prevalence	Saint Vincent and the Grenac	Both	1990	4.95	(3.95 ,6.16)
Prevalence	Samoa	Both	1990	13.31	(10.55 ,16.77)
Prevalence	San Marino	Both	1990	6.38	(4.87 ,8.26)
Prevalence	Sao Tome and Principe	Both	1990	1.48	(1.13 ,1.87)
Prevalence	Saudi Arabia	Both	1990	4.61	(3.32 ,6.39)
Prevalence	Senegal	Both	1990	1.12	(0.88 ,1.42)
Prevalence	Serbia	Both	1990	57.34	(48.14 ,67.60)
Prevalence	Seychelles	Both	1990	2.79	(2.10 ,3.60)
Prevalence	Sierra Leone	Both	1990	1.04	(0.81 ,1.31)
Prevalence	Singapore	Both	1990	98.11	(80.67 ,120.32)
Prevalence	Slovakia	Both	1990	40.65	(32.66 ,50.46)
Prevalence	Slovenia	Both	1990	110.19	(87.57 ,136.18)
Prevalence	Solomon Islands	Both	1990	2.37	(1.84 ,3.10)
Prevalence	Somalia	Both	1990	0.78	(0.62 ,0.98)
Prevalence	South Africa	Both	1990	10.20	(7.92 ,12.73)
Prevalence	South Korea	Both	1990	92.66	(75.60 ,111.97)
Prevalence	South Sudan	Both	1990	1.14	(0.88 ,1.45)
Prevalence	Spain	Both	1990	6.14	(4.83 ,7.70)
Prevalence	Sri Lanka	Both	1990	1.61	(1.22 ,2.06)
Prevalence	Sudan	Both	1990	1.31	(1.01 ,1.68)
Prevalence	Suriname	Both	1990	4.60	(3.70 ,5.57)
Prevalence	Swaziland	Both	1990	3.66	(2.59 ,5.11)
Prevalence	Sweden	Both	1990	35.49	(28.84 ,44.74)
Prevalence	Switzerland	Both	1990	12.01	(9.47 ,15.65)
Prevalence	Syrian	Both	1990	6.71	(5.29 ,8.26)
Prevalence	Taiwan	Both	1990	2.31	(1.75 ,2.98)
Prevalence	Tajikistan	Both	1990	2.40	(1.79 ,3.16)
Prevalence	Tanzania	Both	1990	1.06	(0.81 ,1.33)
Prevalence	Thailand	Both	1990	0.97	(0.74 ,1.26)
Prevalence	Timor-Leste	Both	1990	0.42	(0.31 ,0.56)
Prevalence	Togo	Both	1990	1.13	(0.89 ,1.40)
Prevalence	Tokelau	Both	1990	2.77	(2.20 ,3.49)
Prevalence	Tonga	Both	1990	8.28	(6.50 ,10.65)
Prevalence	Trinidad	Both	1990	6.83	(5.36 ,8.54)
Prevalence	Tunisia	Both	1990	4.04	(3.17 ,5.07)
Prevalence	Turkey	Both	1990	4.96	(3.95 ,6.04)
Prevalence	Turkmenistan	Both	1990	3.78	(2.85 ,4.90)
Prevalence	Tuvalu	Both	1990	2.06	(1.61 ,2.60)
Prevalence	Uganda	Both	1990	0.83	(0.64 ,1.05)
Prevalence	UK	Both	1990	118.65	(94.51 ,149.28)
Prevalence	Ukraine	Both	1990	49.15	(39.26 ,59.28)
Prevalence	United Arab Emirates	Both	1990	18.86	(14.42 ,24.46)
Prevalence	Uruguay	Both	1990	12.71	(9.88 ,16.44)
Prevalence	USA	Both	1990	210.23	(171.24 ,256.04)
Prevalence	Uzbekistan	Both	1990	3.65	(2.74 ,4.73)
Prevalence	Vanuatu	Both	1990	3.01	(2.35 ,3.90)
Prevalence	Venezuela	Both	1990	6.51	(5.29 ,7.84)
Prevalence	Vietnam	Both	1990	0.63	(0.48 ,0.83)
Prevalence	Virgin Islands	Both	1990	32.95	(27.00 ,40.62)
Prevalence	Yemen	Both	1990	1.27	(1.00 ,1.61)

Prevalence	Zambia	Both	1990	0.91	(0.70 ,1.17)
Prevalence	Zimbabwe	Both	1990	1.77	(1.39 ,2.21)
Deaths	Afghanistan	Both	1990	0.89	(0.37 ,1.42)
Deaths	Albania	Both	1990	0.45	(0.32 ,0.70)
Deaths	Algeria	Both	1990	1.18	(0.89 ,1.56)
Deaths	American Samoa	Both	1990	1.10	(0.84 ,1.41)
Deaths	Andorra	Both	1990	2.02	(1.40 ,2.80)
Deaths	Angola	Both	1990	0.90	(0.54 ,1.39)
Deaths	Antigua	Both	1990	0.85	(0.71 ,1.00)
Deaths	Argentina	Both	1990	3.92	(3.39 ,4.44)
Deaths	Armenia	Both	1990	0.19	(0.16 ,0.22)
Deaths	Australia	Both	1990	3.29	(2.89 ,3.66)
Deaths	Austria	Both	1990	2.84	(2.34 ,3.22)
Deaths	Azerbaijan	Both	1990	0.14	(0.11 ,0.17)
Deaths	Bahamas	Both	1990	0.82	(0.68 ,0.94)
Deaths	Bahrain	Both	1990	1.47	(1.16 ,1.86)
Deaths	Bangladesh	Both	1990	0.73	(0.43 ,0.99)
Deaths	Barbados	Both	1990	1.00	(0.86 ,1.14)
Deaths	Belarus	Both	1990	0.14	(0.10 ,0.18)
Deaths	Belgium	Both	1990	4.24	(3.59 ,5.22)
Deaths	Belize	Both	1990	0.42	(0.26 ,0.59)
Deaths	Benin	Both	1990	0.58	(0.32 ,0.88)
Deaths	Bermuda	Both	1990	5.29	(4.07 ,6.12)
Deaths	Bhutan	Both	1990	0.65	(0.30 ,1.07)
Deaths	Bolivia	Both	1990	0.82	(0.48 ,1.22)
Deaths	Bosnia and Herzegovina	Both	1990	1.07	(0.74 ,1.39)
Deaths	Botswana	Both	1990	0.86	(0.61 ,1.22)
Deaths	Brazil	Both	1990	1.83	(1.60 ,1.97)
Deaths	Brunei Darussalam	Both	1990	2.02	(1.56 ,2.62)
Deaths	Bulgaria	Both	1990	0.21	(0.16 ,0.33)
Deaths	Burkina Faso	Both	1990	0.58	(0.29 ,0.91)
Deaths	Burundi	Both	1990	1.06	(0.62 ,1.74)
Deaths	Cabo Verde	Both	1990	0.43	(0.33 ,0.53)
Deaths	Cambodia	Both	1990	0.22	(0.14 ,0.36)
Deaths	Cameroon	Both	1990	0.59	(0.33 ,0.91)
Deaths	Canada	Both	1990	2.47	(2.14 ,2.85)
Deaths	Central African Republic	Both	1990	1.10	(0.62 ,1.78)
Deaths	Chad	Both	1990	0.50	(0.27 ,0.76)
Deaths	Chile	Both	1990	1.57	(1.38 ,1.73)
Deaths	China	Both	1990	0.18	(0.11 ,0.26)
Deaths	Colombia	Both	1990	1.48	(1.32 ,1.66)
Deaths	Comoros	Both	1990	0.94	(0.58 ,1.37)
Deaths	Cook Islands	Both	1990	0.49	(0.37 ,0.64)
Deaths	Costa Rica	Both	1990	1.03	(0.87 ,1.17)
Deaths	Croatia	Both	1990	0.57	(0.36 ,1.18)
Deaths	Cuba	Both	1990	0.92	(0.80 ,1.03)
Deaths	Cyprus	Both	1990	10.21	(7.64 ,13.17)
Deaths	Czechia	Both	1990	0.28	(0.18 ,0.50)
Deaths	Democratic Republic of the C	Both	1990	0.95	(0.63 ,1.44)
Deaths	Denmark	Both	1990	2.61	(2.26 ,2.95)
Deaths	Djibouti	Both	1990	0.96	(0.71 ,1.37)

Deaths	Dominica	Both	1990	0.92	(0.73 ,1.10)
Deaths	Dominican Republic	Both	1990	0.76	(0.61 ,0.90)
Deaths	Ecuador	Both	1990	0.59	(0.47 ,0.73)
Deaths	Egypt	Both	1990	1.04	(0.70 ,1.48)
Deaths	El Salvador	Both	1990	0.27	(0.22 ,0.33)
Deaths	Equatorial Guinea	Both	1990	0.95	(0.52 ,1.58)
Deaths	Eritrea	Both	1990	0.90	(0.41 ,1.49)
Deaths	Estonia	Both	1990	0.43	(0.32 ,0.50)
Deaths	Ethiopia	Both	1990	0.85	(0.41 ,1.34)
Deaths	Fiji	Both	1990	0.50	(0.40 ,0.62)
Deaths	Finland	Both	1990	4.08	(3.35 ,4.69)
Deaths	France	Both	1990	4.97	(4.40 ,5.58)
Deaths	Gabon	Both	1990	1.16	(0.81 ,1.67)
Deaths	Gambia	Both	1990	0.53	(0.31 ,0.80)
Deaths	Georgia	Both	1990	0.16	(0.13 ,0.20)
Deaths	Germany	Both	1990	4.63	(3.93 ,5.24)
Deaths	Ghana	Both	1990	0.49	(0.28 ,0.78)
Deaths	Greece	Both	1990	2.44	(2.04 ,2.71)
Deaths	Greenland	Both	1990	4.63	(2.31 ,7.56)
Deaths	Grenada	Both	1990	1.05	(0.70 ,1.35)
Deaths	Guam	Both	1990	1.70	(1.35 ,2.13)
Deaths	Guatemala	Both	1990	0.41	(0.30 ,0.53)
Deaths	Guinea	Both	1990	0.54	(0.30 ,0.84)
Deaths	Guinea-Bissau	Both	1990	0.69	(0.26 ,1.25)
Deaths	Guyana	Both	1990	2.88	(2.37 ,3.42)
Deaths	Haiti	Both	1990	1.36	(0.77 ,1.90)
Deaths	Honduras	Both	1990	0.56	(0.41 ,0.77)
Deaths	Hungary	Both	1990	2.23	(1.79 ,2.47)
Deaths	Iceland	Both	1990	3.59	(3.15 ,4.05)
Deaths	India	Both	1990	0.75	(0.47 ,1.05)
Deaths	Indonesia	Both	1990	0.26	(0.20 ,0.35)
Deaths	Iran	Both	1990	1.05	(0.83 ,1.32)
Deaths	Iraq	Both	1990	0.30	(0.21 ,0.43)
Deaths	Ireland	Both	1990	3.03	(2.74 ,3.42)
Deaths	Israel	Both	1990	3.83	(3.28 ,4.35)
Deaths	Italy	Both	1990	1.29	(1.05 ,1.65)
Deaths	Ivory Coast	Both	1990	0.56	(0.32 ,0.85)
Deaths	Jamaica	Both	1990	0.25	(0.21 ,0.30)
Deaths	Japan	Both	1990	2.81	(2.35 ,3.35)
Deaths	Jordan	Both	1990	0.85	(0.68 ,1.04)
Deaths	Kazakhstan	Both	1990	0.13	(0.09 ,0.16)
Deaths	Kenya	Both	1990	0.73	(0.58 ,0.97)
Deaths	Kiribati	Both	1990	0.74	(0.54 ,1.40)
Deaths	Kuwait	Both	1990	0.69	(0.55 ,0.81)
Deaths	Kyrgyzstan	Both	1990	0.18	(0.16 ,0.22)
Deaths	Laos	Both	1990	0.25	(0.15 ,0.42)
Deaths	Latvia	Both	1990	0.44	(0.38 ,0.49)
Deaths	Lebanon	Both	1990	1.29	(0.90 ,1.67)
Deaths	Lesotho	Both	1990	0.70	(0.47 ,1.04)
Deaths	Liberia	Both	1990	0.53	(0.29 ,0.82)
Deaths	Libya	Both	1990	0.74	(0.44 ,1.06)

Deaths	Lithuania	Both	1990	0.26	(0.21 ,0.30)
Deaths	Luxembourg	Both	1990	4.49	(3.92 ,5.16)
Deaths	Macedonia	Both	1990	0.46	(0.37 ,0.60)
Deaths	Madagascar	Both	1990	1.23	(0.86 ,1.70)
Deaths	Malawi	Both	1990	0.85	(0.62 ,1.21)
Deaths	Malaysia	Both	1990	0.39	(0.32 ,0.46)
Deaths	Maldives	Both	1990	0.51	(0.29 ,0.79)
Deaths	Mali	Both	1990	0.52	(0.24 ,0.91)
Deaths	Malta	Both	1990	1.86	(1.63 ,2.14)
Deaths	Marshall Islands	Both	1990	1.23	(0.76 ,1.77)
Deaths	Mauritania	Both	1990	0.63	(0.37 ,0.95)
Deaths	Mauritius	Both	1990	0.61	(0.50 ,0.78)
Deaths	Mexico	Both	1990	0.59	(0.54 ,0.69)
Deaths	Micronesia	Both	1990	1.25	(0.75 ,1.74)
Deaths	Monaco	Both	1990	1.08	(0.80 ,1.43)
Deaths	Mongolia	Both	1990	0.26	(0.16 ,0.45)
Deaths	Montenegro	Both	1990	0.34	(0.26 ,0.42)
Deaths	Morocco	Both	1990	0.90	(0.58 ,1.28)
Deaths	Mozambique	Both	1990	0.86	(0.56 ,1.23)
Deaths	Myanmar	Both	1990	0.29	(0.18 ,0.47)
Deaths	Namibia	Both	1990	0.82	(0.57 ,1.12)
Deaths	Nauru	Both	1990	1.39	(0.96 ,1.90)
Deaths	Nepal	Both	1990	0.65	(0.41 ,0.94)
Deaths	Netherlands	Both	1990	3.97	(3.26 ,5.50)
Deaths	New Zealand	Both	1990	4.43	(3.88 ,4.87)
Deaths	Nicaragua	Both	1990	0.38	(0.33 ,0.44)
Deaths	Niger	Both	1990	0.52	(0.25 ,0.83)
Deaths	Nigeria	Both	1990	0.66	(0.33 ,1.13)
Deaths	Niue	Both	1990	1.03	(0.77 ,1.37)
Deaths	North Korea	Both	1990	0.24	(0.17 ,0.34)
Deaths	Northern Mariana Islands	Both	1990	1.99	(1.54 ,2.53)
Deaths	Norway	Both	1990	5.55	(4.76 ,6.39)
Deaths	Oman	Both	1990	1.59	(1.13 ,2.10)
Deaths	Pakistan	Both	1990	0.76	(0.44 ,1.09)
Deaths	Palau	Both	1990	0.27	(0.20 ,0.38)
Deaths	Palestine	Both	1990	0.31	(0.22 ,0.46)
Deaths	Panama	Both	1990	0.88	(0.74 ,1.01)
Deaths	Papua New Guinea	Both	1990	0.73	(0.37 ,1.15)
Deaths	Paraguay	Both	1990	1.22	(0.98 ,1.44)
Deaths	Peru	Both	1990	0.52	(0.39 ,0.66)
Deaths	Philippines	Both	1990	0.14	(0.12 ,0.19)
Deaths	Poland	Both	1990	0.23	(0.16 ,0.33)
Deaths	Portugal	Both	1990	1.44	(1.07 ,1.66)
Deaths	Puerto Rico	Both	1990	1.43	(1.23 ,1.62)
Deaths	Qatar	Both	1990	2.06	(1.41 ,3.10)
Deaths	Republic of Congo	Both	1990	1.23	(0.78 ,1.74)
Deaths	Republic of Moldova	Both	1990	0.18	(0.15 ,0.21)
Deaths	Romania	Both	1990	0.58	(0.46 ,0.77)
Deaths	Russia	Both	1990	0.24	(0.19 ,0.27)
Deaths	Rwanda	Both	1990	1.27	(0.78 ,1.98)
Deaths	Saint Kitts	Both	1990	1.26	(0.97 ,1.53)

Deaths	Saint Lucia	Both	1990	1.10	(0.90 ,1.31)
Deaths	Saint Vincent and the Grenac	Both	1990	1.61	(1.27 ,1.84)
Deaths	Samoa	Both	1990	1.08	(0.81 ,1.39)
Deaths	San Marino	Both	1990	1.92	(1.43 ,2.47)
Deaths	Sao Tome and Principe	Both	1990	0.43	(0.27 ,0.62)
Deaths	Saudi Arabia	Both	1990	0.57	(0.32 ,0.81)
Deaths	Senegal	Both	1990	0.48	(0.30 ,0.69)
Deaths	Serbia	Both	1990	1.18	(0.79 ,1.51)
Deaths	Seychelles	Both	1990	0.65	(0.52 ,0.78)
Deaths	Sierra Leone	Both	1990	0.49	(0.28 ,0.75)
Deaths	Singapore	Both	1990	0.77	(0.65 ,0.88)
Deaths	Slovakia	Both	1990	0.36	(0.29 ,0.56)
Deaths	Slovenia	Both	1990	2.60	(1.95 ,3.59)
Deaths	Solomon Islands	Both	1990	0.99	(0.60 ,1.45)
Deaths	Somalia	Both	1990	1.00	(0.58 ,1.62)
Deaths	South Africa	Both	1990	0.93	(0.78 ,1.07)
Deaths	South Korea	Both	1990	0.52	(0.39 ,0.65)
Deaths	South Sudan	Both	1990	0.84	(0.53 ,1.23)
Deaths	Spain	Both	1990	3.21	(2.81 ,3.65)
Deaths	Sri Lanka	Both	1990	0.93	(0.78 ,1.10)
Deaths	Sudan	Both	1990	0.88	(0.51 ,1.30)
Deaths	Suriname	Both	1990	0.81	(0.67 ,0.95)
Deaths	Swaziland	Both	1990	0.77	(0.51 ,1.16)
Deaths	Sweden	Both	1990	4.01	(3.53 ,4.38)
Deaths	Switzerland	Both	1990	3.39	(2.76 ,3.98)
Deaths	Syrian	Both	1990	3.12	(2.02 ,4.21)
Deaths	Taiwan	Both	1990	0.64	(0.52 ,0.92)
Deaths	Tajikistan	Both	1990	0.16	(0.12 ,0.21)
Deaths	Tanzania	Both	1990	1.13	(0.83 ,1.59)
Deaths	Thailand	Both	1990	0.29	(0.23 ,0.36)
Deaths	Timor-Leste	Both	1990	0.18	(0.12 ,0.29)
Deaths	Togo	Both	1990	0.57	(0.34 ,0.87)
Deaths	Tokelau	Both	1990	0.95	(0.63 ,1.34)
Deaths	Tonga	Both	1990	0.86	(0.65 ,1.15)
Deaths	Trinidad	Both	1990	0.71	(0.57 ,0.84)
Deaths	Tunisia	Both	1990	0.96	(0.65 ,1.24)
Deaths	Turkey	Both	1990	1.45	(0.93 ,2.01)
Deaths	Turkmenistan	Both	1990	0.15	(0.11 ,0.18)
Deaths	Tuvalu	Both	1990	1.09	(0.69 ,1.54)
Deaths	Uganda	Both	1990	0.84	(0.61 ,1.22)
Deaths	UK	Both	1990	3.20	(2.78 ,3.42)
Deaths	Ukraine	Both	1990	0.15	(0.11 ,0.17)
Deaths	United Arab Emirates	Both	1990	1.21	(0.72 ,1.78)
Deaths	Uruguay	Both	1990	3.62	(3.15 ,4.03)
Deaths	USA	Both	1990	3.66	(3.22 ,3.99)
Deaths	Uzbekistan	Both	1990	0.10	(0.06 ,0.17)
Deaths	Vanuatu	Both	1990	0.99	(0.67 ,1.35)
Deaths	Venezuela	Both	1990	0.94	(0.76 ,1.08)
Deaths	Vietnam	Both	1990	0.32	(0.24 ,0.45)
Deaths	Virgin Islands	Both	1990	1.94	(1.57 ,2.40)
Deaths	Yemen	Both	1990	0.92	(0.51 ,1.41)

Deaths	Zambia	Both	1990	0.88	(0.58 ,1.24)
Deaths	Zimbabwe	Both	1990	0.78	(0.58 ,1.13)
DALYs	Afghanistan	Both	1990	21.74	(8.51 ,37.98)
DALYs	Albania	Both	1990	7.90	(6.20 ,11.11)
DALYs	Algeria	Both	1990	25.12	(18.78 ,33.23)
DALYs	American Samoa	Both	1990	21.27	(16.64 ,26.87)
DALYs	Andorra	Both	1990	26.98	(18.77 ,38.24)
DALYs	Angola	Both	1990	18.64	(10.78 ,29.67)
DALYs	Antigua	Both	1990	17.90	(14.81 ,21.10)
DALYs	Argentina	Both	1990	60.95	(53.89 ,68.84)
DALYs	Armenia	Both	1990	4.58	(3.86 ,5.30)
DALYs	Australia	Both	1990	50.08	(45.72 ,55.42)
DALYs	Austria	Both	1990	50.40	(43.48 ,54.62)
DALYs	Azerbaijan	Both	1990	3.68	(3.08 ,4.31)
DALYs	Bahamas	Both	1990	19.04	(15.90 ,22.00)
DALYs	Bahrain	Both	1990	23.56	(19.12 ,29.37)
DALYs	Bangladesh	Both	1990	13.47	(7.56 ,19.10)
DALYs	Barbados	Both	1990	21.99	(18.88 ,25.33)
DALYs	Belarus	Both	1990	3.24	(2.28 ,4.14)
DALYs	Belgium	Both	1990	57.09	(49.33 ,70.45)
DALYs	Belize	Both	1990	8.74	(5.28 ,12.76)
DALYs	Benin	Both	1990	15.28	(8.18 ,23.17)
DALYs	Bermuda	Both	1990	111.00	(83.07 ,129.79)
DALYs	Bhutan	Both	1990	11.78	(5.08 ,19.95)
DALYs	Bolivia	Both	1990	18.68	(10.82 ,28.87)
DALYs	Bosnia and Herzegovina	Both	1990	17.45	(11.94 ,22.72)
DALYs	Botswana	Both	1990	16.78	(11.77 ,24.28)
DALYs	Brazil	Both	1990	41.89	(36.98 ,45.93)
DALYs	Brunei Darussalam	Both	1990	32.39	(25.59 ,42.67)
DALYs	Bulgaria	Both	1990	6.13	(4.65 ,8.83)
DALYs	Burkina Faso	Both	1990	15.01	(7.44 ,23.16)
DALYs	Burundi	Both	1990	22.32	(11.79 ,39.25)
DALYs	Cabo Verde	Both	1990	12.31	(9.34 ,15.35)
DALYs	Cambodia	Both	1990	4.80	(2.89 ,8.01)
DALYs	Cameroon	Both	1990	15.08	(8.53 ,22.96)
DALYs	Canada	Both	1990	36.97	(33.27 ,42.23)
DALYs	Central African Republic	Both	1990	23.02	(12.05 ,38.18)
DALYs	Chad	Both	1990	12.67	(6.92 ,19.84)
DALYs	Chile	Both	1990	32.18	(28.23 ,35.38)
DALYs	China	Both	1990	4.58	(2.91 ,6.49)
DALYs	Colombia	Both	1990	35.31	(31.55 ,39.12)
DALYs	Comoros	Both	1990	18.38	(9.72 ,29.16)
DALYs	Cook Islands	Both	1990	11.14	(8.45 ,14.53)
DALYs	Costa Rica	Both	1990	22.34	(18.97 ,25.29)
DALYs	Croatia	Both	1990	11.31	(7.29 ,22.82)
DALYs	Cuba	Both	1990	22.91	(19.47 ,25.96)
DALYs	Cyprus	Both	1990	131.58	(102.22 ,165.53)
DALYs	Czechia	Both	1990	6.49	(4.73 ,11.21)
DALYs	Democratic Republic of the C	Both	1990	18.79	(11.41 ,30.95)
DALYs	Denmark	Both	1990	38.09	(33.51 ,42.90)
DALYs	Djibouti	Both	1990	19.11	(13.75 ,27.80)

DALYs	Dominica	Both	1990	19.67	(15.55 ,24.23)
DALYs	Dominican Republic	Both	1990	16.38	(13.10 ,19.44)
DALYs	Ecuador	Both	1990	13.47	(10.94 ,16.85)
DALYs	Egypt	Both	1990	25.27	(17.55 ,35.50)
DALYs	El Salvador	Both	1990	7.05	(5.80 ,8.31)
DALYs	Equatorial Guinea	Both	1990	19.85	(10.25 ,33.97)
DALYs	Eritrea	Both	1990	19.19	(8.51 ,33.01)
DALYs	Estonia	Both	1990	8.85	(6.34 ,10.25)
DALYs	Ethiopia	Both	1990	17.88	(7.59 ,29.90)
DALYs	Fiji	Both	1990	10.60	(8.63 ,13.20)
DALYs	Finland	Both	1990	61.46	(50.45 ,69.54)
DALYs	France	Both	1990	71.63	(64.97 ,79.97)
DALYs	Gabon	Both	1990	22.84	(14.66 ,34.98)
DALYs	Gambia	Both	1990	13.44	(7.62 ,20.34)
DALYs	Georgia	Both	1990	3.81	(2.99 ,4.65)
DALYs	Germany	Both	1990	70.93	(60.81 ,79.45)
DALYs	Ghana	Both	1990	11.97	(6.67 ,19.86)
DALYs	Greece	Both	1990	36.72	(31.56 ,39.87)
DALYs	Greenland	Both	1990	73.57	(37.90 ,120.52)
DALYs	Grenada	Both	1990	22.93	(15.40 ,30.37)
DALYs	Guam	Both	1990	32.45	(26.59 ,39.61)
DALYs	Guatemala	Both	1990	8.49	(5.88 ,11.23)
DALYs	Guinea	Both	1990	13.78	(7.71 ,21.27)
DALYs	Guinea-Bissau	Both	1990	18.89	(6.55 ,35.49)
DALYs	Guyana	Both	1990	69.74	(56.35 ,83.30)
DALYs	Haiti	Both	1990	29.17	(15.77 ,42.87)
DALYs	Honduras	Both	1990	12.28	(9.43 ,16.17)
DALYs	Hungary	Both	1990	50.61	(42.43 ,55.95)
DALYs	Iceland	Both	1990	52.15	(46.84 ,58.32)
DALYs	India	Both	1990	13.90	(8.39 ,20.00)
DALYs	Indonesia	Both	1990	5.49	(4.31 ,7.66)
DALYs	Iran	Both	1990	23.90	(18.79 ,29.57)
DALYs	Iraq	Both	1990	7.34	(5.21 ,10.48)
DALYs	Ireland	Both	1990	48.55	(44.46 ,53.66)
DALYs	Israel	Both	1990	49.92	(43.86 ,56.34)
DALYs	Italy	Both	1990	26.58	(23.99 ,32.41)
DALYs	Ivory Coast	Both	1990	14.73	(8.23 ,22.49)
DALYs	Jamaica	Both	1990	5.65	(4.32 ,6.58)
DALYs	Japan	Both	1990	42.36	(37.74 ,49.05)
DALYs	Jordan	Both	1990	18.08	(14.33 ,22.47)
DALYs	Kazakhstan	Both	1990	3.16	(2.49 ,3.82)
DALYs	Kenya	Both	1990	13.68	(10.73 ,19.26)
DALYs	Kiribati	Both	1990	17.47	(12.89 ,30.66)
DALYs	Kuwait	Both	1990	13.95	(11.66 ,16.05)
DALYs	Kyrgyzstan	Both	1990	5.09	(4.33 ,6.51)
DALYs	Laos	Both	1990	5.24	(2.96 ,9.66)
DALYs	Latvia	Both	1990	10.20	(8.86 ,11.50)
DALYs	Lebanon	Both	1990	29.92	(21.10 ,39.11)
DALYs	Lesotho	Both	1990	13.31	(8.60 ,19.87)
DALYs	Liberia	Both	1990	13.62	(7.34 ,20.96)
DALYs	Libya	Both	1990	17.86	(11.17 ,24.26)

DALYs	Lithuania	Both	1990	6.57	(5.17 ,7.73)
DALYs	Luxembourg	Both	1990	64.37	(57.77 ,73.43)
DALYs	Macedonia	Both	1990	8.79	(7.47 ,11.21)
DALYs	Madagascar	Both	1990	27.89	(18.16 ,42.46)
DALYs	Malawi	Both	1990	16.35	(11.26 ,23.42)
DALYs	Malaysia	Both	1990	8.30	(6.95 ,9.84)
DALYs	Maldives	Both	1990	11.38	(6.61 ,18.30)
DALYs	Mali	Both	1990	12.98	(5.71 ,23.05)
DALYs	Malta	Both	1990	26.85	(23.97 ,30.67)
DALYs	Marshall Islands	Both	1990	25.18	(14.93 ,37.97)
DALYs	Mauritania	Both	1990	16.15	(9.33 ,24.71)
DALYs	Mauritius	Both	1990	16.24	(12.97 ,20.68)
DALYs	Mexico	Both	1990	13.67	(12.72 ,15.49)
DALYs	Micronesia	Both	1990	25.95	(14.43 ,38.89)
DALYs	Monaco	Both	1990	16.01	(12.32 ,20.50)
DALYs	Mongolia	Both	1990	6.93	(4.26 ,11.63)
DALYs	Montenegro	Both	1990	7.93	(6.23 ,9.62)
DALYs	Morocco	Both	1990	19.73	(13.03 ,27.73)
DALYs	Mozambique	Both	1990	16.05	(9.98 ,23.30)
DALYs	Myanmar	Both	1990	6.52	(4.00 ,10.74)
DALYs	Namibia	Both	1990	15.03	(10.27 ,21.24)
DALYs	Nauru	Both	1990	29.94	(19.03 ,43.74)
DALYs	Nepal	Both	1990	12.09	(7.32 ,17.26)
DALYs	Netherlands	Both	1990	59.02	(50.15 ,79.59)
DALYs	New Zealand	Both	1990	64.39	(57.87 ,71.15)
DALYs	Nicaragua	Both	1990	9.46	(8.23 ,11.04)
DALYs	Niger	Both	1990	13.49	(6.39 ,22.53)
DALYs	Nigeria	Both	1990	16.67	(7.82 ,28.74)
DALYs	Niue	Both	1990	22.16	(16.44 ,30.04)
DALYs	North Korea	Both	1990	6.24	(4.38 ,9.22)
DALYs	Northern Mariana Islands	Both	1990	42.23	(32.65 ,54.50)
DALYs	Norway	Both	1990	74.26	(66.30 ,84.91)
DALYs	Oman	Both	1990	32.51	(23.07 ,43.26)
DALYs	Pakistan	Both	1990	14.18	(7.75 ,20.81)
DALYs	Palau	Both	1990	6.68	(4.65 ,9.23)
DALYs	Palestine	Both	1990	7.82	(5.05 ,11.73)
DALYs	Panama	Both	1990	20.75	(17.69 ,24.01)
DALYs	Papua New Guinea	Both	1990	14.30	(7.00 ,23.65)
DALYs	Paraguay	Both	1990	23.89	(19.22 ,28.63)
DALYs	Peru	Both	1990	12.43	(9.14 ,16.57)
DALYs	Philippines	Both	1990	2.55	(2.15 ,3.21)
DALYs	Poland	Both	1990	6.03	(4.50 ,8.47)
DALYs	Portugal	Both	1990	25.25	(20.93 ,29.16)
DALYs	Puerto Rico	Both	1990	26.25	(23.16 ,29.12)
DALYs	Qatar	Both	1990	30.30	(22.22 ,42.39)
DALYs	Republic of Congo	Both	1990	24.56	(14.56 ,38.10)
DALYs	Republic of Moldova	Both	1990	4.36	(3.60 ,5.00)
DALYs	Romania	Both	1990	14.44	(11.94 ,17.76)
DALYs	Russia	Both	1990	5.90	(4.60 ,6.74)
DALYs	Rwanda	Both	1990	25.97	(14.84 ,44.00)
DALYs	Saint Kitts	Both	1990	25.67	(18.74 ,31.97)

DALYs	Saint Lucia	Both	1990	22.35	(18.26 ,26.71)
DALYs	Saint Vincent and the Grenac	Both	1990	35.21	(27.92 ,40.90)
DALYs	Samoa	Both	1990	21.68	(16.06 ,28.79)
DALYs	San Marino	Both	1990	23.48	(18.52 ,28.97)
DALYs	Sao Tome and Principe	Both	1990	10.50	(6.61 ,15.41)
DALYs	Saudi Arabia	Both	1990	11.37	(6.78 ,15.80)
DALYs	Senegal	Both	1990	12.46	(7.77 ,17.95)
DALYs	Serbia	Both	1990	23.04	(16.44 ,28.96)
DALYs	Seychelles	Both	1990	13.29	(11.16 ,15.79)
DALYs	Sierra Leone	Both	1990	12.51	(7.08 ,19.51)
DALYs	Singapore	Both	1990	16.54	(14.63 ,19.28)
DALYs	Slovakia	Both	1990	8.79	(7.22 ,13.26)
DALYs	Slovenia	Both	1990	42.67	(31.88 ,58.47)
DALYs	Solomon Islands	Both	1990	21.40	(11.43 ,35.63)
DALYs	Somalia	Both	1990	21.24	(10.83 ,36.53)
DALYs	South Africa	Both	1990	18.96	(16.20 ,22.10)
DALYs	South Korea	Both	1990	10.13	(7.69 ,12.60)
DALYs	South Sudan	Both	1990	16.60	(10.05 ,25.65)
DALYs	Spain	Both	1990	56.95	(51.08 ,62.32)
DALYs	Sri Lanka	Both	1990	19.77	(16.69 ,23.27)
DALYs	Sudan	Both	1990	21.04	(11.64 ,32.31)
DALYs	Suriname	Both	1990	17.25	(14.28 ,20.59)
DALYs	Swaziland	Both	1990	14.59	(9.37 ,21.96)
DALYs	Sweden	Both	1990	57.71	(52.84 ,62.24)
DALYs	Switzerland	Both	1990	45.15	(37.66 ,52.50)
DALYs	Syrian	Both	1990	67.76	(45.17 ,96.09)
DALYs	Taiwan	Both	1990	9.72	(8.12 ,13.93)
DALYs	Tajikistan	Both	1990	4.11	(3.30 ,5.00)
DALYs	Tanzania	Both	1990	21.72	(15.92 ,31.34)
DALYs	Thailand	Both	1990	6.02	(4.77 ,7.54)
DALYs	Timor-Leste	Both	1990	3.67	(2.43 ,6.05)
DALYs	Togo	Both	1990	14.58	(8.75 ,22.57)
DALYs	Tokelau	Both	1990	18.75	(11.90 ,27.37)
DALYs	Tonga	Both	1990	16.69	(12.74 ,22.14)
DALYs	Trinidad	Both	1990	15.13	(12.40 ,18.25)
DALYs	Tunisia	Both	1990	20.82	(14.19 ,26.71)
DALYs	Turkey	Both	1990	29.68	(19.03 ,39.81)
DALYs	Turkmenistan	Both	1990	3.52	(2.84 ,4.12)
DALYs	Tuvalu	Both	1990	23.02	(13.92 ,33.86)
DALYs	Uganda	Both	1990	16.13	(11.42 ,23.49)
DALYs	UK	Both	1990	51.73	(46.51 ,54.78)
DALYs	Ukraine	Both	1990	3.53	(2.75 ,4.27)
DALYs	United Arab Emirates	Both	1990	28.63	(17.43 ,41.47)
DALYs	Uruguay	Both	1990	55.89	(49.87 ,61.76)
DALYs	USA	Both	1990	59.90	(55.17 ,65.08)
DALYs	Uzbekistan	Both	1990	2.45	(1.44 ,3.77)
DALYs	Vanuatu	Both	1990	19.01	(11.79 ,27.87)
DALYs	Venezuela	Both	1990	21.42	(17.53 ,24.09)
DALYs	Vietnam	Both	1990	6.59	(4.96 ,9.66)
DALYs	Virgin Islands	Both	1990	41.45	(33.72 ,50.68)
DALYs	Yemen	Both	1990	21.47	(11.27 ,35.02)

DALYs	Zambia	Both	1990	16.67	(10.31 ,24.77)
DALYs	Zimbabwe	Both	1990	14.68	(11.37 ,20.98)
Incidence	Afghanistan	Both	2019	0.53	(0.45 ,0.63)
Incidence	Albania	Both	2019	13.27	(10.95 ,15.96)
Incidence	Algeria	Both	2019	0.76	(0.65 ,0.91)
Incidence	American Samoa	Both	2019	13.70	(11.11 ,16.81)
Incidence	Andorra	Both	2019	12.63	(9.34 ,15.64)
Incidence	Angola	Both	2019	0.92	(0.83 ,1.03)
Incidence	Antigua	Both	2019	2.55	(2.25 ,2.87)
Incidence	Argentina	Both	2019	9.94	(8.83 ,11.32)
Incidence	Armenia	Both	2019	3.24	(2.68 ,3.95)
Incidence	Australia	Both	2019	46.56	(39.52 ,54.82)
Incidence	Austria	Both	2019	47.41	(41.16 ,53.39)
Incidence	Azerbaijan	Both	2019	2.55	(2.04 ,3.07)
Incidence	Bahamas	Both	2019	2.98	(2.60 ,3.37)
Incidence	Bahrain	Both	2019	3.90	(3.29 ,4.65)
Incidence	Bangladesh	Both	2019	0.84	(0.74 ,0.95)
Incidence	Barbados	Both	2019	4.08	(3.50 ,4.73)
Incidence	Belarus	Both	2019	13.25	(10.60 ,16.56)
Incidence	Belgium	Both	2019	3.39	(2.73 ,4.20)
Incidence	Belize	Both	2019	2.94	(2.41 ,3.89)
Incidence	Benin	Both	2019	0.63	(0.55 ,0.72)
Incidence	Bermuda	Both	2019	18.44	(15.63 ,21.85)
Incidence	Bhutan	Both	2019	1.32	(1.06 ,1.82)
Incidence	Bolivia	Both	2019	1.38	(1.21 ,1.58)
Incidence	Bosnia and Herzegovina	Both	2019	16.92	(13.97 ,20.38)
Incidence	Botswana	Both	2019	1.15	(1.01 ,1.31)
Incidence	Brazil	Both	2019	2.69	(2.29 ,3.15)
Incidence	Brunei Darussalam	Both	2019	18.50	(15.71 ,21.74)
Incidence	Bulgaria	Both	2019	19.09	(15.46 ,23.28)
Incidence	Burkina Faso	Both	2019	0.65	(0.58 ,0.74)
Incidence	Burundi	Both	2019	0.69	(0.61 ,0.78)
Incidence	Cabo Verde	Both	2019	0.69	(0.60 ,0.79)
Incidence	Cambodia	Both	2019	0.28	(0.24 ,0.34)
Incidence	Cameroon	Both	2019	0.85	(0.75 ,0.97)
Incidence	Canada	Both	2019	9.51	(8.25 ,10.90)
Incidence	Central African Republic	Both	2019	0.95	(0.85 ,1.05)
Incidence	Chad	Both	2019	0.52	(0.45 ,0.59)
Incidence	Chile	Both	2019	11.52	(9.69 ,13.59)
Incidence	China	Both	2019	2.61	(2.11 ,3.17)
Incidence	Colombia	Both	2019	2.30	(2.03 ,2.62)
Incidence	Comoros	Both	2019	0.85	(0.75 ,0.96)
Incidence	Cook Islands	Both	2019	8.30	(6.68 ,10.06)
Incidence	Costa Rica	Both	2019	3.48	(3.05 ,4.00)
Incidence	Croatia	Both	2019	43.43	(37.15 ,50.15)
Incidence	Cuba	Both	2019	3.39	(2.95 ,3.90)
Incidence	Cyprus	Both	2019	10.62	(8.57 ,13.03)
Incidence	Czechia	Both	2019	38.43	(32.04 ,46.39)
Incidence	Democratic Republic of the C	Both	2019	0.93	(0.82 ,1.05)
Incidence	Denmark	Both	2019	10.52	(8.64 ,12.87)
Incidence	Djibouti	Both	2019	0.88	(0.77 ,0.99)

Incidence	Dominica	Both	2019	3.27	(2.90 ,3.72)
Incidence	Dominican Republic	Both	2019	1.94	(1.70 ,2.22)
Incidence	Ecuador	Both	2019	7.93	(6.91 ,9.07)
Incidence	Egypt	Both	2019	2.00	(1.61 ,2.42)
Incidence	El Salvador	Both	2019	1.06	(0.83 ,1.39)
Incidence	Equatorial Guinea	Both	2019	1.49	(1.27 ,1.74)
Incidence	Eritrea	Both	2019	0.80	(0.71 ,0.89)
Incidence	Estonia	Both	2019	46.84	(34.97 ,59.58)
Incidence	Ethiopia	Both	2019	0.62	(0.52 ,0.74)
Incidence	Fiji	Both	2019	2.21	(1.83 ,2.68)
Incidence	Finland	Both	2019	30.59	(22.93 ,37.64)
Incidence	France	Both	2019	6.30	(5.01 ,7.85)
Incidence	Gabon	Both	2019	2.46	(2.04 ,2.91)
Incidence	Gambia	Both	2019	0.62	(0.54 ,0.71)
Incidence	Georgia	Both	2019	5.53	(4.67 ,6.48)
Incidence	Germany	Both	2019	11.26	(9.25 ,13.68)
Incidence	Ghana	Both	2019	0.64	(0.56 ,0.73)
Incidence	Greece	Both	2019	9.47	(7.67 ,11.66)
Incidence	Greenland	Both	2019	7.80	(6.81 ,8.79)
Incidence	Grenada	Both	2019	2.16	(1.80 ,2.58)
Incidence	Guam	Both	2019	13.20	(10.61 ,16.11)
Incidence	Guatemala	Both	2019	0.68	(0.58 ,0.80)
Incidence	Guinea	Both	2019	0.61	(0.54 ,0.70)
Incidence	Guinea-Bissau	Both	2019	0.67	(0.59 ,0.75)
Incidence	Guyana	Both	2019	3.48	(2.92 ,3.91)
Incidence	Haiti	Both	2019	1.48	(1.14 ,1.85)
Incidence	Honduras	Both	2019	0.89	(0.77 ,1.02)
Incidence	Hungary	Both	2019	56.24	(48.55 ,66.61)
Incidence	Iceland	Both	2019	11.36	(9.29 ,13.71)
Incidence	India	Both	2019	0.99	(0.83 ,1.17)
Incidence	Indonesia	Both	2019	0.46	(0.38 ,0.56)
Incidence	Iran	Both	2019	0.58	(0.48 ,0.70)
Incidence	Iraq	Both	2019	0.98	(0.81 ,1.20)
Incidence	Ireland	Both	2019	14.23	(10.67 ,17.10)
Incidence	Israel	Both	2019	3.32	(2.69 ,4.09)
Incidence	Italy	Both	2019	27.37	(23.23 ,32.13)
Incidence	Ivory Coast	Both	2019	0.72	(0.63 ,0.81)
Incidence	Jamaica	Both	2019	1.49	(1.19 ,2.00)
Incidence	Japan	Both	2019	27.40	(23.13 ,32.70)
Incidence	Jordan	Both	2019	1.97	(1.60 ,2.40)
Incidence	Kazakhstan	Both	2019	5.29	(4.25 ,6.40)
Incidence	Kenya	Both	2019	1.10	(0.92 ,1.31)
Incidence	Kiribati	Both	2019	1.04	(0.87 ,1.27)
Incidence	Kuwait	Both	2019	5.74	(4.70 ,6.98)
Incidence	Kyrgyzstan	Both	2019	0.77	(0.63 ,0.93)
Incidence	Laos	Both	2019	0.33	(0.28 ,0.39)
Incidence	Latvia	Both	2019	31.24	(25.50 ,38.07)
Incidence	Lebanon	Both	2019	1.22	(0.91 ,1.63)
Incidence	Lesotho	Both	2019	1.10	(0.98 ,1.23)
Incidence	Liberia	Both	2019	0.65	(0.55 ,0.76)
Incidence	Libya	Both	2019	1.53	(1.24 ,1.87)

Incidence	Lithuania	Both	2019	22.44	(18.52 ,26.94)
Incidence	Luxembourg	Both	2019	18.85	(15.13 ,23.14)
Incidence	Macedonia	Both	2019	16.36	(13.51 ,19.71)
Incidence	Madagascar	Both	2019	1.09	(0.98 ,1.20)
Incidence	Malawi	Both	2019	0.85	(0.74 ,0.96)
Incidence	Malaysia	Both	2019	1.14	(0.97 ,1.33)
Incidence	Maldives	Both	2019	0.87	(0.65 ,1.06)
Incidence	Mali	Both	2019	0.51	(0.44 ,0.58)
Incidence	Malta	Both	2019	7.64	(6.56 ,8.99)
Incidence	Marshall Islands	Both	2019	1.29	(1.07 ,1.57)
Incidence	Mauritania	Both	2019	0.74	(0.62 ,0.87)
Incidence	Mauritius	Both	2019	1.35	(1.13 ,1.61)
Incidence	Mexico	Both	2019	2.74	(2.29 ,3.20)
Incidence	Micronesia	Both	2019	2.55	(2.11 ,3.11)
Incidence	Monaco	Both	2019	18.35	(15.25 ,22.61)
Incidence	Mongolia	Both	2019	0.81	(0.67 ,0.97)
Incidence	Montenegro	Both	2019	22.74	(18.30 ,27.51)
Incidence	Morocco	Both	2019	0.67	(0.56 ,0.81)
Incidence	Mozambique	Both	2019	0.98	(0.88 ,1.09)
Incidence	Myanmar	Both	2019	0.29	(0.24 ,0.35)
Incidence	Namibia	Both	2019	0.98	(0.87 ,1.10)
Incidence	Nauru	Both	2019	1.81	(1.52 ,2.14)
Incidence	Nepal	Both	2019	0.89	(0.79 ,1.01)
Incidence	Netherlands	Both	2019	2.96	(2.43 ,3.60)
Incidence	New Zealand	Both	2019	32.99	(27.83 ,39.00)
Incidence	Nicaragua	Both	2019	0.76	(0.62 ,0.94)
Incidence	Niger	Both	2019	0.48	(0.41 ,0.55)
Incidence	Nigeria	Both	2019	0.51	(0.43 ,0.62)
Incidence	Niue	Both	2019	3.93	(3.24 ,4.74)
Incidence	North Korea	Both	2019	0.36	(0.29 ,0.44)
Incidence	Northern Mariana Islands	Both	2019	9.58	(7.77 ,11.52)
Incidence	Norway	Both	2019	5.21	(4.16 ,6.58)
Incidence	Oman	Both	2019	1.43	(1.18 ,1.81)
Incidence	Pakistan	Both	2019	1.43	(1.20 ,1.68)
Incidence	Palau	Both	2019	4.30	(3.47 ,5.32)
Incidence	Palestine	Both	2019	0.60	(0.45 ,0.79)
Incidence	Panama	Both	2019	1.15	(0.99 ,1.32)
Incidence	Papua New Guinea	Both	2019	0.96	(0.80 ,1.13)
Incidence	Paraguay	Both	2019	3.34	(3.06 ,3.64)
Incidence	Peru	Both	2019	2.22	(1.83 ,2.67)
Incidence	Philippines	Both	2019	0.33	(0.27 ,0.40)
Incidence	Poland	Both	2019	21.33	(17.56 ,25.32)
Incidence	Portugal	Both	2019	4.06	(3.30 ,4.99)
Incidence	Puerto Rico	Both	2019	23.76	(19.11 ,29.19)
Incidence	Qatar	Both	2019	8.73	(7.08 ,10.63)
Incidence	Republic of Congo	Both	2019	1.11	(1.00 ,1.24)
Incidence	Republic of Moldova	Both	2019	15.45	(12.29 ,18.94)
Incidence	Romania	Both	2019	54.92	(44.76 ,68.05)
Incidence	Russia	Both	2019	25.93	(20.93 ,31.18)
Incidence	Rwanda	Both	2019	0.88	(0.77 ,1.00)
Incidence	Saint Kitts	Both	2019	3.05	(2.65 ,3.50)

Incidence	Saint Lucia	Both	2019	2.80	(2.49 ,3.18)
Incidence	Saint Vincent and the Grenac	Both	2019	2.71	(2.40 ,3.08)
Incidence	Samoa	Both	2019	2.61	(2.17 ,3.14)
Incidence	San Marino	Both	2019	12.00	(9.34 ,14.36)
Incidence	Sao Tome and Principe	Both	2019	0.67	(0.58 ,0.77)
Incidence	Saudi Arabia	Both	2019	2.91	(2.40 ,3.47)
Incidence	Senegal	Both	2019	0.50	(0.43 ,0.58)
Incidence	Serbia	Both	2019	28.27	(24.09 ,33.46)
Incidence	Seychelles	Both	2019	1.80	(1.39 ,2.34)
Incidence	Sierra Leone	Both	2019	0.56	(0.48 ,0.64)
Incidence	Singapore	Both	2019	24.41	(19.96 ,29.29)
Incidence	Slovakia	Both	2019	22.65	(19.02 ,26.87)
Incidence	Slovenia	Both	2019	62.21	(45.88 ,79.83)
Incidence	Solomon Islands	Both	2019	1.12	(0.94 ,1.34)
Incidence	Somalia	Both	2019	0.70	(0.62 ,0.79)
Incidence	South Africa	Both	2019	8.83	(7.04 ,10.76)
Incidence	South Korea	Both	2019	17.58	(14.74 ,20.76)
Incidence	South Sudan	Both	2019	0.81	(0.71 ,0.92)
Incidence	Spain	Both	2019	6.76	(5.44 ,8.26)
Incidence	Sri Lanka	Both	2019	0.59	(0.51 ,0.69)
Incidence	Sudan	Both	2019	0.63	(0.54 ,0.74)
Incidence	Suriname	Both	2019	2.40	(2.10 ,2.74)
Incidence	Swaziland	Both	2019	1.77	(1.31 ,2.26)
Incidence	Sweden	Both	2019	20.75	(17.04 ,25.30)
Incidence	Switzerland	Both	2019	18.00	(15.31 ,20.94)
Incidence	Syrian	Both	2019	1.04	(0.93 ,1.16)
Incidence	Taiwan	Both	2019	3.94	(3.30 ,4.71)
Incidence	Tajikistan	Both	2019	0.44	(0.36 ,0.52)
Incidence	Tanzania	Both	2019	0.91	(0.80 ,1.04)
Incidence	Thailand	Both	2019	1.00	(0.85 ,1.17)
Incidence	Timor-Leste	Both	2019	0.31	(0.26 ,0.36)
Incidence	Togo	Both	2019	0.65	(0.58 ,0.73)
Incidence	Tokelau	Both	2019	1.83	(1.53 ,2.19)
Incidence	Tonga	Both	2019	3.21	(2.64 ,3.88)
Incidence	Trinidad	Both	2019	3.17	(2.61 ,3.89)
Incidence	Tunisia	Both	2019	0.83	(0.70 ,1.02)
Incidence	Turkey	Both	2019	1.76	(1.60 ,1.95)
Incidence	Turkmenistan	Both	2019	2.52	(2.04 ,3.03)
Incidence	Tuvalu	Both	2019	1.17	(0.97 ,1.38)
Incidence	Uganda	Both	2019	0.77	(0.68 ,0.88)
Incidence	UK	Both	2019	25.86	(21.74 ,30.53)
Incidence	Ukraine	Both	2019	13.38	(10.64 ,16.36)
Incidence	United Arab Emirates	Both	2019	6.05	(4.97 ,7.40)
Incidence	Uruguay	Both	2019	12.95	(11.39 ,14.81)
Incidence	USA	Both	2019	21.52	(18.69 ,24.60)
Incidence	Uzbekistan	Both	2019	2.17	(1.81 ,2.55)
Incidence	Vanuatu	Both	2019	1.36	(1.13 ,1.63)
Incidence	Venezuela	Both	2019	1.75	(1.54 ,1.99)
Incidence	Vietnam	Both	2019	0.33	(0.28 ,0.40)
Incidence	Virgin Islands	Both	2019	15.62	(12.96 ,18.56)
Incidence	Yemen	Both	2019	0.60	(0.53 ,0.70)

Incidence	Zambia	Both	2019	0.88	(0.78 ,0.99)
Incidence	Zimbabwe	Both	2019	0.96	(0.86 ,1.07)
Prevalence	Afghanistan	Both	2019	1.04	(0.80 ,1.34)
Prevalence	Albania	Both	2019	239.18	(199.20 ,289.35)
Prevalence	Algeria	Both	2019	4.64	(3.63 ,5.84)
Prevalence	American Samoa	Both	2019	195.91	(153.90 ,245.53)
Prevalence	Andorra	Both	2019	171.77	(130.03 ,213.02)
Prevalence	Angola	Both	2019	1.20	(0.95 ,1.50)
Prevalence	Antigua	Both	2019	29.65	(24.38 ,35.08)
Prevalence	Argentina	Both	2019	102.43	(86.97 ,122.81)
Prevalence	Armenia	Both	2019	55.18	(44.90 ,67.49)
Prevalence	Australia	Both	2019	695.19	(590.19 ,832.23)
Prevalence	Austria	Both	2019	668.14	(576.67 ,759.72)
Prevalence	Azerbaijan	Both	2019	45.27	(36.12 ,55.52)
Prevalence	Bahamas	Both	2019	38.24	(31.29 ,45.62)
Prevalence	Bahrain	Both	2019	40.24	(31.18 ,50.80)
Prevalence	Bangladesh	Both	2019	1.64	(1.28 ,2.04)
Prevalence	Barbados	Both	2019	57.18	(46.41 ,68.28)
Prevalence	Belarus	Both	2019	229.73	(183.94 ,280.39)
Prevalence	Belgium	Both	2019	39.00	(30.82 ,48.99)
Prevalence	Belize	Both	2019	42.70	(33.90 ,55.26)
Prevalence	Benin	Both	2019	1.65	(1.27 ,2.08)
Prevalence	Bermuda	Both	2019	274.80	(231.59 ,328.97)
Prevalence	Bhutan	Both	2019	4.95	(3.67 ,6.65)
Prevalence	Bolivia	Both	2019	15.70	(12.98 ,19.01)
Prevalence	Bosnia and Herzegovina	Both	2019	294.68	(245.88 ,354.85)
Prevalence	Botswana	Both	2019	5.98	(4.64 ,7.49)
Prevalence	Brazil	Both	2019	23.70	(19.11 ,29.00)
Prevalence	Brunei Darussalam	Both	2019	289.41	(246.36 ,342.68)
Prevalence	Bulgaria	Both	2019	357.88	(289.22 ,429.53)
Prevalence	Burkina Faso	Both	2019	1.39	(1.08 ,1.74)
Prevalence	Burundi	Both	2019	0.78	(0.61 ,0.98)
Prevalence	Cabo Verde	Both	2019	3.42	(2.71 ,4.24)
Prevalence	Cambodia	Both	2019	1.15	(0.85 ,1.53)
Prevalence	Cameroon	Both	2019	3.05	(2.37 ,3.86)
Prevalence	Canada	Both	2019	100.80	(83.69 ,119.91)
Prevalence	Central African Republic	Both	2019	0.82	(0.65 ,1.01)
Prevalence	Chad	Both	2019	0.99	(0.78 ,1.26)
Prevalence	Chile	Both	2019	166.28	(138.23 ,198.59)
Prevalence	China	Both	2019	42.75	(34.03 ,52.12)
Prevalence	Colombia	Both	2019	27.56	(22.58 ,33.42)
Prevalence	Comoros	Both	2019	1.47	(1.14 ,1.85)
Prevalence	Cook Islands	Both	2019	118.44	(91.50 ,146.01)
Prevalence	Costa Rica	Both	2019	48.79	(40.59 ,58.17)
Prevalence	Croatia	Both	2019	761.43	(654.39 ,881.08)
Prevalence	Cuba	Both	2019	49.03	(40.91 ,58.48)
Prevalence	Cyprus	Both	2019	131.73	(106.46 ,162.28)
Prevalence	Czechia	Both	2019	676.09	(559.92 ,802.00)
Prevalence	Democratic Republic of the C	Both	2019	1.12	(0.88 ,1.41)
Prevalence	Denmark	Both	2019	142.90	(116.33 ,175.93)
Prevalence	Djibouti	Both	2019	1.55	(1.22 ,1.94)

Prevalence	Dominica	Both	2019	37.31	(30.66 ,44.91)
Prevalence	Dominican Republic	Both	2019	23.21	(19.20 ,27.72)
Prevalence	Ecuador	Both	2019	132.90	(114.58 ,153.07)
Prevalence	Egypt	Both	2019	13.43	(10.24 ,17.40)
Prevalence	El Salvador	Both	2019	14.48	(11.13 ,18.99)
Prevalence	Equatorial Guinea	Both	2019	4.01	(3.01 ,5.09)
Prevalence	Eritrea	Both	2019	0.86	(0.68 ,1.07)
Prevalence	Estonia	Both	2019	759.94	(578.58 ,943.34)
Prevalence	Ethiopia	Both	2019	1.05	(0.81 ,1.35)
Prevalence	Fiji	Both	2019	22.15	(17.46 ,27.72)
Prevalence	Finland	Both	2019	432.08	(330.68 ,536.75)
Prevalence	France	Both	2019	79.49	(63.32 ,99.16)
Prevalence	Gabon	Both	2019	10.01	(7.57 ,12.90)
Prevalence	Gambia	Both	2019	1.57	(1.23 ,1.96)
Prevalence	Georgia	Both	2019	92.12	(76.88 ,110.29)
Prevalence	Germany	Both	2019	147.90	(121.44 ,179.61)
Prevalence	Ghana	Both	2019	1.89	(1.47 ,2.33)
Prevalence	Greece	Both	2019	126.19	(103.74 ,154.94)
Prevalence	Greenland	Both	2019	45.34	(37.49 ,54.11)
Prevalence	Grenada	Both	2019	22.06	(17.49 ,27.16)
Prevalence	Guam	Both	2019	189.76	(149.17 ,234.59)
Prevalence	Guatemala	Both	2019	6.42	(5.02 ,7.93)
Prevalence	Guinea	Both	2019	1.25	(0.99 ,1.57)
Prevalence	Guinea-Bissau	Both	2019	1.16	(0.92 ,1.45)
Prevalence	Guyana	Both	2019	29.20	(23.48 ,35.73)
Prevalence	Haiti	Both	2019	7.17	(5.36 ,9.18)
Prevalence	Honduras	Both	2019	8.74	(7.08 ,10.67)
Prevalence	Hungary	Both	2019	997.07	(861.74 ,1167.68)
Prevalence	Iceland	Both	2019	155.92	(128.08 ,189.36)
Prevalence	India	Both	2019	1.94	(1.52 ,2.42)
Prevalence	Indonesia	Both	2019	2.72	(2.09 ,3.50)
Prevalence	Iran	Both	2019	4.18	(3.26 ,5.23)
Prevalence	Iraq	Both	2019	5.67	(4.30 ,7.39)
Prevalence	Ireland	Both	2019	192.79	(145.96 ,234.79)
Prevalence	Israel	Both	2019	37.58	(29.51 ,47.60)
Prevalence	Italy	Both	2019	408.26	(347.04 ,476.32)
Prevalence	Ivory Coast	Both	2019	1.72	(1.35 ,2.16)
Prevalence	Jamaica	Both	2019	20.35	(15.71 ,27.14)
Prevalence	Japan	Both	2019	435.47	(369.13 ,514.16)
Prevalence	Jordan	Both	2019	18.74	(14.28 ,23.93)
Prevalence	Kazakhstan	Both	2019	97.94	(77.96 ,117.06)
Prevalence	Kenya	Both	2019	2.77	(2.12 ,3.52)
Prevalence	Kiribati	Both	2019	6.50	(4.98 ,8.33)
Prevalence	Kuwait	Both	2019	85.34	(66.96 ,106.72)
Prevalence	Kyrgyzstan	Both	2019	12.13	(9.57 ,14.79)
Prevalence	Laos	Both	2019	1.11	(0.82 ,1.49)
Prevalence	Latvia	Both	2019	526.67	(435.44 ,631.71)
Prevalence	Lebanon	Both	2019	11.26	(8.34 ,15.04)
Prevalence	Lesotho	Both	2019	3.28	(2.60 ,4.08)
Prevalence	Liberia	Both	2019	2.18	(1.65 ,2.81)
Prevalence	Libya	Both	2019	11.24	(8.49 ,14.42)

Prevalence	Lithuania	Both	2019	380.23	(316.79 ,452.87)
Prevalence	Luxembourg	Both	2019	264.10	(215.26 ,323.76)
Prevalence	Macedonia	Both	2019	307.54	(253.65 ,370.57)
Prevalence	Madagascar	Both	2019	1.43	(1.15 ,1.75)
Prevalence	Malawi	Both	2019	1.19	(0.91 ,1.53)
Prevalence	Malaysia	Both	2019	10.91	(8.63 ,13.67)
Prevalence	Maldives	Both	2019	6.77	(5.23 ,8.68)
Prevalence	Mali	Both	2019	1.11	(0.86 ,1.39)
Prevalence	Malta	Both	2019	99.79	(83.46 ,119.84)
Prevalence	Marshall Islands	Both	2019	8.03	(6.34 ,10.14)
Prevalence	Mauritania	Both	2019	3.33	(2.55 ,4.20)
Prevalence	Mauritius	Both	2019	14.25	(11.15 ,18.11)
Prevalence	Mexico	Both	2019	41.39	(33.68 ,49.40)
Prevalence	Micronesia	Both	2019	22.73	(18.04 ,28.83)
Prevalence	Monaco	Both	2019	291.41	(242.55 ,359.55)
Prevalence	Mongolia	Both	2019	11.74	(9.38 ,14.38)
Prevalence	Montenegro	Both	2019	433.64	(352.66 ,524.66)
Prevalence	Morocco	Both	2019	3.24	(2.50 ,4.04)
Prevalence	Mozambique	Both	2019	1.22	(0.96 ,1.51)
Prevalence	Myanmar	Both	2019	1.20	(0.87 ,1.60)
Prevalence	Namibia	Both	2019	2.90	(2.34 ,3.54)
Prevalence	Nauru	Both	2019	17.20	(13.58 ,21.33)
Prevalence	Nepal	Both	2019	2.12	(1.65 ,2.67)
Prevalence	Netherlands	Both	2019	30.87	(24.52 ,39.18)
Prevalence	New Zealand	Both	2019	405.99	(342.78 ,477.05)
Prevalence	Nicaragua	Both	2019	10.36	(8.19 ,12.70)
Prevalence	Niger	Both	2019	0.98	(0.75 ,1.25)
Prevalence	Nigeria	Both	2019	1.60	(1.24 ,2.01)
Prevalence	Niue	Both	2019	49.02	(38.72 ,60.88)
Prevalence	North Korea	Both	2019	3.66	(2.84 ,4.66)
Prevalence	Northern Mariana Islands	Both	2019	128.64	(102.16 ,159.79)
Prevalence	Norway	Both	2019	57.49	(45.28 ,73.06)
Prevalence	Oman	Both	2019	11.96	(9.11 ,15.24)
Prevalence	Pakistan	Both	2019	3.41	(2.69 ,4.25)
Prevalence	Palau	Both	2019	56.57	(44.48 ,71.27)
Prevalence	Palestine	Both	2019	3.29	(2.43 ,4.30)
Prevalence	Panama	Both	2019	15.29	(12.49 ,18.55)
Prevalence	Papua New Guinea	Both	2019	5.13	(3.99 ,6.35)
Prevalence	Paraguay	Both	2019	25.43	(21.13 ,30.44)
Prevalence	Peru	Both	2019	35.90	(29.07 ,43.60)
Prevalence	Philippines	Both	2019	1.73	(1.28 ,2.31)
Prevalence	Poland	Both	2019	382.71	(310.52 ,454.22)
Prevalence	Portugal	Both	2019	46.47	(37.66 ,57.77)
Prevalence	Puerto Rico	Both	2019	457.61	(362.96 ,575.53)
Prevalence	Qatar	Both	2019	137.22	(105.59 ,171.60)
Prevalence	Republic of Congo	Both	2019	1.77	(1.37 ,2.22)
Prevalence	Republic of Moldova	Both	2019	274.15	(219.12 ,333.46)
Prevalence	Romania	Both	2019	1044.49	(855.59 ,1271.28)
Prevalence	Russia	Both	2019	456.35	(365.58 ,553.15)
Prevalence	Rwanda	Both	2019	1.22	(0.94 ,1.53)
Prevalence	Saint Kitts	Both	2019	39.52	(32.26 ,47.20)

Prevalence	Saint Lucia	Both	2019	32.61	(26.88 ,39.31)
Prevalence	Saint Vincent and the Grenac	Both	2019	30.25	(24.94 ,36.43)
Prevalence	Samoa	Both	2019	26.44	(21.08 ,33.10)
Prevalence	San Marino	Both	2019	169.43	(134.48 ,203.70)
Prevalence	Sao Tome and Principe	Both	2019	2.76	(2.11 ,3.40)
Prevalence	Saudi Arabia	Both	2019	30.66	(23.69 ,38.07)
Prevalence	Senegal	Both	2019	1.34	(1.05 ,1.70)
Prevalence	Serbia	Both	2019	492.89	(417.41 ,579.51)
Prevalence	Seychelles	Both	2019	20.89	(15.47 ,27.26)
Prevalence	Sierra Leone	Both	2019	1.27	(1.00 ,1.60)
Prevalence	Singapore	Both	2019	433.97	(358.54 ,518.38)
Prevalence	Slovakia	Both	2019	410.75	(342.55 ,482.30)
Prevalence	Slovenia	Both	2019	1080.06	(825.88 ,1367.13)
Prevalence	Solomon Islands	Both	2019	6.73	(5.28 ,8.49)
Prevalence	Somalia	Both	2019	0.66	(0.52 ,0.85)
Prevalence	South Africa	Both	2019	118.85	(91.51 ,151.35)
Prevalence	South Korea	Both	2019	310.27	(261.14 ,364.94)
Prevalence	South Sudan	Both	2019	1.21	(0.92 ,1.56)
Prevalence	Spain	Both	2019	85.32	(69.27 ,105.43)
Prevalence	Sri Lanka	Both	2019	4.07	(3.16 ,5.14)
Prevalence	Sudan	Both	2019	2.33	(1.82 ,2.97)
Prevalence	Suriname	Both	2019	27.52	(22.30 ,33.20)
Prevalence	Swaziland	Both	2019	10.39	(7.14 ,13.98)
Prevalence	Sweden	Both	2019	284.59	(234.80 ,343.78)
Prevalence	Switzerland	Both	2019	248.78	(210.62 ,294.25)
Prevalence	Syrian	Both	2019	5.57	(4.40 ,6.86)
Prevalence	Taiwan	Both	2019	55.11	(45.84 ,66.97)
Prevalence	Tajikistan	Both	2019	5.72	(4.47 ,7.19)
Prevalence	Tanzania	Both	2019	1.54	(1.18 ,1.95)
Prevalence	Thailand	Both	2019	9.52	(7.44 ,11.92)
Prevalence	Timor-Leste	Both	2019	1.18	(0.88 ,1.55)
Prevalence	Togo	Both	2019	1.48	(1.17 ,1.82)
Prevalence	Tokelau	Both	2019	18.42	(14.59 ,23.16)
Prevalence	Tonga	Both	2019	35.43	(28.24 ,44.19)
Prevalence	Trinidad	Both	2019	43.89	(35.16 ,53.79)
Prevalence	Tunisia	Both	2019	6.38	(5.00 ,8.03)
Prevalence	Turkey	Both	2019	12.30	(9.82 ,14.89)
Prevalence	Turkmenistan	Both	2019	44.16	(35.28 ,53.39)
Prevalence	Tuvalu	Both	2019	9.09	(7.06 ,11.40)
Prevalence	Uganda	Both	2019	1.06	(0.82 ,1.33)
Prevalence	UK	Both	2019	341.24	(287.07 ,405.19)
Prevalence	Ukraine	Both	2019	232.44	(185.06 ,282.36)
Prevalence	United Arab Emirates	Both	2019	69.23	(53.50 ,88.41)
Prevalence	Uruguay	Both	2019	129.03	(108.73 ,153.35)
Prevalence	USA	Both	2019	261.92	(229.32 ,299.88)
Prevalence	Uzbekistan	Both	2019	33.60	(27.16 ,40.40)
Prevalence	Vanuatu	Both	2019	8.39	(6.57 ,10.66)
Prevalence	Venezuela	Both	2019	21.17	(17.36 ,25.42)
Prevalence	Vietnam	Both	2019	1.93	(1.44 ,2.52)
Prevalence	Virgin Islands	Both	2019	263.88	(211.63 ,324.00)
Prevalence	Yemen	Both	2019	1.67	(1.31 ,2.11)

Prevalence	Zambia	Both	2019	1.38	(1.06 ,1.76)
Prevalence	Zimbabwe	Both	2019	2.27	(1.83 ,2.77)
Deaths	Afghanistan	Both	2019	0.87	(0.44 ,1.31)
Deaths	Albania	Both	2019	0.56	(0.39 ,0.75)
Deaths	Algeria	Both	2019	0.98	(0.65 ,1.30)
Deaths	American Samoa	Both	2019	1.05	(0.83 ,1.32)
Deaths	Andorra	Both	2019	1.92	(1.39 ,2.53)
Deaths	Angola	Both	2019	1.06	(0.80 ,1.38)
Deaths	Antigua	Both	2019	0.94	(0.77 ,1.19)
Deaths	Argentina	Both	2019	3.87	(3.34 ,4.30)
Deaths	Armenia	Both	2019	0.38	(0.32 ,0.46)
Deaths	Australia	Both	2019	2.89	(2.36 ,3.31)
Deaths	Austria	Both	2019	4.31	(3.52 ,4.96)
Deaths	Azerbaijan	Both	2019	0.19	(0.15 ,0.24)
Deaths	Bahamas	Both	2019	0.92	(0.75 ,1.14)
Deaths	Bahrain	Both	2019	1.42	(1.12 ,1.79)
Deaths	Bangladesh	Both	2019	0.85	(0.56 ,1.22)
Deaths	Barbados	Both	2019	1.11	(0.92 ,1.33)
Deaths	Belarus	Both	2019	0.18	(0.14 ,0.24)
Deaths	Belgium	Both	2019	5.44	(4.33 ,6.21)
Deaths	Belize	Both	2019	0.51	(0.41 ,0.64)
Deaths	Benin	Both	2019	0.60	(0.36 ,0.92)
Deaths	Bermuda	Both	2019	3.51	(2.90 ,4.29)
Deaths	Bhutan	Both	2019	0.91	(0.61 ,1.25)
Deaths	Bolivia	Both	2019	0.88	(0.62 ,1.22)
Deaths	Bosnia and Herzegovina	Both	2019	1.45	(1.11 ,1.89)
Deaths	Botswana	Both	2019	0.89	(0.62 ,1.32)
Deaths	Brazil	Both	2019	1.54	(1.33 ,1.78)
Deaths	Brunei Darussalam	Both	2019	3.29	(2.59 ,3.99)
Deaths	Bulgaria	Both	2019	0.48	(0.36 ,0.62)
Deaths	Burkina Faso	Both	2019	0.73	(0.44 ,1.14)
Deaths	Burundi	Both	2019	0.81	(0.57 ,1.17)
Deaths	Cabo Verde	Both	2019	0.70	(0.55 ,0.90)
Deaths	Cambodia	Both	2019	0.22	(0.16 ,0.34)
Deaths	Cameroon	Both	2019	0.68	(0.43 ,1.04)
Deaths	Canada	Both	2019	2.93	(2.40 ,3.28)
Deaths	Central African Republic	Both	2019	1.04	(0.61 ,1.53)
Deaths	Chad	Both	2019	0.54	(0.30 ,0.86)
Deaths	Chile	Both	2019	1.41	(1.24 ,1.62)
Deaths	China	Both	2019	0.15	(0.12 ,0.18)
Deaths	Colombia	Both	2019	1.25	(0.90 ,1.76)
Deaths	Comoros	Both	2019	0.85	(0.62 ,1.16)
Deaths	Cook Islands	Both	2019	0.32	(0.26 ,0.41)
Deaths	Costa Rica	Both	2019	1.31	(1.00 ,1.69)
Deaths	Croatia	Both	2019	3.04	(2.13 ,3.89)
Deaths	Cuba	Both	2019	1.03	(0.80 ,1.37)
Deaths	Cyprus	Both	2019	8.20	(6.86 ,9.76)
Deaths	Czechia	Both	2019	2.69	(1.97 ,3.51)
Deaths	Democratic Republic of the C	Both	2019	0.97	(0.66 ,1.38)
Deaths	Denmark	Both	2019	4.53	(3.69 ,5.15)
Deaths	Djibouti	Both	2019	0.99	(0.66 ,1.43)

Deaths	Dominica	Both	2019	1.03	(0.79 ,1.32)
Deaths	Dominican Republic	Both	2019	0.85	(0.66 ,1.09)
Deaths	Ecuador	Both	2019	0.86	(0.66 ,1.11)
Deaths	Egypt	Both	2019	1.11	(0.79 ,1.54)
Deaths	El Salvador	Both	2019	0.27	(0.20 ,0.35)
Deaths	Equatorial Guinea	Both	2019	1.15	(0.74 ,1.64)
Deaths	Eritrea	Both	2019	0.95	(0.66 ,1.32)
Deaths	Estonia	Both	2019	2.57	(1.89 ,3.45)
Deaths	Ethiopia	Both	2019	0.71	(0.53 ,1.01)
Deaths	Fiji	Both	2019	0.43	(0.33 ,0.54)
Deaths	Finland	Both	2019	4.90	(3.95 ,5.67)
Deaths	France	Both	2019	4.04	(3.28 ,4.61)
Deaths	Gabon	Both	2019	1.23	(0.94 ,1.60)
Deaths	Gambia	Both	2019	0.62	(0.40 ,0.93)
Deaths	Georgia	Both	2019	0.69	(0.49 ,1.05)
Deaths	Germany	Both	2019	5.47	(4.61 ,6.26)
Deaths	Ghana	Both	2019	0.59	(0.38 ,0.88)
Deaths	Greece	Both	2019	3.33	(2.83 ,3.83)
Deaths	Greenland	Both	2019	4.37	(2.66 ,6.15)
Deaths	Grenada	Both	2019	1.12	(0.93 ,1.44)
Deaths	Guam	Both	2019	1.57	(1.26 ,1.90)
Deaths	Guatemala	Both	2019	0.35	(0.28 ,0.44)
Deaths	Guinea	Both	2019	0.61	(0.36 ,0.96)
Deaths	Guinea-Bissau	Both	2019	0.71	(0.37 ,1.19)
Deaths	Guyana	Both	2019	1.90	(1.49 ,2.44)
Deaths	Haiti	Both	2019	1.22	(0.73 ,1.82)
Deaths	Honduras	Both	2019	0.81	(0.64 ,1.02)
Deaths	Hungary	Both	2019	3.95	(3.11 ,4.80)
Deaths	Iceland	Both	2019	3.70	(2.96 ,4.24)
Deaths	India	Both	2019	0.73	(0.56 ,0.94)
Deaths	Indonesia	Both	2019	0.30	(0.21 ,0.42)
Deaths	Iran	Both	2019	0.90	(0.80 ,1.11)
Deaths	Iraq	Both	2019	0.35	(0.25 ,0.46)
Deaths	Ireland	Both	2019	3.04	(2.55 ,3.47)
Deaths	Israel	Both	2019	3.68	(3.02 ,4.18)
Deaths	Italy	Both	2019	2.30	(1.83 ,2.59)
Deaths	Ivory Coast	Both	2019	0.56	(0.34 ,0.84)
Deaths	Jamaica	Both	2019	0.31	(0.24 ,0.41)
Deaths	Japan	Both	2019	2.14	(1.49 ,2.55)
Deaths	Jordan	Both	2019	0.49	(0.40 ,0.60)
Deaths	Kazakhstan	Both	2019	0.21	(0.17 ,0.26)
Deaths	Kenya	Both	2019	0.92	(0.67 ,1.25)
Deaths	Kiribati	Both	2019	0.70	(0.53 ,1.20)
Deaths	Kuwait	Both	2019	1.06	(0.84 ,1.26)
Deaths	Kyrgyzstan	Both	2019	0.18	(0.14 ,0.21)
Deaths	Laos	Both	2019	0.26	(0.19 ,0.37)
Deaths	Latvia	Both	2019	0.97	(0.79 ,1.18)
Deaths	Lebanon	Both	2019	1.11	(0.74 ,1.61)
Deaths	Lesotho	Both	2019	0.97	(0.63 ,1.48)
Deaths	Liberia	Both	2019	0.53	(0.32 ,0.82)
Deaths	Libya	Both	2019	0.75	(0.43 ,1.07)

Deaths	Lithuania	Both	2019	0.75	(0.58 ,0.93)
Deaths	Luxembourg	Both	2019	4.71	(3.78 ,5.57)
Deaths	Macedonia	Both	2019	0.75	(0.59 ,0.93)
Deaths	Madagascar	Both	2019	1.17	(0.81 ,1.58)
Deaths	Malawi	Both	2019	0.91	(0.65 ,1.27)
Deaths	Malaysia	Both	2019	0.52	(0.40 ,0.66)
Deaths	Maldives	Both	2019	0.65	(0.51 ,0.83)
Deaths	Mali	Both	2019	0.54	(0.32 ,0.87)
Deaths	Malta	Both	2019	1.85	(1.53 ,2.15)
Deaths	Marshall Islands	Both	2019	1.10	(0.72 ,1.61)
Deaths	Mauritania	Both	2019	0.49	(0.35 ,0.69)
Deaths	Mauritius	Both	2019	0.57	(0.46 ,0.72)
Deaths	Mexico	Both	2019	0.73	(0.61 ,0.87)
Deaths	Micronesia	Both	2019	1.11	(0.70 ,1.65)
Deaths	Monaco	Both	2019	1.01	(0.76 ,1.31)
Deaths	Mongolia	Both	2019	0.27	(0.19 ,0.45)
Deaths	Montenegro	Both	2019	0.49	(0.40 ,0.61)
Deaths	Morocco	Both	2019	1.03	(0.69 ,1.37)
Deaths	Mozambique	Both	2019	1.12	(0.80 ,1.57)
Deaths	Myanmar	Both	2019	0.25	(0.19 ,0.34)
Deaths	Namibia	Both	2019	0.91	(0.67 ,1.21)
Deaths	Nauru	Both	2019	1.24	(0.81 ,1.74)
Deaths	Nepal	Both	2019	0.89	(0.61 ,1.27)
Deaths	Netherlands	Both	2019	5.26	(4.15 ,6.14)
Deaths	New Zealand	Both	2019	4.79	(3.88 ,5.41)
Deaths	Nicaragua	Both	2019	0.37	(0.30 ,0.49)
Deaths	Niger	Both	2019	0.51	(0.31 ,0.81)
Deaths	Nigeria	Both	2019	0.51	(0.36 ,0.69)
Deaths	Niue	Both	2019	0.84	(0.63 ,1.10)
Deaths	North Korea	Both	2019	0.20	(0.15 ,0.28)
Deaths	Northern Mariana Islands	Both	2019	2.67	(2.08 ,3.26)
Deaths	Norway	Both	2019	5.72	(4.56 ,6.45)
Deaths	Oman	Both	2019	1.49	(1.05 ,1.95)
Deaths	Pakistan	Both	2019	0.92	(0.61 ,1.22)
Deaths	Palau	Both	2019	0.25	(0.20 ,0.33)
Deaths	Palestine	Both	2019	0.32	(0.26 ,0.40)
Deaths	Panama	Both	2019	0.49	(0.37 ,0.64)
Deaths	Papua New Guinea	Both	2019	0.71	(0.43 ,1.09)
Deaths	Paraguay	Both	2019	2.20	(1.64 ,2.82)
Deaths	Peru	Both	2019	0.30	(0.22 ,0.41)
Deaths	Philippines	Both	2019	0.20	(0.16 ,0.24)
Deaths	Poland	Both	2019	2.05	(1.52 ,2.61)
Deaths	Portugal	Both	2019	3.18	(2.61 ,3.59)
Deaths	Puerto Rico	Both	2019	1.14	(0.87 ,1.45)
Deaths	Qatar	Both	2019	0.96	(0.72 ,1.27)
Deaths	Republic of Congo	Both	2019	1.15	(0.87 ,1.57)
Deaths	Republic of Moldova	Both	2019	0.16	(0.13 ,0.21)
Deaths	Romania	Both	2019	0.77	(0.61 ,0.94)
Deaths	Russia	Both	2019	0.54	(0.44 ,0.66)
Deaths	Rwanda	Both	2019	1.06	(0.72 ,1.57)
Deaths	Saint Kitts	Both	2019	1.09	(0.91 ,1.36)

Deaths	Saint Lucia	Both	2019	0.94	(0.78 ,1.13)
Deaths	Saint Vincent and the Grenac	Both	2019	1.11	(0.93 ,1.37)
Deaths	Samoa	Both	2019	0.85	(0.64 ,1.16)
Deaths	San Marino	Both	2019	2.37	(1.55 ,3.37)
Deaths	Sao Tome and Principe	Both	2019	0.58	(0.41 ,0.78)
Deaths	Saudi Arabia	Both	2019	0.65	(0.48 ,0.84)
Deaths	Senegal	Both	2019	0.51	(0.35 ,0.71)
Deaths	Serbia	Both	2019	2.84	(2.20 ,3.70)
Deaths	Seychelles	Both	2019	0.70	(0.56 ,0.86)
Deaths	Sierra Leone	Both	2019	0.54	(0.33 ,0.82)
Deaths	Singapore	Both	2019	0.49	(0.38 ,0.59)
Deaths	Slovakia	Both	2019	1.48	(1.12 ,1.89)
Deaths	Slovenia	Both	2019	6.77	(4.92 ,8.94)
Deaths	Solomon Islands	Both	2019	0.89	(0.54 ,1.35)
Deaths	Somalia	Both	2019	0.83	(0.52 ,1.31)
Deaths	South Africa	Both	2019	0.96	(0.79 ,1.09)
Deaths	South Korea	Both	2019	0.83	(0.67 ,0.98)
Deaths	South Sudan	Both	2019	0.67	(0.44 ,0.98)
Deaths	Spain	Both	2019	4.00	(3.30 ,4.68)
Deaths	Sri Lanka	Both	2019	0.63	(0.46 ,0.86)
Deaths	Sudan	Both	2019	0.88	(0.59 ,1.27)
Deaths	Suriname	Both	2019	0.85	(0.68 ,1.06)
Deaths	Swaziland	Both	2019	0.86	(0.57 ,1.25)
Deaths	Sweden	Both	2019	4.39	(3.63 ,5.06)
Deaths	Switzerland	Both	2019	3.18	(2.52 ,3.71)
Deaths	Syrian	Both	2019	1.81	(1.28 ,2.57)
Deaths	Taiwan	Both	2019	0.91	(0.69 ,1.16)
Deaths	Tajikistan	Both	2019	0.21	(0.17 ,0.27)
Deaths	Tanzania	Both	2019	1.06	(0.70 ,1.49)
Deaths	Thailand	Both	2019	0.37	(0.27 ,0.49)
Deaths	Timor-Leste	Both	2019	0.24	(0.16 ,0.36)
Deaths	Togo	Both	2019	0.63	(0.39 ,0.98)
Deaths	Tokelau	Both	2019	0.80	(0.61 ,1.09)
Deaths	Tonga	Both	2019	0.76	(0.55 ,1.02)
Deaths	Trinidad	Both	2019	0.60	(0.45 ,0.78)
Deaths	Tunisia	Both	2019	0.92	(0.59 ,1.31)
Deaths	Turkey	Both	2019	1.03	(0.76 ,1.32)
Deaths	Turkmenistan	Both	2019	0.19	(0.15 ,0.25)
Deaths	Tuvalu	Both	2019	0.93	(0.66 ,1.29)
Deaths	Uganda	Both	2019	0.89	(0.63 ,1.26)
Deaths	UK	Both	2019	3.62	(3.09 ,3.94)
Deaths	Ukraine	Both	2019	0.25	(0.21 ,0.30)
Deaths	United Arab Emirates	Both	2019	0.95	(0.52 ,1.47)
Deaths	Uruguay	Both	2019	5.18	(4.38 ,5.88)
Deaths	USA	Both	2019	3.73	(3.11 ,4.12)
Deaths	Uzbekistan	Both	2019	0.46	(0.33 ,0.66)
Deaths	Vanuatu	Both	2019	0.89	(0.58 ,1.23)
Deaths	Venezuela	Both	2019	0.97	(0.72 ,1.30)
Deaths	Vietnam	Both	2019	0.29	(0.22 ,0.41)
Deaths	Virgin Islands	Both	2019	2.05	(1.70 ,2.49)
Deaths	Yemen	Both	2019	0.93	(0.64 ,1.28)

Deaths	Zambia	Both	2019	1.27	(0.95 ,1.67)
Deaths	Zimbabwe	Both	2019	0.90	(0.65 ,1.28)
DALYs	Afghanistan	Both	2019	21.27	(10.74 ,33.83)
DALYs	Albania	Both	2019	11.56	(8.39 ,15.37)
DALYs	Algeria	Both	2019	21.12	(13.79 ,28.87)
DALYs	American Samoa	Both	2019	23.53	(18.83 ,28.90)
DALYs	Andorra	Both	2019	26.72	(19.84 ,35.11)
DALYs	Angola	Both	2019	19.74	(15.01 ,26.06)
DALYs	Antigua	Both	2019	17.78	(14.59 ,22.40)
DALYs	Argentina	Both	2019	57.86	(52.02 ,63.25)
DALYs	Armenia	Both	2019	7.55	(6.30 ,8.97)
DALYs	Australia	Both	2019	41.11	(35.26 ,47.46)
DALYs	Austria	Both	2019	58.84	(50.90 ,67.64)
DALYs	Azerbaijan	Both	2019	4.98	(3.94 ,6.28)
DALYs	Bahamas	Both	2019	21.49	(17.38 ,27.21)
DALYs	Bahrain	Both	2019	21.57	(17.00 ,27.14)
DALYs	Bangladesh	Both	2019	14.78	(9.74 ,21.06)
DALYs	Barbados	Both	2019	23.49	(19.23 ,28.46)
DALYs	Belarus	Both	2019	6.11	(4.69 ,8.00)
DALYs	Belgium	Both	2019	59.52	(49.61 ,66.56)
DALYs	Belize	Both	2019	11.36	(9.27 ,14.55)
DALYs	Benin	Both	2019	14.81	(8.50 ,23.48)
DALYs	Bermuda	Both	2019	69.18	(57.44 ,84.51)
DALYs	Bhutan	Both	2019	15.37	(10.31 ,21.37)
DALYs	Bolivia	Both	2019	18.54	(12.93 ,26.05)
DALYs	Bosnia and Herzegovina	Both	2019	24.66	(19.24 ,31.55)
DALYs	Botswana	Both	2019	16.70	(11.24 ,25.55)
DALYs	Brazil	Both	2019	29.20	(26.27 ,33.44)
DALYs	Brunei Darussalam	Both	2019	49.33	(40.45 ,57.24)
DALYs	Bulgaria	Both	2019	14.32	(10.91 ,18.36)
DALYs	Burkina Faso	Both	2019	17.72	(10.03 ,27.60)
DALYs	Burundi	Both	2019	15.33	(10.27 ,22.05)
DALYs	Cabo Verde	Both	2019	16.76	(13.23 ,21.63)
DALYs	Cambodia	Both	2019	4.54	(3.30 ,7.07)
DALYs	Cameroon	Both	2019	17.17	(10.31 ,27.69)
DALYs	Canada	Both	2019	37.11	(32.16 ,40.74)
DALYs	Central African Republic	Both	2019	20.84	(11.36 ,32.88)
DALYs	Chad	Both	2019	13.62	(7.54 ,22.27)
DALYs	Chile	Both	2019	27.65	(24.67 ,31.54)
DALYs	China	Both	2019	4.09	(3.32 ,4.93)
DALYs	Colombia	Both	2019	27.75	(19.88 ,38.91)
DALYs	Comoros	Both	2019	15.42	(11.19 ,20.97)
DALYs	Cook Islands	Both	2019	9.04	(7.13 ,11.25)
DALYs	Costa Rica	Both	2019	28.45	(21.73 ,36.52)
DALYs	Croatia	Both	2019	52.25	(38.10 ,66.05)
DALYs	Cuba	Both	2019	23.68	(17.91 ,31.98)
DALYs	Cyprus	Both	2019	96.09	(82.40 ,111.43)
DALYs	Czechia	Both	2019	46.90	(35.79 ,59.42)
DALYs	Democratic Republic of the C	Both	2019	18.35	(11.78 ,27.38)
DALYs	Denmark	Both	2019	54.88	(47.22 ,61.42)
DALYs	Djibouti	Both	2019	18.14	(12.25 ,26.66)

DALYs	Dominica	Both	2019	21.85	(16.71 ,28.27)
DALYs	Dominican Republic	Both	2019	17.96	(13.45 ,23.65)
DALYs	Ecuador	Both	2019	19.66	(15.01 ,25.26)
DALYs	Egypt	Both	2019	27.43	(19.63 ,38.90)
DALYs	El Salvador	Both	2019	7.20	(5.33 ,9.48)
DALYs	Equatorial Guinea	Both	2019	19.96	(12.78 ,29.04)
DALYs	Eritrea	Both	2019	17.92	(11.75 ,26.67)
DALYs	Estonia	Both	2019	46.34	(34.76 ,60.71)
DALYs	Ethiopia	Both	2019	12.56	(9.15 ,17.29)
DALYs	Fiji	Both	2019	9.35	(7.33 ,11.82)
DALYs	Finland	Both	2019	66.09	(57.31 ,75.68)
DALYs	France	Both	2019	47.76	(40.45 ,53.44)
DALYs	Gabon	Both	2019	22.71	(17.19 ,29.54)
DALYs	Gambia	Both	2019	15.13	(9.58 ,22.97)
DALYs	Georgia	Both	2019	14.79	(10.79 ,22.00)
DALYs	Germany	Both	2019	67.99	(59.54 ,75.86)
DALYs	Ghana	Both	2019	13.62	(8.44 ,21.87)
DALYs	Greece	Both	2019	45.23	(39.85 ,52.25)
DALYs	Greenland	Both	2019	60.20	(37.11 ,84.43)
DALYs	Grenada	Both	2019	24.67	(20.41 ,32.06)
DALYs	Guam	Both	2019	40.66	(33.21 ,49.00)
DALYs	Guatemala	Both	2019	7.07	(5.44 ,9.12)
DALYs	Guinea	Both	2019	15.50	(8.92 ,24.68)
DALYs	Guinea-Bissau	Both	2019	18.64	(9.28 ,32.41)
DALYs	Guyana	Both	2019	46.48	(35.06 ,61.50)
DALYs	Haiti	Both	2019	25.51	(14.35 ,40.74)
DALYs	Honduras	Both	2019	15.90	(12.49 ,20.31)
DALYs	Hungary	Both	2019	75.23	(60.38 ,89.89)
DALYs	Iceland	Both	2019	49.42	(42.05 ,55.92)
DALYs	India	Both	2019	13.33	(9.88 ,17.60)
DALYs	Indonesia	Both	2019	6.11	(4.13 ,8.51)
DALYs	Iran	Both	2019	20.63	(18.56 ,23.89)
DALYs	Iraq	Both	2019	8.52	(5.73 ,11.35)
DALYs	Ireland	Both	2019	42.15	(37.07 ,47.64)
DALYs	Israel	Both	2019	43.78	(37.54 ,49.17)
DALYs	Italy	Both	2019	34.69	(28.94 ,39.17)
DALYs	Ivory Coast	Both	2019	13.91	(8.24 ,21.67)
DALYs	Jamaica	Both	2019	7.42	(5.72 ,9.82)
DALYs	Japan	Both	2019	26.55	(21.26 ,30.40)
DALYs	Jordan	Both	2019	10.10	(8.25 ,12.37)
DALYs	Kazakhstan	Both	2019	5.67	(4.59 ,7.30)
DALYs	Kenya	Both	2019	16.10	(12.50 ,20.78)
DALYs	Kiribati	Both	2019	16.95	(12.01 ,26.39)
DALYs	Kuwait	Both	2019	17.90	(14.75 ,21.25)
DALYs	Kyrgyzstan	Both	2019	4.50	(3.66 ,5.47)
DALYs	Laos	Both	2019	5.49	(3.97 ,8.06)
DALYs	Latvia	Both	2019	23.60	(18.84 ,29.72)
DALYs	Lebanon	Both	2019	26.75	(16.79 ,40.25)
DALYs	Lesotho	Both	2019	17.82	(11.05 ,28.49)
DALYs	Liberia	Both	2019	13.21	(7.72 ,20.93)
DALYs	Libya	Both	2019	18.54	(11.25 ,26.54)

DALYs	Lithuania	Both	2019	20.01	(15.63 ,24.93)
DALYs	Luxembourg	Both	2019	60.92	(51.18 ,71.65)
DALYs	Macedonia	Both	2019	15.33	(12.18 ,19.04)
DALYs	Madagascar	Both	2019	24.22	(16.13 ,34.01)
DALYs	Malawi	Both	2019	16.81	(12.25 ,22.75)
DALYs	Malaysia	Both	2019	10.21	(7.91 ,13.16)
DALYs	Maldives	Both	2019	11.56	(9.19 ,14.32)
DALYs	Mali	Both	2019	12.90	(7.29 ,21.99)
DALYs	Malta	Both	2019	27.01	(22.89 ,31.14)
DALYs	Marshall Islands	Both	2019	23.29	(14.36 ,35.29)
DALYs	Mauritania	Both	2019	11.70	(7.96 ,17.46)
DALYs	Mauritius	Both	2019	15.56	(12.28 ,19.71)
DALYs	Mexico	Both	2019	16.30	(13.51 ,19.42)
DALYs	Micronesia	Both	2019	24.43	(14.18 ,38.62)
DALYs	Monaco	Both	2019	16.91	(13.26 ,21.17)
DALYs	Mongolia	Both	2019	7.33	(5.14 ,12.01)
DALYs	Montenegro	Both	2019	12.62	(10.17 ,16.09)
DALYs	Morocco	Both	2019	21.77	(15.39 ,29.22)
DALYs	Mozambique	Both	2019	21.08	(14.55 ,28.62)
DALYs	Myanmar	Both	2019	5.35	(4.04 ,7.69)
DALYs	Namibia	Both	2019	15.65	(11.57 ,21.76)
DALYs	Nauru	Both	2019	27.41	(17.24 ,40.73)
DALYs	Nepal	Both	2019	15.32	(10.78 ,20.95)
DALYs	Netherlands	Both	2019	60.00	(49.94 ,68.18)
DALYs	New Zealand	Both	2019	63.13	(54.19 ,70.31)
DALYs	Nicaragua	Both	2019	8.42	(6.65 ,10.93)
DALYs	Niger	Both	2019	12.56	(7.08 ,20.52)
DALYs	Nigeria	Both	2019	11.68	(7.90 ,16.99)
DALYs	Niue	Both	2019	18.93	(14.10 ,25.38)
DALYs	North Korea	Both	2019	5.49	(3.96 ,7.86)
DALYs	Northern Mariana Islands	Both	2019	56.46	(45.35 ,69.14)
DALYs	Norway	Both	2019	64.27	(53.86 ,71.40)
DALYs	Oman	Both	2019	28.65	(19.46 ,39.02)
DALYs	Pakistan	Both	2019	17.22	(11.00 ,23.67)
DALYs	Palau	Both	2019	6.99	(5.39 ,9.03)
DALYs	Palestine	Both	2019	7.51	(6.13 ,9.52)
DALYs	Panama	Both	2019	11.89	(8.99 ,15.76)
DALYs	Papua New Guinea	Both	2019	14.53	(8.56 ,22.46)
DALYs	Paraguay	Both	2019	42.55	(31.44 ,55.98)
DALYs	Peru	Both	2019	7.56	(5.50 ,10.22)
DALYs	Philippines	Both	2019	4.31	(3.48 ,5.27)
DALYs	Poland	Both	2019	37.70	(28.53 ,47.55)
DALYs	Portugal	Both	2019	44.28	(38.56 ,49.42)
DALYs	Puerto Rico	Both	2019	23.79	(18.72 ,29.71)
DALYs	Qatar	Both	2019	16.32	(12.27 ,21.65)
DALYs	Republic of Congo	Both	2019	21.02	(14.99 ,29.69)
DALYs	Republic of Moldova	Both	2019	6.88	(5.40 ,9.07)
DALYs	Romania	Both	2019	25.39	(19.77 ,33.98)
DALYs	Russia	Both	2019	16.11	(12.86 ,20.07)
DALYs	Rwanda	Both	2019	18.85	(13.36 ,27.14)
DALYs	Saint Kitts	Both	2019	21.01	(16.54 ,26.94)

DALYs	Saint Lucia	Both	2019	19.56	(16.12 ,24.09)
DALYs	Saint Vincent and the Grenac	Both	2019	23.11	(19.13 ,29.03)
DALYs	Samoa	Both	2019	18.10	(13.22 ,24.94)
DALYs	San Marino	Both	2019	30.56	(20.74 ,43.90)
DALYs	Sao Tome and Principe	Both	2019	14.20	(9.83 ,19.12)
DALYs	Saudi Arabia	Both	2019	13.48	(10.27 ,17.58)
DALYs	Senegal	Both	2019	12.61	(8.36 ,18.17)
DALYs	Serbia	Both	2019	47.37	(37.43 ,59.63)
DALYs	Seychelles	Both	2019	14.18	(11.37 ,17.13)
DALYs	Sierra Leone	Both	2019	13.79	(8.21 ,20.97)
DALYs	Singapore	Both	2019	11.91	(9.57 ,14.87)
DALYs	Slovakia	Both	2019	31.18	(23.63 ,39.19)
DALYs	Slovenia	Both	2019	91.55	(70.09 ,118.18)
DALYs	Solomon Islands	Both	2019	19.92	(10.63 ,33.07)
DALYs	Somalia	Both	2019	16.27	(9.58 ,26.03)
DALYs	South Africa	Both	2019	17.17	(14.02 ,19.67)
DALYs	South Korea	Both	2019	13.25	(11.11 ,15.77)
DALYs	South Sudan	Both	2019	12.01	(7.90 ,17.57)
DALYs	Spain	Both	2019	52.55	(45.60 ,59.91)
DALYs	Sri Lanka	Both	2019	13.83	(10.02 ,18.96)
DALYs	Sudan	Both	2019	20.70	(13.64 ,30.94)
DALYs	Suriname	Both	2019	18.54	(14.80 ,23.72)
DALYs	Swaziland	Both	2019	16.07	(10.25 ,24.76)
DALYs	Sweden	Both	2019	52.66	(46.19 ,58.97)
DALYs	Switzerland	Both	2019	38.09	(32.50 ,43.27)
DALYs	Syrian	Both	2019	37.96	(26.75 ,53.41)
DALYs	Taiwan	Both	2019	13.33	(10.28 ,17.07)
DALYs	Tajikistan	Both	2019	5.08	(4.04 ,6.34)
DALYs	Tanzania	Both	2019	19.10	(13.28 ,27.13)
DALYs	Thailand	Both	2019	6.95	(5.16 ,9.24)
DALYs	Timor-Leste	Both	2019	4.89	(3.02 ,7.56)
DALYs	Togo	Both	2019	15.77	(9.22 ,25.57)
DALYs	Tokelau	Both	2019	17.25	(12.81 ,23.90)
DALYs	Tonga	Both	2019	16.13	(11.57 ,21.98)
DALYs	Trinidad	Both	2019	14.09	(10.35 ,18.97)
DALYs	Tunisia	Both	2019	20.38	(12.77 ,29.56)
DALYs	Turkey	Both	2019	19.24	(14.48 ,24.50)
DALYs	Turkmenistan	Both	2019	5.16	(4.05 ,6.76)
DALYs	Tuvalu	Both	2019	20.32	(14.39 ,28.59)
DALYs	Uganda	Both	2019	16.33	(12.15 ,22.20)
DALYs	UK	Both	2019	50.78	(45.49 ,55.33)
DALYs	Ukraine	Both	2019	8.23	(6.66 ,10.13)
DALYs	United Arab Emirates	Both	2019	24.93	(13.93 ,37.82)
DALYs	Uruguay	Both	2019	73.15	(65.09 ,81.49)
DALYs	USA	Both	2019	47.99	(42.61 ,52.83)
DALYs	Uzbekistan	Both	2019	9.37	(7.01 ,13.08)
DALYs	Vanuatu	Both	2019	19.23	(12.23 ,28.18)
DALYs	Venezuela	Both	2019	21.35	(15.53 ,29.40)
DALYs	Vietnam	Both	2019	6.08	(4.40 ,8.72)
DALYs	Virgin Islands	Both	2019	43.12	(35.12 ,52.17)
DALYs	Yemen	Both	2019	21.61	(14.46 ,30.96)

DALYs	Zambia	Both	2019	23.45	(17.21 ,31.97)
DALYs	Zimbabwe	Both	2019	17.03	(12.37 ,24.34)
